# Ancient Origin of the CARD–Coiled Coil/Bcl10/MALT1-Like Paracaspase Signaling Complex Indicates Unknown Critical Functions

**DOI:** 10.3389/fimmu.2018.01136

**Published:** 2018-05-24

**Authors:** Jens Staal, Yasmine Driege, Mira Haegman, Alice Borghi, Paco Hulpiau, Laurens Lievens, Ismail Sahin Gul, Srividhya Sundararaman, Amanda Gonçalves, Ineke Dhondt, Jorge H. Pinzón, Bart P. Braeckman, Ulrich Technau, Yvan Saeys, Frans van Roy, Rudi Beyaert

**Affiliations:** ^1^Unit of Molecular Signal Transduction in Inflammation, VIB-UGent Center for Inflammation Research (IRC), Ghent, Belgium; ^2^Department of Biomedical Molecular Biology, Ghent University, Ghent, Belgium; ^3^Unit of Data Mining and Modeling for Biomedicine, VIB-UGent Center for Inflammation Research (IRC), Ghent, Belgium; ^4^Unit of Molecular Cell Biology, VIB-UGent Center for Inflammation Research (IRC), Ghent, Belgium; ^5^VIB Bio Imaging Core Gent, VIB-UGent Center for Inflammation Research (IRC), Ghent, Belgium; ^6^Laboratory for Aging Physiology and Molecular Evolution, Biology Department, Ghent University, Ghent, Belgium; ^7^Department of Biology, University of Texas Arlington, Arlington, TX, United States; ^8^Department of Molecular Evolution and Development, Faculty of Life Sciences, University of Vienna, Vienna, Austria; ^9^Department of Applied Mathematics, Computer Science and Statistics, Ghent University, Ghent, Belgium

**Keywords:** signal transduction, molecular evolution, protein–protein interaction, structure–function analysis, NF-kappaB, coral bleaching, *Nematostella vectensis*, *Caenorhabditis elegans*

## Abstract

The CARD–coiled coil (CC)/Bcl10/MALT1-like paracaspase (CBM) signaling complexes composed of a CARD–CC family member (CARD-9, -10, -11, or -14), Bcl10, and the type 1 paracaspase MALT1 (PCASP1) play a pivotal role in immunity, inflammation, and cancer. Targeting MALT1 proteolytic activity is of potential therapeutic interest. However, little is known about the evolutionary origin and the original functions of the CBM complex. Type 1 paracaspases originated before the last common ancestor of planulozoa (bilaterians and cnidarians). Notably in bilaterians, Ecdysozoa (e.g., nematodes and insects) lacks Bcl10, whereas other lineages have a Bcl10 homolog. A survey of invertebrate CARD–CC homologs revealed such homologs only in species with Bcl10, indicating an ancient common origin of the entire CBM complex. Furthermore, vertebrate-like Syk/Zap70 tyrosine kinase homologs with the ITAM-binding SH2 domain were only found in invertebrate organisms with CARD–CC/Bcl10, indicating that this pathway might be related to the original function of the CBM complex. Moreover, the type 1 paracaspase sequences from invertebrate organisms that have CARD–CC/Bcl10 are more similar to vertebrate paracaspases. Functional analysis of protein–protein interactions, NF-κB signaling, and CYLD cleavage for selected invertebrate type 1 paracaspase and Bcl10 homologs supports this scenario and indicates an ancient origin of the CARD–CC/Bcl10/paracaspase signaling complex. By contrast, many of the known MALT1-associated activities evolved fairly recently, indicating that unknown functions are at the basis of the protein conservation. As a proof-of-concept, we provide initial evidence for a CBM- and NF-κB-independent neuronal function of the Caenorhabditis elegans type 1 paracaspase malt-1. In conclusion, this study shows how evolutionary insights may point at alternative functions of MALT1.

## Introduction

The paracaspase MALT1 (PCASP1) was originally identified in humans as an oncogenic fusion with IAP2 in low-grade antibiotic-resistant MALT lymphomas (1). Later, it was discovered that MALT1 is a critical component in T and B cell antigen receptor signaling as part of the CARD-11–Bcl10–MALT1 (CBM) signaling complex that is formed upon antigen stimulation. Antigen receptor signaling *via* the ITAM (immunoreceptor tyrosine-based activation motif)-binding SH2 domain tyrosine kinase Zap70 activates PKC, leading to CARD-11 phosphorylation and the recruitment of a preexisting Bcl10/MALT1 complex (2–4). MALT1 in the activated CBM complex recruits critical downstream proteins, such as TRAF6, for the activation of NF-κB-dependent gene expression (5) (Figure S1A in Supplementary Material). CARD-11 (also known as CARMA1) belongs to a distinct phylogenetic group of CARD domain proteins, which is characterized by a CARD and a coiled-coil (CC) domain, and this group of proteins will thus be referred to as CARD–CC proteins. Further studies made it clear that MALT1 plays a role in several different CARD–CC/Bcl10/MALT1 signaling complexes, which are composed of specific CARD–CC family proteins [CARD-9 (6), CARD-11 (4), CARD-14 (also known as CARMA2) (7–9), and CARD-10 (also known as CARMA3) (10)] and which are formed upon the stimulation of distinct receptors in several immune and non-immune cells. The use of different CARD–CC proteins in the CBM complexes has been proposed to depend on their cell-type-specific expression (11), although other mechanisms cannot be ruled out. CARD-9, CARD-10, CARD-11, and CARD-14 are the only members of this CARD–CC family in humans, which makes it unlikely that additional similar human CBM complexes will be found. MALT1 has been annotated as a “paracaspase” due to sequence similarity with the true caspases and “metacaspases” (12). A broader survey of paracaspases in the whole tree of life indicates that “paracaspases” should be considered a sub-class of “metacaspases” and that paracaspases have evolved several times independently (13). The name caspase refers to both the structure (cysteine protease) and the function (aspartic acid substrate specificity) of the protein family. The semantic association of metacaspases and paracaspases to caspases is therefore unfortunate, since these similar names inspired false assumptions of common roles and properties of the different protein families (14). Despite the identification of “paracaspase” in 2000, it was not until 2008 that the proteolytic activity of MALT1 was established (15, 16). In contrast to true caspases (but similar to metacaspases and orthocaspases), the PCASP1 cleaves substrates specifically after an arginine residue (17–19). The anti-inflammatory role of many of the known protease substrates coupled with the critical role for MALT1 in pro-inflammatory signaling has sparked an interest in targeting MALT1 protease activity as a therapeutic strategy for autoimmune diseases (20). The proteolytic activity of MALT1 was also found to be critical for certain cancers (21), which has stimulated an interest in MALT1 protease activity as a cancer therapy target as well. Although the MALT1 scaffold function for the recruitment of downstream TRAF6 has been clearly associated to NF-κB activation (22), the MALT1 proteolytic activity plays a more subtle role being required for the activation of a subset of NF-κB-dependent genes (23–26) and the stabilization of specific mRNAs (27). MALT1 belongs to the type 1 paracaspase family, which consists of an N-terminal death domain, immunoglobulin domains, and a paracaspase domain. Type 2 paracaspases only contain the paracaspase domain and are found in non-metazoans and early-branching metazoans like porifera, placozoa, and ctenophora (13). The type 1 family of paracaspases originated sometime during the Ediacaran geological period, preceding the last common ancestor of bilaterians and cnidarians (13, 28, 29). The cnidarians (e.g., jellyfish, sea anemone, hydra, and coral) and bilaterians (e.g., vertebrates, insects, nematodes, mollusks, and ringed worms) form the planulozoan clade (30). In our previous survey of paracaspases and MALT1-associated proteins, type 1 paracaspases and Bcl10 could not be found outside planulozoa (13). Cnidarians typically contain several paralogs of both type 1 and the ancient type 2 paracaspases, whereas bilaterians typically contain a single copy of a type 1 paracaspase. Notable exceptions are the jawed vertebrates, where the type 1 paracaspase got triplicated. Subsequently, two paralogs were lost in the mammalian lineage leaving MALT1 as the single paracaspase in mammals (13). Importantly, some invertebrates, such as the nematode *Caenorhabditis elegans*, contain a conserved type 1 paracaspase but lack NF-κB (31), which indicates that other roles or mechanisms might be responsible for the conservation of the general domain organization of the type 1 paracaspases (13). Mice deficient for MALT1 do not show any obvious developmental phenotypes and primarily show defects in T and B cell functions (2, 3). In addition to functional studies of MALT1 in human cells, human patients, and mouse models, investigating the evolutionary history of the type 1 paracaspases, their interacting proteins and molecular functions in alternative model systems could provide important clues to yet-unknown roles and functions of human MALT1 (13). Finding those alternative functions of MALT1 could ultimately be important in the context of future MALT1 inhibitor-based therapies (32) and could help identify yet undiscovered issues that might affect patients deficient in MALT1.

## Results

### CBM Complex Evolution Indicates Ancient Functional Conservation

**Figure 1:**
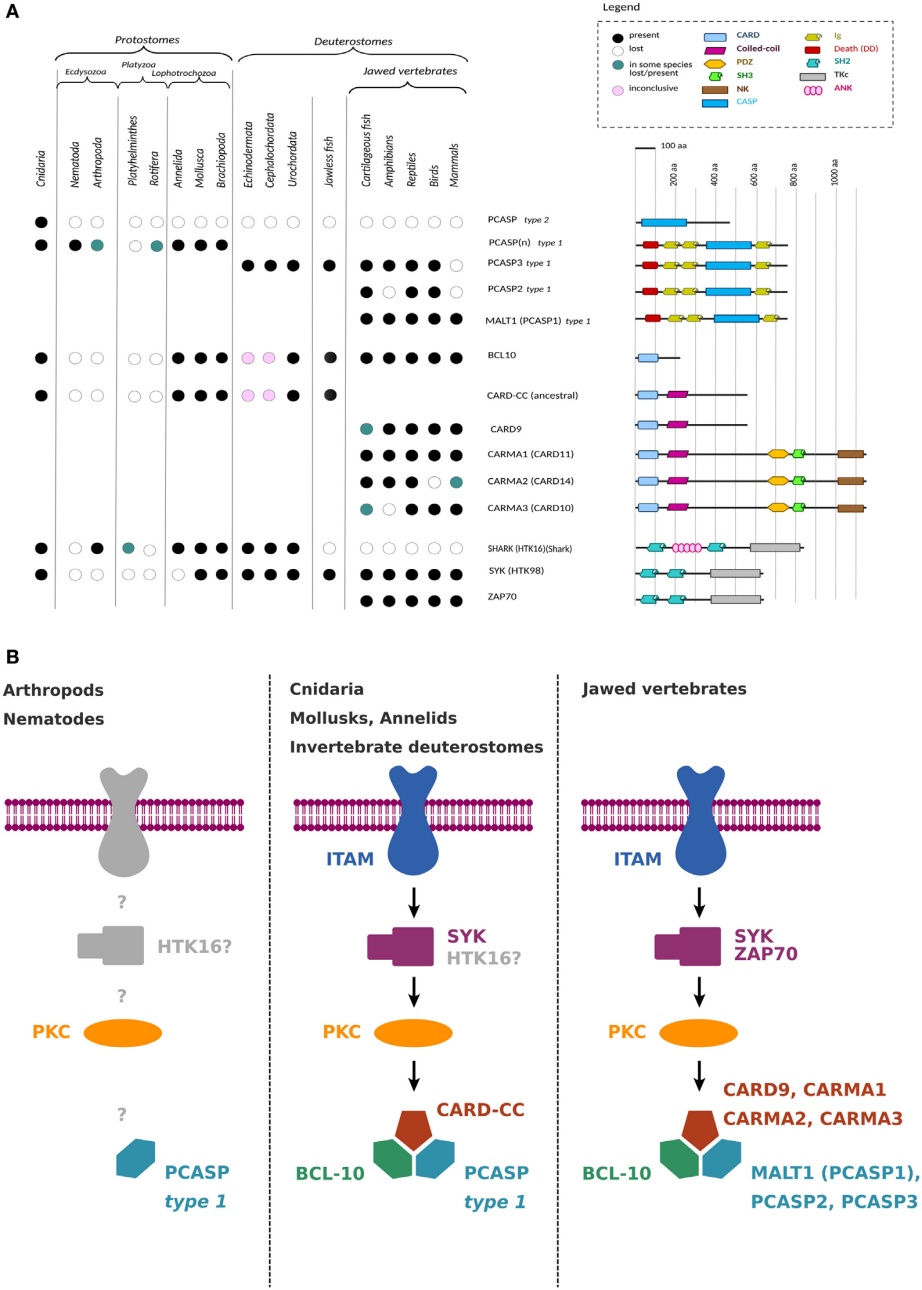
Coevolution and proposed signaling model. **(A)** Patterns of coevolution of Syk and several CBM complex components in various organisms within the planulozoan clade. Type 1 paracaspases prior to Deuterostomes were annotated as PCASP(*n*) since currently available invertebrate genome sequences cannot determine whether a distant paracaspase is an ancient PCASP3 paralog or ortholog. One model proposes two ancient type 1 paracaspases, one Bcl10-dependent and one Bcl10-independent. The CARD–CC/Bcl10-dependent type 1 paracaspase shows MALT1-like activities. Deuterostomia (including tunicates, lancelets, vertebrates, and hemichordates), annelids, and mollusks inherited the Bcl10-dependent type 1 paracaspase, whereas most other bilaterian invertebrates kept the Bcl10-independent type 1 paracaspase. The model is based on currently available reliable sequence information and might change with additional data. Analogously, CARD–CC became duplicated and later fused with MAGUK domains in the jawed vertebrates. At this moment, we are uncertain about which of the four jawed vertebrate CARD–CC paralogs (CARD-9, -10, -11, -14) should be considered the ortholog of the ancestral CARD–CC. Also, upstream Syk became duplicated in jawed vertebrates, resulting in Zap70. **(B)** Proposed signaling model in various organism classes. Nothing is known about upstream activators of type 1 paracaspases in CARD–CC/Bcl10-independent organisms such as arthropods and nematodes.

In order to unravel the evolutionary history of the CBM complex, the sequences of CARD–CC, Bcl10, and other CBM complex-associated proteins were investigated through mining of several sequence databases and through phylogenetic analyses. The CARD–CC and Bcl10 CBM complex components showed coevolution (Figure 1A), whereas MALT1-like paracaspases could be found also in organisms lacking the upstream signaling proteins Bcl10 and CARD–CC (Figure 1; Figures S1–S3 in Supplementary Material). The presence of invertebrate Syk/Zap70 homologs correlates with the presence of Bcl10 and CARD–CC (Figure 1; Figures S1B and S2 in Supplementary Material). Since the N-terminal SH2 domains in Syk and Zap70 are critical for interaction with upstream ITAM domain-containing receptors (33, 34), the overlapping phylogenetic distribution of the CBM complex and Syk/Zap70 indicates an ancient conservation of ITAM-containing upstream receptors linked to signaling *via* the CBM complex (Figure 1B; Figure S1A in Supplementary Material). By contrast, another invertebrate SH2 domain tyrosine kinase (“Shark”), which has been shown to also mediate ITAM-dependent immune-related signals (35), does not show a correlation with the phylogenetic distribution of the CBM complex components. Another evolutionary conserved class of receptors that can signal *via* a CBM complex-dependent pathway is the G protein-coupled receptors (GPCRs). Interestingly, a common feature for the currently known GPCR pathways that signal *via* a CBM complex is that they depend on G_12_/G_13_, G_q_, and RhoA (36). These components do, however, not show a pattern of CBM coevolution. In an extended analysis of homologs of human CBM-interacting proteins and comparisons to the CBM complex phylogenetic distribution, we identified proteins with phylogenetic distributions that were either not correlated (CBM-independent), correlated (CBM-coevolving), or vertebrate-specific, which might reflect on the original functions of the CBM complex: the CBM-interacting proteins AIP (37), Akt (38), CaMKII (39), caspase-8 (40), β-catenin, and its destruction complex (41), cIAP1/cIAP2 (42), CK1α (43), CRADD (44), CSN5 (45), MIB2 (46), NOTCH1 (47), p62/SQSTM1 (48), RLTPR (49), and Rubicon (50) showed a wide phylogenetic distribution with no indications of a CBM coevolution, indicating important CBM-independent roles. By contrast, A20 (15), DEPDC7 (51), HECTD3 (52), LRRK1 (53), Net1 (54), and RIG-I (55) showed a phylogenetic distribution or BLASTp ranking that correlates with the presence of the CBM complex. Other CBM-interacting proteins like ADAP (56), BINCA (57), CKIP1 (58), RIPK2 (59), and USP2a (60) were poorly conserved in invertebrates and might represent more recently evolved CBM interaction partners. Taken together, these phylogenetic patterns indicate an ancient conserved signaling pathway, possibly *via* ITAM/Syk/PKC to CBM complex activation (Figure 1B).

### Organisms With CARD–CC and Bcl10 Have More Vertebrate-Like Type 1 Paracaspase Sequences

**Figure 2:**
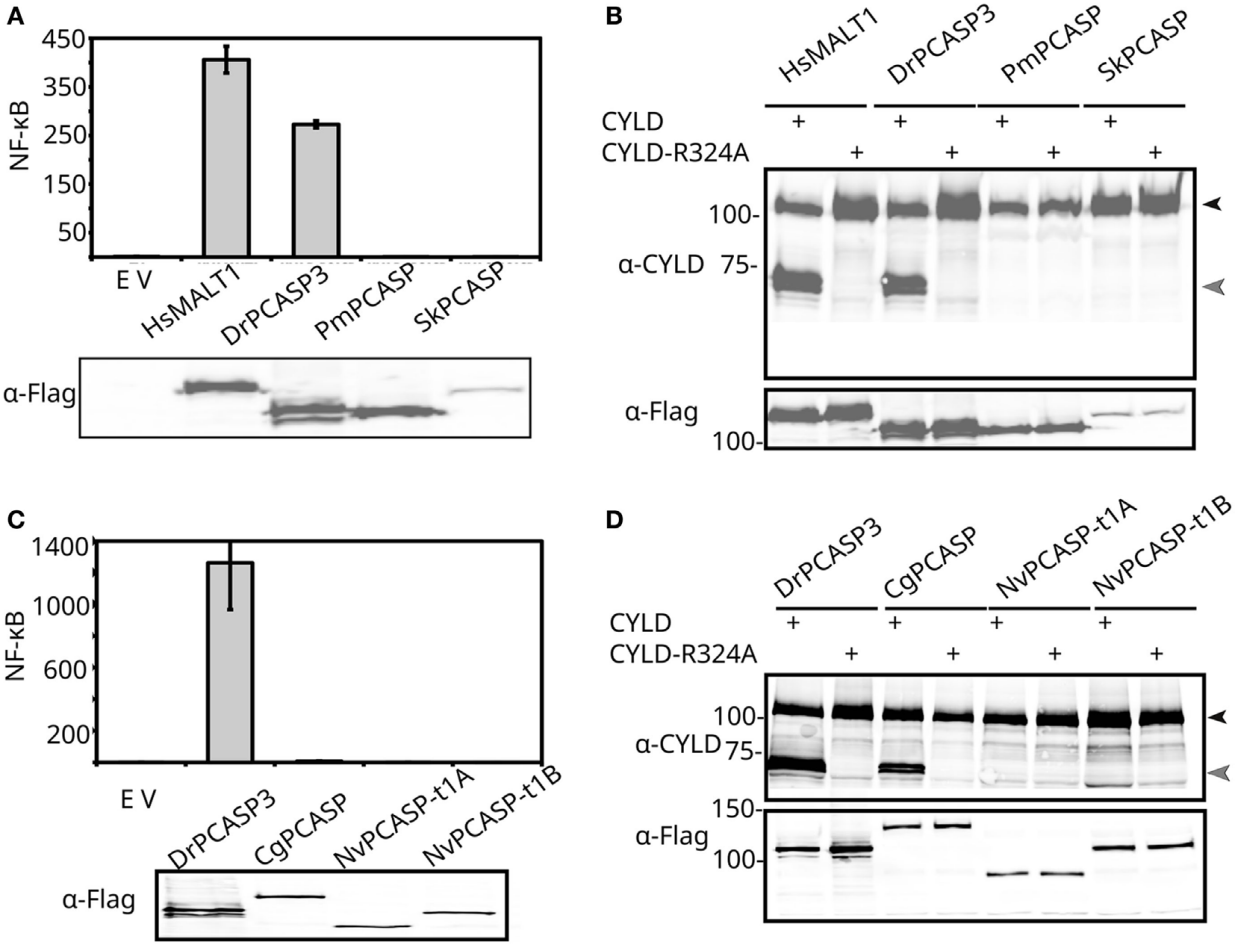
Functional conservation of invertebrate paracaspases. **(A,C)** NF-κB-dependent luciferase reporter gene induction by HLH-paracaspase fusion proteins. The indicated Flag-tagged HLH-paracaspases were expressed in MALT1-deficient HEK293T cells together with an NF-κB-dependent luciferase reporter expression plasmid and a constituitively expressed β-galactosidase reporter gene. Luciferase values are normalized against β-galactosidase and expressed as fold induction compared to samples not expressing a HLH-paracaspase (empty vector: EV). Error bars represent 95% confidence intervals [Student’s *t*-distribution (65)]. The lower part of the panel shows the expression of each HLH-paracaspase as revealed by Western blotting and development with anti-Flag antibodies. Experiments were repeated four times. **(B,D)** CYLD cleavage by HLH-paracaspase fusion proteins. MALT1-deficient HEK293T cells were transiently transfected with the indicated HLH-paracaspases together with either human WT CYLD or CYLD (R324A) in which the MALT1 cleavage site is mutated. Cell lysates were analyzed for CYLD expression and cleavage *via* Western blotting and detection with anti-CYLD antibodies. Full-length CYLD is indicated with a closed arrowhead; cleaved CYLD is indicated by an open arrowhead. To show equal expression levels of each HLH-paracaspase, the blot was also developed with anti-Flag antibodies (lower part). Experiments were repeated three times.

**Figure 3:**
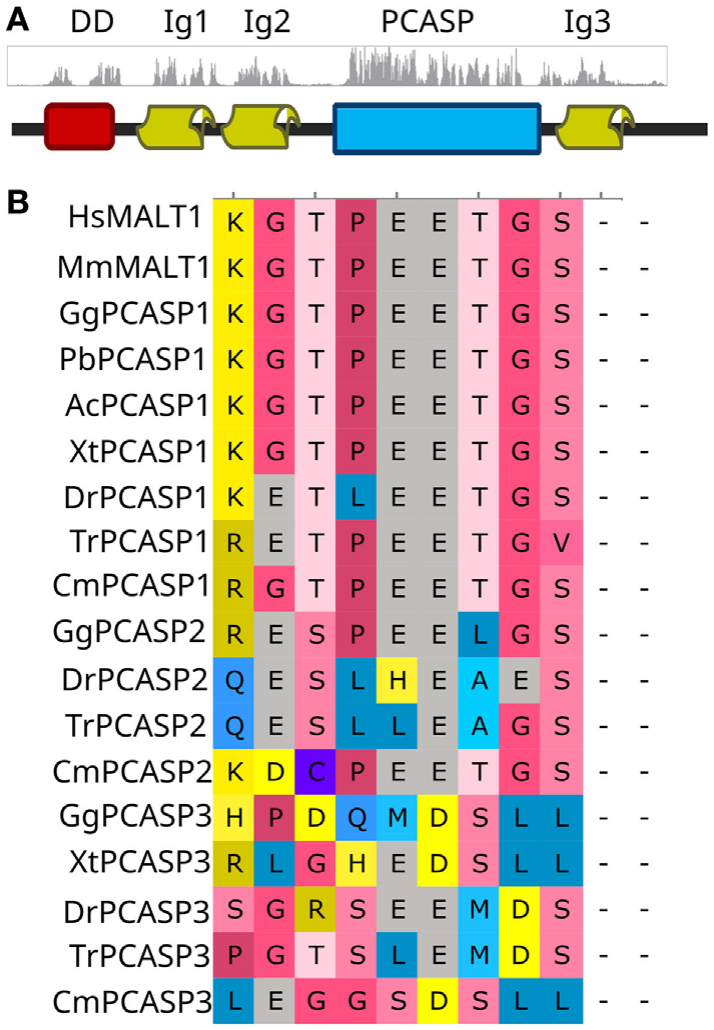
Domain conservation and TRAF6-binding evolution. **(A)** Conservation plot of a MUSCLE alignment from a wide selection of type 1 paracaspases from Cnidaria to humans. Conserved segments correspond well to annotated domains. **(B)** MUSCLE alignment segment of the most conserved TRAF6-binding site in the Ig3 domain. The corresponding sequence is missing in type 1 paracaspase homologs from organisms that diverged before the last common ancestor of jawed vertebrates.

**Figure 4:**
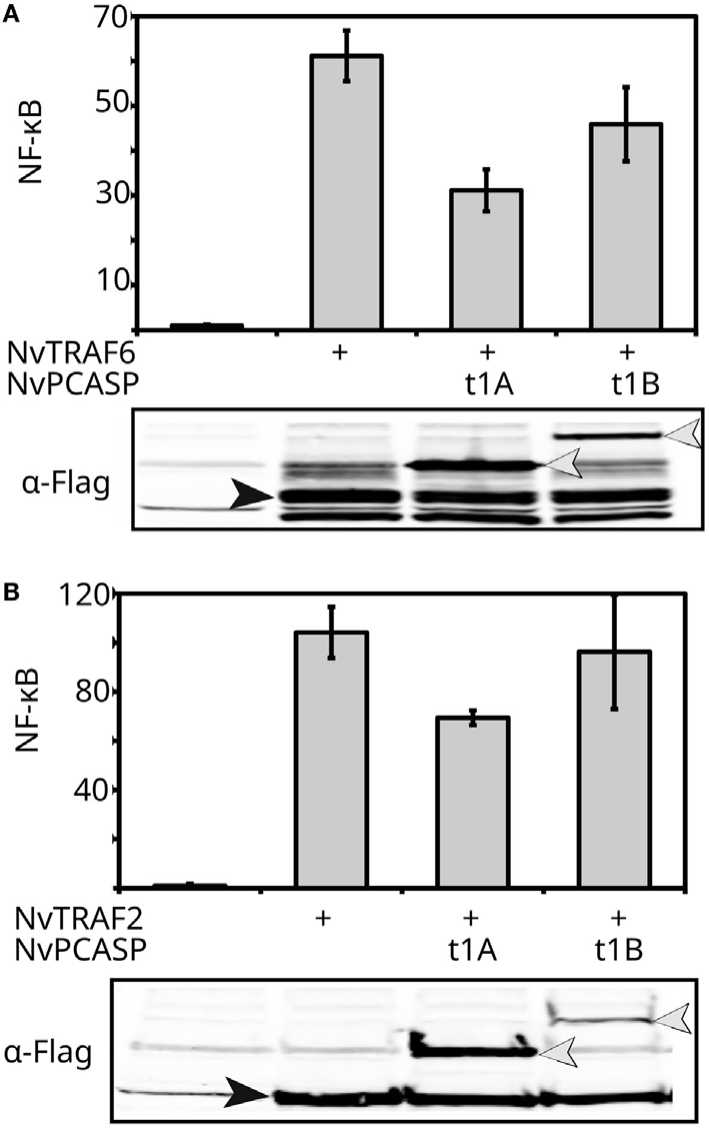
Functional conservation of cnidarian TRAF6 and TRAF2. **(A,B)** *Nematostella vectensis* (Nvn) TRAF6 **(A)** or TRAF2 **(B)** was expressed in human HEK293T cells together with an NF-κB-dependent luciferase reporter gene and a constitutively expressed β-galactosidase reporter gene plasmids. The TRAF was transfected either alone or together with the indicated Nvn HLH-paracaspase fusion protein. Luciferase values were normalized against β-galactosidase and expressed as fold induction compared to samples not expressing the TRAF. Error bars represent 95% confidence intervals (Student’s *t*-distribution). The lower part of the panel shows the expression of the TRAF (closed arrow head) and each Flag-tagged HLH-fused type 1 paracaspase (open arrow heads) as revealed by Western blotting and development with anti-Flag antibodies. Experiments were repeated at least twice.

**Figure 5:**
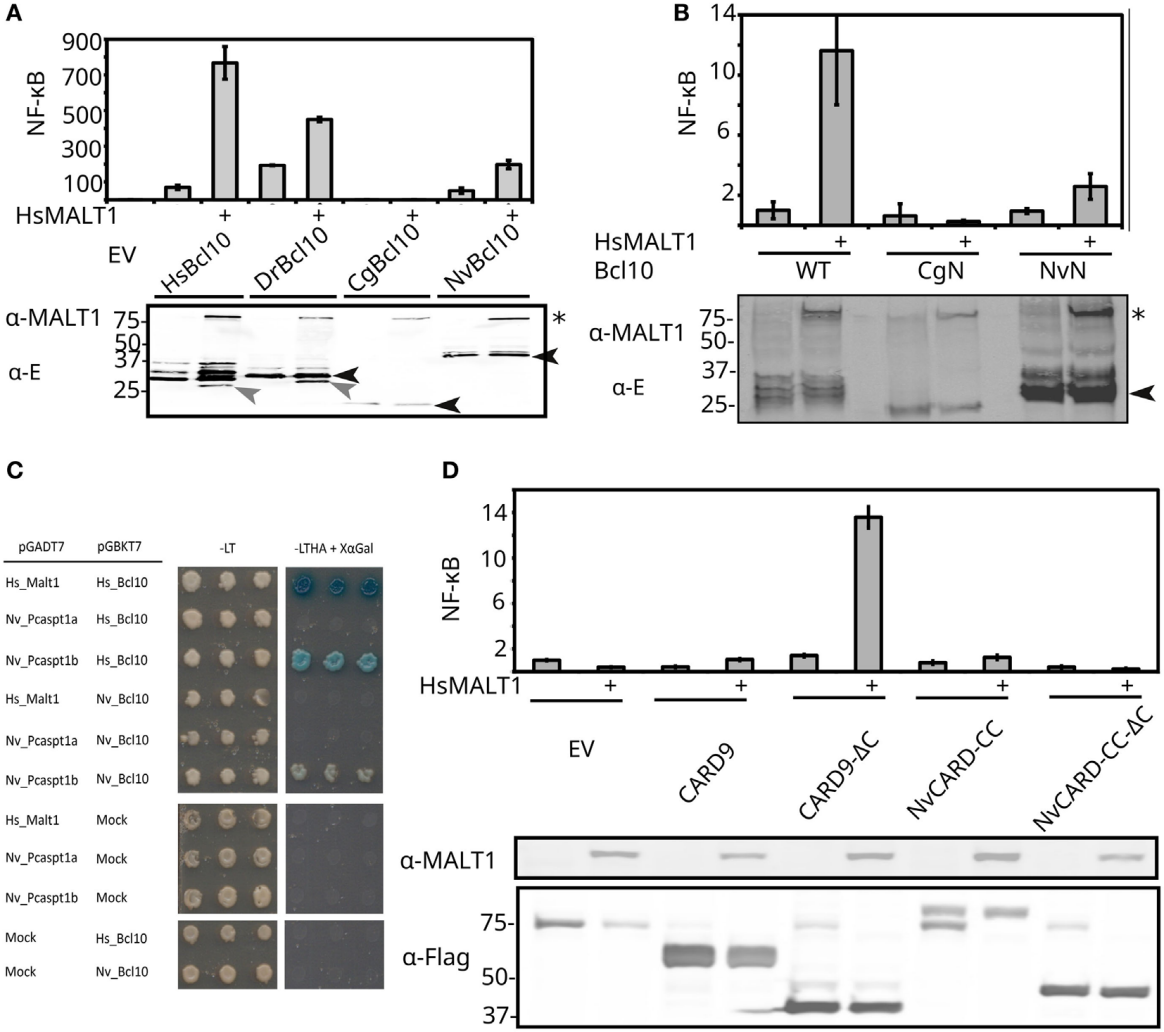
Functional conservation of Bcl10 and CARD–CC proteins. **(A)** Human MALT1-dependent NF-κB induction by different Bcl10 homologs. The indicated E-tagged Bcl10 homologs were expressed in MALT1-deficient HEK293T cells together with an NF-κB-dependent luciferase reporter expression plasmid and a constituitively expressed β-galactosidase reporter gene. Where indicated (+), human MALT1 was also co-expressed. Luciferase values were normalized against β-galactosidase and expressed as fold induction compared to samples not expressing Bcl10 (EV). Error bars represent 95% confidence intervals (Student’s *t*-distribution). The expression of E-tagged Bcl10 homologs and human MALT1 was revealed by Western blotting and detection with anti-E-tag or anti-MALT1 antibodies, respectively (lower part). Full-length Bcl10 is indicated by closed arrow heads and cleaved Bcl10 by open arrow heads. MALT1 is indicated by an asterisk. Experiments were repeated two times. **(B)** Bcl10/CARD-dependent MALT1-mediated NF-κB induction. Wild-type human Bcl10 and hybrid Bcl10 clones where residues 1–102 of human Bcl10 were replaced by the corresponding residues from *C. gigas* (CgN) or *Nematostella vectensis* (NvN) Bcl10 were expressed in MALT1-deficient HEK293T cells together with an NF-κB-dependent luciferase reporter expression plasmid and a constituitively expressed β-galactosidase reporter gene. Where indicated, (+) human MALT1 was also co-expressed. Luciferase values were normalized against β-galactosidase and expressed as fold induction compared to samples expressing human wild-type Bcl10 without MALT1. Experiments were repeated two times. **(C)** Yeast-2-hybrid assay demonstrating conserved interaction between both Nvn and human Bcl10 and type 1 paracaspases. Left panel represents growth on non-selective media and right panel selective growth (-LTHA medium), on which only a combination of bait and prey clones with interacting proteins can grow. As an independent readout, clones with strong bait–prey interactions also stain blue from X-gal. **(D)** Full-length and C-terminal deletion (ΔC) constructs of human CARD-9 and Nvn CARD–CC were expressed in MALT1-deficient HEK293T cells together with NF-κB-dependent luciferase reporter gene and constitutively expressed β-galactosidase reporter gene plasmids. Where indicated, (+) human MALT1 was also co-expressed. Luciferase values are normalized against β-galactosidase and expressed as fold induction compared to samples not expressing CARD-9 or CARD–CC (EV). Error bars represent 95% confidence intervals (Student’s *t*-distribution). The lower part of the panel shows the expression of full-length and ΔC-mutant CARD-9 and Nvn CARD–CC constructs, respectively, as revealed by Western blotting and development with anti-Flag antibodies. An aspecific Flag signal at 75 kDa is sometimes visible in the MALT1-deficient HEK293T cells. Experiments were repeated two times.

While searching for invertebrate homologs of type 1 paracaspases and Bcl10, it became apparent that type 1 paracaspases from species containing Syk, CARD–CC, and Bcl10 (Figure 1; Figures S1B and S2 in Supplementary Material) generally had higher BLASTp scores compared to species from phyla lacking these CBM complex-associated proteins. Phylogenetic analyses of several type 1 paracaspases revealed that type 1 paracaspases from species that contain *Bcl10* (cnidarians, mollusks, annelids, hemichordates) often cluster closer to the vertebrate paracaspases, either directly or indirectly by clustering with the invertebrate *Pcasp3* orthologs from tunicate and lancelet (13) (Figures S1C and S3 in Supplementary Material), indicating a conserved common Bcl10-dependent paracaspase ancestor. Since the MALT1 N-terminal death domain and immunoglobulin domain are Bcl10 binding (61), a phylogenetic analysis of the sequence N-terminal of the caspase-like domain was performed. This analysis showed a stronger association between paracaspases from *Bcl10*-containing species, but does not cluster known paralogs within the deuterostomes (Figure S1C in Supplementary Material), such as PCASP3 protein sequences from acorn worm, lancelet, or tunicate. Phylogenetic analyses of full-length paracaspases and of the highly conserved caspase-like domain indicate that the last common bilaterian ancestor had two to three different type 1 paracaspases (Figure S3 in Supplementary Material), which makes it possible that Bcl10-dependent and -independent paracaspases originated before the bilaterians. This is intriguingly similar to the early evolution of the apoptosis network, where the last planulozoan ancestor had multiple paralogs of Apaf-1, and different paralogs were kept in the different lineages (62). Because of the unclear early bilaterian evolutionary history of the type 1 paracaspases, only deuterostome paracaspases, which are clear orthologs of the vertebrate *Pcasp3* (13), were currently classified and named as *Pcasp3*. Until the invertebrate paracaspases can be more accurately classified and numbered, the three cnidarian type 1 paracaspase paralogs were annotated “A” to “C” (Figures 1–5; Figures S1 and S4 in Supplementary Material) in order to avoid possible future name space conflicts.

### Recent Evolution of MALT1-Like Activities in Deuterostome Type 1 Paracaspases

MALT1-like protease and NF-κB-inducing scaffold activities in type 1 paracaspases were shown to be present as far back as the last common ancestor of the three jawed vertebrate paracaspase paralogs (13). All type 1 paracaspases have a conserved domain structure ever since Cnidaria (Figure 1A), which indicates ancient conserved functions. To further determine the evolutionary origins of these activities, we functionally analyzed the type 1 paracaspase from lamprey (*Petromyzon marinus*) (PmPCASP), which represents the paracaspase function in the last common vertebrate ancestor prior to the dramatic expansion of CBM complex-associated proteins that occurred in jawed vertebrates (Figure 1; Figure S2 in Supplementary Material). For the investigation of conserved substrate specificity of paracaspases within the deuterostomes, we also analyzed the paracaspase SkPCASP from the hemichordate acorn worm (*Saccoglossus kowaleski*). *MALT1*-deficient HEK293T cells were transiently transfected with constructs expressing paracaspases fused to the *ETV6* HLH domain, which is known to induce oligomerization and artificial activation (26), and analyzed for their ability to activate NF-κB-dependent luciferase reporter gene expression (reflecting paracaspase scaffold function) and CYLD cleavage (reflecting paracaspase protease substrate specificity). CYLD is chosen as a model substrate for the evaluation of MALT1-like paracaspase protease activity and specificity since it is a large protein with a single MALT1 cleavage site (R324) and many potential aspecific cleavage sites which are never cleaved by MALT1. CYLD also represents one of the oldest paracaspase substrates (13, 63). The currently most distantly related vertebrate paracaspase with conserved activity, zebrafish (*Danio rerio*) PCASP3 (DrPCASP3) (13) was used as positive control. Only the activated human MALT1 and zebrafish PCASP3 induced NF-κB-dependent luciferase reporter gene expression (Figure 2A), while the lamprey and hemichordate homologs failed to do so. Moreover, the latter two did not show conserved MALT1-like protease substrate specificity either, as determined by CYLD cleavage (Figure 2B). The very poor expression of SkPCASP, most likely related to its AT-rich nucleotide sequence (64), may hamper the detection of a weak protease activity but scaffold activity should still be visible due to the sensitive luciferase readout. These results do, however, indicate that the NF-κB-inducing MALT1-like scaffold activity and the MALT1-like protease substrate specificity evolved relatively recently, after the last common vertebrate ancestor but before the divergence of the three paracaspase paralogs in the last common jawed vertebrate ancestor (Figures S1B,C in Supplementary Material). The conservation of scaffold activity for TRAF6-mediated NF-κB activation is hard to predict by sequence analysis alone: the isoform-specific Ig2 TRAF6-binding motif (TDEAVECTE) and the C-terminal TRAF6-binding site (PVETTD) in MALT1 (22) are PCASP1-specific, but we know that jawed vertebrate PCASP2 and PCASP3 paralogs still are as efficient in NF-κB induction (13). One TRAF6-binding site (TPEETG) located in the Ig3 domain of human MALT1 is found in all three jawed vertebrate paralogs, but is absent in more distant homologs (Figure 3) and could represent the critical evolutionary event for NF-κB activating MALT1-like scaffold activity.

### Functional Conservation or Convergent Evolution of MALT1-Like Activities in Distant Type 1 Paracaspases Reveals That MALT1 Scaffold and Proteolytic Activities Can Exist Independently

Based on BLASTp and subsequent phylogenetic analyses, the mollusk paracaspases were identified as the non-deuterostome homologs most closely resembling vertebrate type 1 paracaspases (13). The pacific sea oyster (*Crassostrea gigas*) was selected as a model and cDNA source (66) for the mollusks. Conversely, the most distantly related species where type 1 paracaspases and *Bcl10* could be found are Cnidaria (13). The cnidarian model organism starlet sea anemone [*Nematostella vectensis* (NvN)] (67) was used as a cDNA source for as distantly related homologous proteins as possible. Neither mollusk (CgPCASP) nor sea anemone type 1 paracaspases (NvPCASP-t1A, NvPCASP-t1B) were able to induce NF-κB in a human cellular background (Figure 2C). We therefore investigated whether downstream signaling components are functionally conserved. The TRAF family of E3 ubiquitin ligases diverged before the cnidarian/bilaterian last common ancestor (68). To investigate whether the type 1 paracaspase–TRAF interaction has undergone lineage-specific divergence, we cloned the Nvn homologs of *Traf2* and *Traf6* and co-expressed them with the two Nvn type 1 paracaspase paralogs fused to the activating *ETV6* HLH domain in HEK293T cells for testing in an NF-κB luciferase reporter assay (Figure 4). NF-κB activation induced by the expression of cnidarian TRAF2 and TRAF6 homologs was equal in the absence or presence of paracaspases, further illustrating the lack of an NF-κB-activating scaffold function in the case of cnidarian paracaspases. Interestingly, the evaluation of MALT1-like protease activity and substrate specificity using human CYLD as a substrate revealed that mollusk paracaspase (CgPCASP) specifically cleaves human CYLD at R324, just like vertebrate paracaspases (Figure 2D), indicating either MALT1-like protease substrate specificity already in the last common bilaterian ancestor (Figure S1B in Supplementary Material), or a convergent evolution of MALT1-like proteolytic activity. On the other hand, the “A” and “B” type 1 paracaspase paralogs from sea anemone (NvPCASP-t1A, NvPCASP-t1B) could not cleave CYLD at all, indicating that paracaspase substrate specificity is not conserved in the cnidarians despite being an organism with a *Bcl10* homolog. In conclusion, our data show the presence of MALT1-like catalytic protease activity in organisms that predate the divergence of deuterostomian and protostomian bilaterians and reveal that the MALT1-like catalytic activity can exist independently of the MALT1-like scaffold function leading to NF-κB activation.

### Functional Conservation of Bcl10-Induced MALT1 Activity Indicates Complex Molecular Interactions

Bcl10 overexpression is known to activate MALT1 activity in mammals. To further investigate the functional conservation of Bcl10/paracaspase coevolution, we transfected *H. sapiens, D. rerio, C. gigas*, and Nvn Bcl10 in MALT1-deficient HEK293T cells with or without reconstitution with human MALT1. Strikingly, not only *D. rerio* Bcl10 (DrBcl10) but also the Nvn Bcl10 (NvBcl10) could induce human MALT1-mediated NF-κB activation (Figure 5A). This result is highly unexpected, since a critical MALT1 Ig domain interaction sequence (residues 107–119 in human Bcl10) that has been identified downstream of the CARD domain in human Bcl10 (61) can only be found in vertebrates. Importantly, the critical 107–119 residues in human Bcl10 are not conserved in *D. rerio*, demonstrating that alternative additional C-terminal paracaspase binding domains in Bcl10 exist. In contrast to human and *D. rerio* Bcl10, Nvn Bcl10 does not appear to be cleaved by human MALT1 (Figure 5A). The observation that cnidarian Bcl10 can activate human MALT1 indicates a highly conserved interaction surface between the two proteins. A conserved Bcl10–paracaspase interaction was confirmed with yeast-2-hybrid analysis, where the Nvn type 1 paracaspase “B” paralog readily interacted with both human and Nvn Bcl10 (Figure 5C). By contrast, the Nvn type 1 paracaspase “A” paralog did not show any interaction with Bcl10. Interestingly, this difference in Bcl10 interaction is reflected by the phylogenetic analysis of the N-terminal sequence of type 1 paracaspases, where the cnidarian “B” paralog clusters closer to type 1 paracaspases from vertebrates and Bcl10-containing invertebrate bilaterian species (Figure S1C in Supplementary Material). In contrast to the functional interaction revealed in the NF-κB luciferase assays (Figures 5A,B), no physical interaction could be established between Nvn Bcl10 and human MALT1 by yeast-2-hybrid (Figure 5C) or by co-immunoprecipitation (data not shown). *C. gigas* Bcl10 failed to induce any NF-κB reporter activity (Figure 5A). In general, samples expressing CgBcl10 show less expression of all transfected components (e.g., MALT1 in Figures 5A,B). This could be due to cell death or other counter-selection in highly expressing cells, which might indicate that CgBcl10 engages in detrimental off-target protein–protein interactions in the human cell background. The invertebrate Bcl10 homologs only show a clear alignment of the CARD domain up until residue 102 of human Bcl10. This N-terminal region has been shown to be required but insufficient for NF-κB induction (61). In addition to the 102 conserved N-terminal residues, all Bcl10 homologs show a proline-rich motif close to the C-terminus. The functional importance of this proline-rich region is, however, unknown, since it is dispensable for MALT1 activation. For further proof that the Bcl10 CARD domain is functionally conserved, we generated hybrid Bcl10 clones where residues 1–102 in human Bcl10 are replaced by the corresponding residues from NvBcl10 or CgBcl10. Hybrid NvBcl10 (NvBcl10 CARD, HsBcl10 C-terminal sequence) showed the same relative level of activation as the wild-type NvBcl10 and hybrid CgBcl10 remained inactive, indicating that the suboptimal induction is due to the CARD domain and not due to lacking protein–protein interaction sequences at the C-terminal part of Bcl10 (Figures 5A,B). Although suboptimal Bcl10 activity (2.5- vs 10-fold MALT1-dependent NF-κB activation), we have demonstrated that the CARD domain, which is critical for paracaspase interaction, is conserved in Nvn. Functional conservation is not always directly related to primary sequence conservation. The very low sequence identity in the corresponding CARD sequence from Nvn (35% identity) is less than the closer related *C. gigas* Bcl10 CARD (40% identity), which lacks the ability to activate human MALT1. Both distant Bcl10 homologs have acidic residues that correspond to E84, a glutamine corresponding to Q92 and hydrophobic residues corresponding to residues L95, I96, and I99 in human Bcl10, which all are critical for MALT1 interaction (61). On the other hand, only NvBcl10 has an acidic residue that corresponds to the critical D80 residue in human Bcl10. From these experiments, we can conclude that the Bcl10/paracaspase interaction is likely to be ancient and that the critical Bcl10 CARD/paracaspase interaction is highly conserved. We also demonstrate that the non-CARD component of Bcl10 required for MALT1 activation is not dependent on a single highly conserved MALT1-binding peptide sequence downstream of the CARD domain.

### A Conserved Intramolecular Autoinhibition Mechanism in Vertebrate CARD–CC Proteins

**Figure 6:**
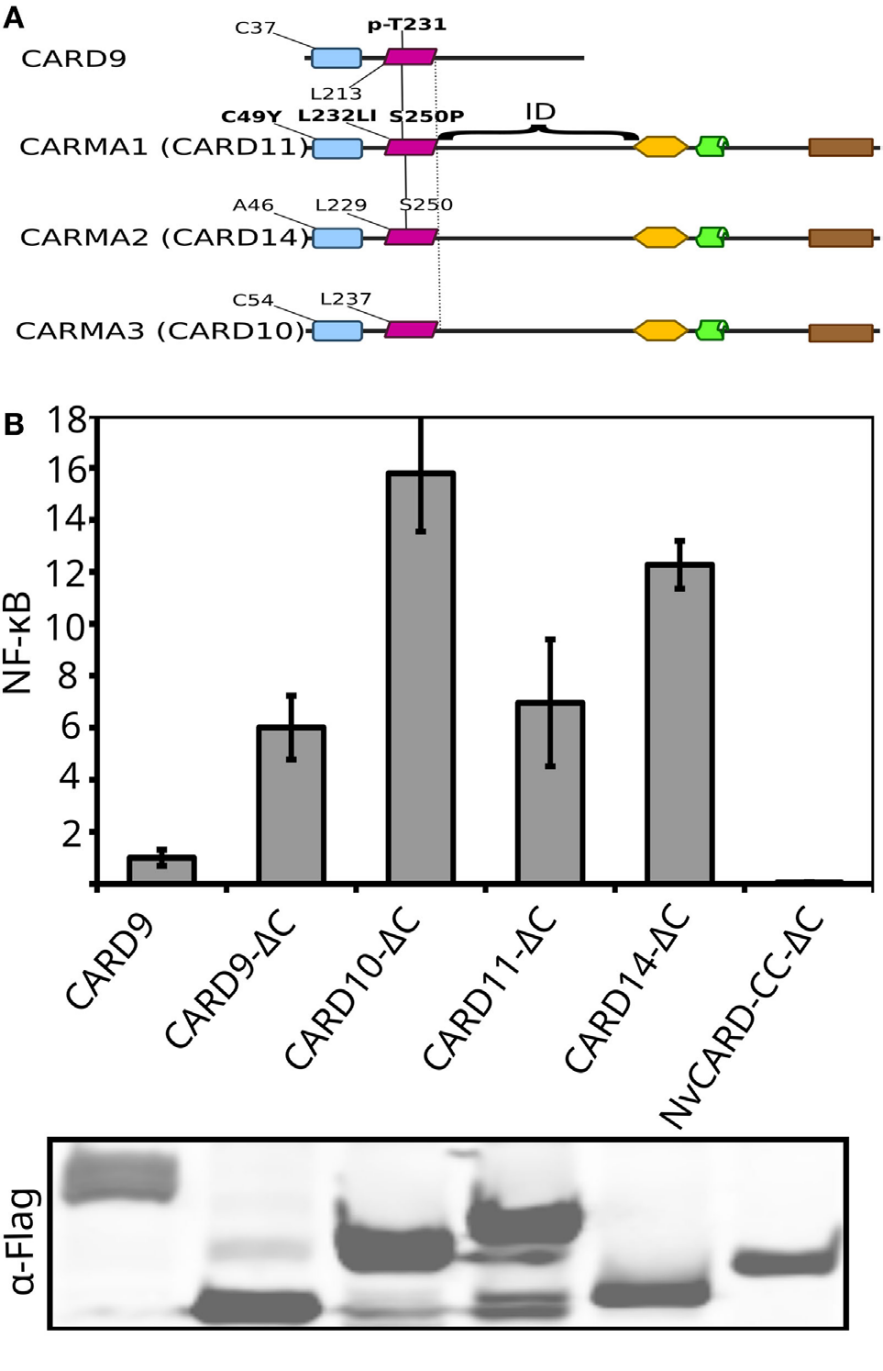
Activation by C-terminal deletions of the CARD–Coiled Coil (CC) protein family members. **(A)** Structural features of the four mammalian CARD–CC family proteins, activating mutations/modifications in conserved residues, are highlighted in bold text. C-terminal deletion mutants in **(B)** consist of the CARD (blue) and CC (purple) domains, to the left of the dashed line. **(B)** Mutants of CARD–CC family members with C-terminal deletions were expressed in human HEK293T cells together with an NF-κB-dependent luciferase reporter gene and a constitutively expressed β-galactosidase reporter gene plasmids. Luciferase values were normalized against β-galactosidase and expressed as fold induction compared to full-length (with intramolecular repression) CARD-9.

Bcl10 has been shown to be functionally conserved as far back as *D. rerio*, as is the upstream interaction with CARD–CC proteins (69, 70). We have now shown that Bcl10 and MALT1-like activities from type 1 paracaspases are considerably older (Figures 2 and 5), most likely preceding the Cambrian explosion (30). We were, however, unable to detect an interaction between full-length CARD-9 or Nvn CARD–CC and the Bcl10 clones in a yeast-2-hybrid assay (data not shown), which could be due to intramolecular CARD–CC autoinhibition. In line with this, both wild-type CARD-9 and the Nvn CARD–CC show very low MALT1-dependent activity (~2-fold, Figure 5D) upon overexpression in HEK293T cells, which most probably is due to an intramolecular inhibition of the CARD domain that prevents Bcl10 binding (71). In CARD-11, the linker sequence between the CC and PDZ domain acts as an intramolecular inhibitory domain (70). We hypothesized that the undefined sequence downstream of the CC domain in CARD-9 and the ancestral CARD–CC proteins could fill a similar autoinhibitory function. We therefore generated C-terminal deletion (ΔC) constructs where the sequences downstream of the CC domain of mouse or human CARD-9, -10, -11, -14, and Nvn (NvCARD–CC-ΔC) were removed. The ΔC constructs were co-expressed with human MALT1 in MALT1-deficient HEK293T cells to test their ability to induce MALT1-dependent NF-κB activation. In contrast to CARD-9 and the other vertebrate CARD–CC proteins, the C-terminal deletion was not able to activate NvCARD–CC (Figures 5D and 6), indicating that the MALT1-dependent NF-κB activation downstream of this protein is due to an alternative mechanism. Taken together, we can conclude that the core CBM complex components are evolutionary linked but that functional evaluation of conservation is problematic due to lineage-specific divergence.

### CBM Complex Components of the Starlet Sea Anemone (Nvn) Show an Overlapping Expression Pattern

**Figure 7:**
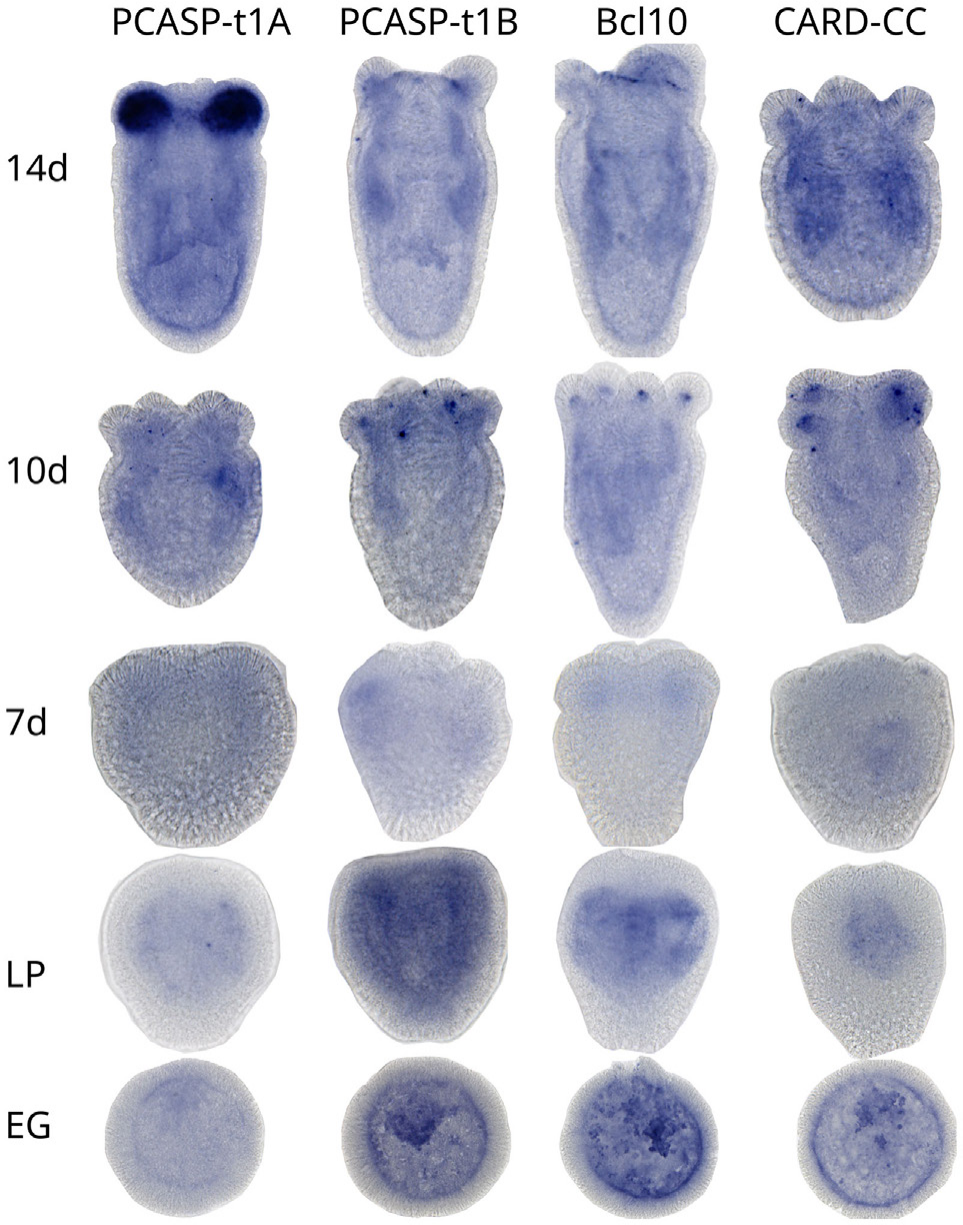
The CBM complex components show overlapping expression in *Nematostella vectensis* (Nvn). Developing Nvn embryos were stained for the indicated genes *via in situ* hybridization at 11 different developmental stages, from the unfertilized egg stage to 14 days post fertilization. Representative pictures from 14 days (14d), 10 days (10d), 7 days (7d), late planula (LP), and early gastrula (EG) stages for all 4 CBM complex genes analyzed are shown.

Since the Nvn type 1 paracaspase paralog “B” was found to interact with Bcl10 of both Nvn and human (Figure 5C) and since Nvn CARD–CC showed a MALT1-dependent signaling activity (Figure 5D), it is likely that these three components together form a CBM signaling complex. Investigation of the expression patterns of the two type 1 paracaspase paralogs, Bcl10 and CARD–CC, during Nvn embryo development (11 stages, from unfertilized egg to the juvenile stage at 14 days post fertilization) using *in situ* hybridization (72) revealed overlapping temporal and spatial expression of the proposed CBM complex components (Figure 7). All four genes showed expression from late planula and onward in a pattern indicating neuronal expression. In addition, all four genes also showed a high expression in the tentacles at the later stages. Although we cannot show the formation of a CBM complex at the protein level in sea anemone, the overlapping temporal and spatial mRNA expression pattern of the proposed CBM complex components is indicative for the existence of such a complex.

### CBM and NF-κB-Independent Functions of Type 1 Paracaspases

**Figure 8:**
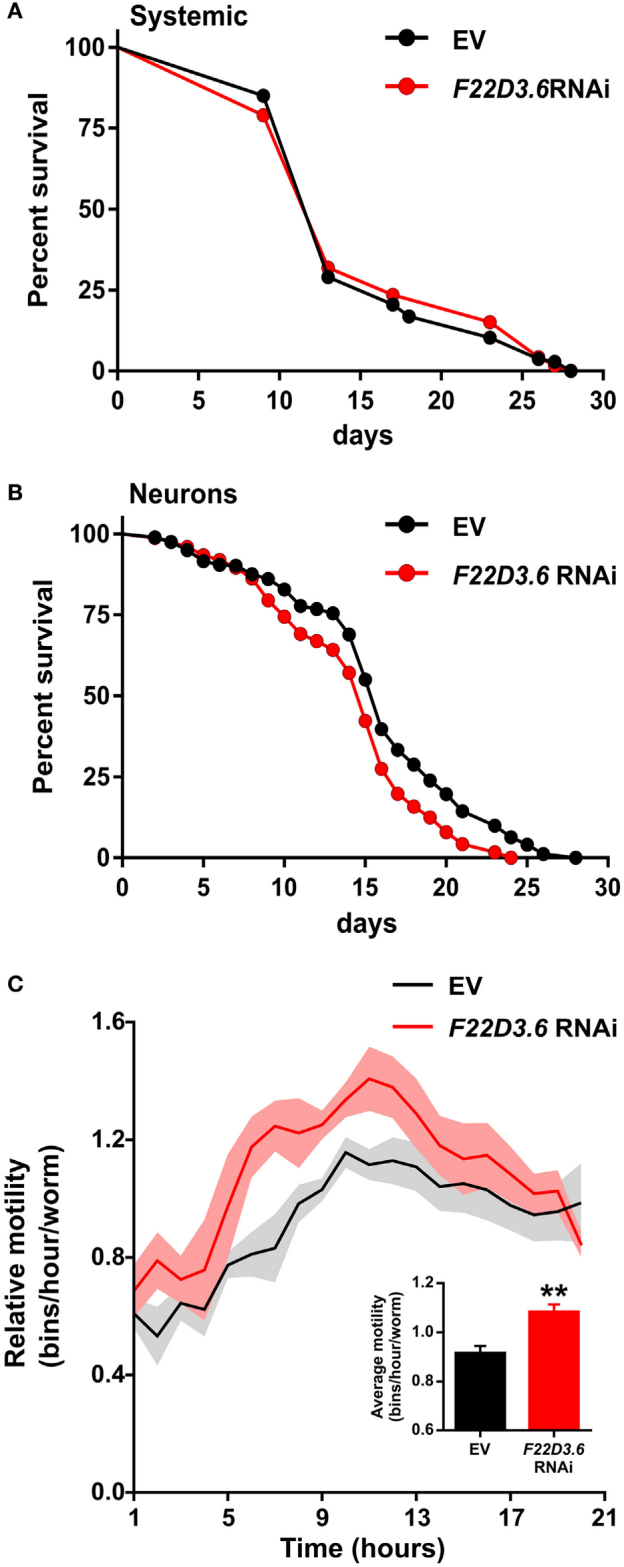
CBM and NF-κB-independent functions of the type 1 paracaspase *malt1* in *Caenorhabditis elegans*. **(A)** Wild-type (N2 strain) *C. elegans* worms were fed *E. coli* expressing RNAi targeting *malt1* (*N* = 119) and regularly monitored for viability compared to worms fed control RNAi (*N* = 107). No difference in life span could be detected (*p* = 0.8194). In these wild-type worms, RNAi is typically targeting every cell except neurons. Silencing in the wild-type N2 strain resulted in approximately 60% reduction of total *malt1* expression levels, indicating efficient silencing in the non-neuronal cells. **(B)** Neuronal RNAi importer transgenic (TU3311 strain; *unc-119p:sid-1* transgenic) *C. elegans* worms were fed *E. coli* expressing RNAi targeting *malt1* (*N* = 399) and regularly monitored for viability compared to worms fed control RNAi (*N* = 364). A reproducible and significant (*p* = 1.4 × 10^−7^) drop in survival was seen upon silencing *malt1* in the neuronal system. **(C)** Neuronal RNAi importer transgenic (TU3311 strain; *unc-119p:sid-1* transgenic) *C. elegans* worms were fed *E. coli* expressing RNAi targeting *malt1* and monitored for motility, using the Wmicrotracker device, compared to worms fed control RNAi. Panels **(A–C)** represent pooled results of three biological replicates carried out at 20°C.

Our functional analyses of non-jawed vertebrate deuterostome and other invertebrate type 1 paracaspases indicate that the remarkable domain conservation in this protein family cannot be explained by currently known mechanisms. The nematode model organism *C. elegans* is a promising system to specifically investigate unconventional functions of type 1 paracaspases because it lacks CARD–CC, Bcl10, and NF-κB. Despite the lack of upstream and downstream proteins in the known paracaspase-dependent signaling pathway, the WormBase phenotype database indicates an important role for the *C. elegans* type 1 paracaspase (*F22D3.6* or *malt1*) with a “lethal or sterile” mutant phenotype (*tm289* vs *tm321*) in nematodes (73). To investigate this further, we analyzed the life span of *C. elegans* in which the paracaspase was knocked down by RNAi. We silenced the *C. elegans* paracaspase gene systemically in the wild-type strain N2 (all tissues except the neurons) and in a strain hypersensitive to neuronal RNAi (TU3311). While systemic knockdown of *malt1* showed no effect on life span (Figure 8A), *malt1* silencing in the neurons caused a slight but significant reduction of life span (*p* < 0.05 in all three replicates, Figure 8B), hinting at a vital role of this paracaspase homolog in the *C. elegans* neurons. Remarkably, paracaspase knockdown in the neuronal RNAi hypersensitive strain also caused an increased motility (Figure 8C). These data indicate the existence of a previously unknown CBM- and NF-κB-independent function of paracaspases in *C. elegans* and possibly other organisms.

## Discussion

### Extensive CBM Complex-Associated Evolutionary Events in Jawed Vertebrates

The presence of three CARMA paralogs (CARD-10, -11, and -14), the split between Syk and Zap70, the evolution of MALT1-like activities, and the divergence of three type 1 paracaspase paralogs in the vertebrate lineage all seem to have arisen in the last common ancestor of jawed vertebrates (Figure S2 in Supplementary Material), which coincides with the evolution of the vertebrate adaptive immune system (74). Interestingly, CARD-11 shows a conserved and non-redundant function in B cell antigen receptor signaling at least as far back as the last common ancestor of mammals and dinosaurs (75). Since all jawed vertebrates have all three CARMA paralogs, a likely evolutionary scenario for the CARMA proteins is that early in the jawed vertebrate evolution, a CARD-9-like CARD–CC got fused with a ZO-1/Dlg5-like MAGUK protein upstream of the PDZ domain (76). Lampreys are in this respect more similar to invertebrates and only have a single ancestral CARD–CC (Figure 1A) and a single type 1 paracaspase, a PCASP3 ortholog which is related to the parent of the PCASP3 and PCASP(1/2) branches in jawed vertebrates (Figure 1A; Figure S1C in Supplementary Material). At this moment, we cannot determine the sequence of evolutionary events between the last common vertebrate ancestor and the last common jawed vertebrate ancestor leading to the increased complexity of the CBM-associated proteins. For example, we do not know if either the evolution of the CARMA paralogs, or the NF-κB-inducing scaffold function of paracaspases, or the expansion of the paracaspases, or the Syk/Zap70 duplication was the primary cause for the CBM-associated expansion in jawed vertebrates.

### Structure–Function Information From Ancient CBM Complex Molecular Interactions

There are already very good structure–function studies done on both the human Bcl10/MALT1 interaction (61) and the human CARD11/Bcl10 interaction (77). Functional characterization of evolutionary distant homologs does, however, provide complementary insights (78, 79). We could, for example, by comparative analysis between CARD–CC proteins generate activated C-terminal deletion mutants in all four jawed vertebrate CARD–CC family members (Figure 6). While in traditional structure–function analyses, single residues predicted to be important are mutated in order to disrupt activity, our identification of functionally conserved distant Bcl10 homologs meant that we could simultaneously “mutate” up to 65% of all residues in the critical interaction domain without completely disrupting activity. To get a better understanding of early Bcl10 structure–function evolution for interaction with CARD–CC and type 1 paracaspases, more sequences from invertebrate genomes are needed (80, 81).

### Identification of CBM Complex Regulation and Function Through Comparative Biology

Investigating early-diverging biological model systems for biological function, protein–protein interactions and signal transduction mechanisms can highlight the original and most conserved functions in a native context. It is worth noting that a MALT1 homolog was recently found to be one of the most significantly upregulated genes in the coral *Orbicella faveolata* after natural bleaching stress, an environmental stress-triggered dysregulated host immune response that leads to dysbiosis (82). By re-analysis of the RNAseq sequences, we could identify the upregulated *O. faveolata* MALT1 homolog at the base of the cnidarian “B” paralog branch (Figure S4 in Supplementary Material). The other three *O. faveolata* type 1 paracaspase paralogs did not respond to the natural bleaching stress. The paralog-specific regulation indicates that the different paralogs might serve unique and specialized functions in these early-diverging organisms. This also indicates that cnidarian paracaspases can still be involved in immune-related responses despite lacking MALT1-like protease- and scaffold functions in our models. The expression patterns of Nvn Bcl10 and CARD–CC are intriguing and might indicate an ancient conserved developmental role, since it has been shown that Bcl10 and CARD-10 are involved in neural tube closure during mouse embryo development (83, 84). There are no neurodevelopmental defects in MALT1-deficient mice (3), but the expression profiles of MALT1 (EMAGE: 12681) during mouse development are strongly neuronal (85). In case there is a functional involvement of CBM complex components in the cnidarian nervous system, the reported interaction of the CBM complex with Notch1 would be a likely conserved mechanism (47, 86). The *C. elegans* phenotype also highlights an important neuronal role for CBM-independent type 1 paracaspase functions and indicates that further investigations on the role and function of *malt1* in *C. elegans* could be highly interesting. It is possible that our RNAi knockdown approach results in a less severe phenotype than the *tm289* knockout, but this mutant also disrupts another gene (*F22D3.1*). Future *C. elegans* analyses should thus focus on clean *malt1* knockout and protease-dead catalytic cysteine knockin mutant worms. Ultimately, identifying the CBM-independent type 1 paracaspase activation mechanism and function in organisms like *C. elegans* could potentially also lead to the discovery of a novel fifth (Bcl10-independent) MALT1 activation mechanism, apart from the four (CARD-9, -10, -11, and -14) currently known CBM complexes in humans.

## Materials and Methods

### Sequences of Homologs of Type 1 Paracaspases, Bcl10 and CARD–CC

Protein sequences of type 1 paracaspase, Bcl10 and CARMA/CARD-9 homologs, were retrieved from NCBI,^1^ Ensembl,^2^ JGI,^3^ OIST marine genomics^4^ (87–89), ReefGenomics^5^ (90), and ICMB^6^ (91, 92), using BLASTp (93). Phylogenetic distribution of Bcl10, CARD–CC, and type 1 paracaspases was also confirmed with the HMMER software package^7^ (94), using the highly conserved CARD and death domain sequences. All full-length sequences used in the analyses can be found in supplemental materials.

### Sequence Alignment and Phylogenetic Analysis

Sequence alignments were performed on the full protein sequence, using the different alignment algorithms Clustal Omega^8^ (95), MUSCLE^9^ (96). Phylogenetic analyses were performed with PhyML^10^ (97) and MrBayes^11^ (98) methods. N-terminal paracaspase sequences were trimmed out from a MUSCLE multiple sequence alignment using Jalview (99). Trimmed N-terminal paracaspase sequences less than 190 residues long were excluded from the phylogenetic analysis. Both alignments and phylogenetic analyses were performed using UGENE^12^ (100) on Arch^13^ Linux (101). For the figures, one of the most representative trees (alignment and phylogenetic analysis) was selected. The final trees were re-drawn to unrooted radial cladograms using dendroscope^14^ (102), and branches were colored using Inkscape.^15^

### RNA Isolation and cDNA Synthesis

RNA of Nvn from various developmental stages was isolated with TRIzol (Thermo Fisher) and pooled. 1.5 µg of total RNA was subjected to 5′-RACE with a GeneRacer kit (Invitrogen) according to the manufacturer’s protocol. RNA was treated with calf intestinal phosphatase to remove the 5′ phosphates from truncated RNAs and non-mRNAs. After dephosphorylation of the RNA with tobacco acid pyrophosphatase, lyophilized GeneRacer RNA Oligo (provided in the kit) was added to the 5′ end of the RNA with RNA ligase. The ligated RNA was reverse transcribed to cDNA using superscript III with random primers and used as templates for PCR amplifications.

### DNA Constructs

Plasmids of the cloned genes were deposited in the BCCM/GeneCorner plasmid collection along with detailed descriptions of cloning strategy and plasmid sequence.^16^ The starlet sea anemone (Nvn) type 1 paracaspase paralog “A” (LMBP: 9589) and zebrafish PCASP3 (LMBP: 9573) were cloned previously. Lamprey (PmPCASP; LMBP: 10451) and the hemichordate acorn worm (SkPCASP; LMBP: 10452) were generated synthetically by Gen9 (www.gen9bio.com). PmPCASP (LMBP: 10453), SkPCASP (LMBP: 10454 & 10455), the Nvn type 1 paracaspase paralogs “A” (LMBP: 9636) and “B” (LMBP: 9825), and pacific oyster (CgPCASP, LMBP: 9826 & 10456) paracaspase were cloned behind the human ETV6 HLH domain for dimerization-induced activation as described previously. Human (LMBP: 9637), zebrafish (LMBP: 9665), pacific oyster (LMBP: 9666), and Nvn (LMBP: 9822) Bcl10 were cloned in the pCAGGS vector with an N-terminal E-tag. For functional characterization of the Bcl10 CARD domain, hybrid Bcl10 clones where human residues 1–102 were replaced with the corresponding sequence from pacific oyster (CgN, LMBP: 10190) or Nvn (LMBP: 10191) were cloned. Also, minimal active Δ119–223 clones of human Bcl10 (LMBP: 10269), pacific oyster (CgN, LMBP: 10263), and Nvn (LMBP: 10264) hybrid Bcl10 were cloned for functional tests. Nvn CYLD (LMBP: 9900) was also cloned in pCAGGS with an N-terminal E-tag. The Nvn homologs of CARD–CC (LMBP: 9854) TRAF6 (LMBP: 9855) and TRAF2 (LMBP: 9856) were cloned in a pCDNA3 vector with N-terminal Flag tag. For further specific cloning purposes, human CARD-9 (LMBP: 9877), Bcl10 (LMBP: 9872), MALT1 (LMBP: 9104, 9105) and Nvn CARD–CC (LMBP: 9873), Bcl10 (LMBP: 9874), PCASP-t1A (LMBP: 9875), and PCASP-t1B (LMBP: 9876) were cloned into gateway-compatible pENTR vectors. For Y2H analysis: Human MALT1 (LMBP: 9880, 9899), Bcl10 (LMBP: 9879, 9885), CARD-9 (LMBP: 9878, 9884), and Nematostella PCASP-t1A (LMBP: 9898) PCASP-t1B (LMBP: 9883, 9888), Bcl10 (LMBP: 9882, 9887), and CARD–CC (LMBP: 9881, 9886) were cloned into the pdGADT7 or pdGBKT7 vectors by Gateway LR reaction. For Nvn ISH analysis: As RNA probe templates, pDEST12.2 clones of Nvn CARD–CC (LMBP: 9908), Bcl10 (LMBP: 9902), PCASP-t1A (LMBP: 9903), and PCASP-t1B (LMBP: 9904) were generated by Gateway LR reaction. For investigations of the CARD–CC activation mechanism through the release of an inhibitory domain downstream of the CC domain, C-terminal deletions of CARD-9 (LMBP: 10457), CARD-10 (LMBP: 10459), CARD-11 (LMBP: 10458), CARD-14 (LMBP: 10460), and NvCARD–CC (LMBP: 10461) were generated.

### Cell Culture, Transfection, and Expression Analysis

TALEN-generated MALT1-deficient HEK293T cells (clone #36) (13) and WT HEK293T cells were grown under standard conditions (DMEM, 10% FCS, 5% CO_2_, 37°C) and transfected with the calcium phosphate method (103). For evaluation of the conservation of cleavage activity, the HLH-fused paracaspase constructs were co-transfected with plasmids encoding wild-type CYLD (LMBP: 6613) or the uncleavable CYLD-R324A (LMBP: 6645) mutant. Cells transfected for cleavage activity evaluations were lysed directly in Laemmli buffer [0.1% 2-mercaptoethanol, 5 ppm bromophenol blue, 10% glycerol, 2% SDS, 63 mM Tris–HCl (pH 6.8)]. For evaluation of the conservation of paracaspase scaffold activity, the HLH-fused constructs were transfected in an NF-κB luciferase assay. For evaluation of NF-κB induction, candidate-inducing constructs were co-transfected with an NF-κB-dependent luciferase reporter expression plasmid (LMBP: 3249) and an actin promoter-driven β-galactosidase expression plasmid (LMBP: 4341) as transfection control. The cells used for luciferase analysis were washed with PBS and lysed in luciferase lysis buffer (25 mM Tris pH 7.8, 2 mM DTT, 2 mM CDTA, 10% glycerol, 1% Triton X-100). For the colorimetric determination (at 595 nm) of β-galactosidase activity, chlorophenol red-β-d-galactopyranoside (Roche diagnostics) was used as a substrate. Luciferase activity was measured by using beetle luciferin (Promega) as a substrate, and luminescence was measured with the GloMax^®^ 96 Microplate Luminometer (Promega). Luciferase data processing and calculation of 95% confidence intervals (Student’s *t*-distribution) (65) was done in LibreOffice (www.libreoffice.org) Calc. For evaluation of the functional conservation of Bcl10 homologs, the Bcl10 clones were co-transfected with the NF-κB luciferase reporter and β-galactosidase expression plasmids in the MALT1-deficient HEK293T cells with or without reconstitution with human MALT1 (LMBP: 5536). Human CARD-9 (LMBP: 9609) was used as control for evaluations of the functional conservation of CARD–CC proteins. Detection of cleaved CYLD was done with the E10 antibody (Santa Cruz Biotechnology) recognizing the C-terminal 70-kDa cleavage band. Expression of the fused paracaspases was determined with anti-Flag antibody (F-3165, Sigma). Human MALT1 was detected by the EP603Y monoclonal rabbit antibody (Abcam) and the E-tagged Bcl10 clones with anti-E-tag antibody (ab66152, Abcam). All Western blots were developed on an Odyssey scanner (LI-COR).

### Yeast-2-Hybrid Assay

The Matchmaker Gold Yeast Two-Hybrid System (Clontech) was used with the Y2H Gold yeast strain to investigate protein–protein interactions. A pre-culture was made the day before transformation, by inoculating about 10 colonies of Y2H gold strain in 5-ml YPDA medium and growing it for about 4 h in a 30°C shaking incubator. The pre-culture was transferred to 35-ml YPDA and grown overnight in a 30°C shaking incubator. On the day of transformation, the overnight culture was diluted to an OD_600_ of 0.2 in YPDA (10-ml YPDA/transformation) and grown in a 30°C shaking incubator until an OD_600_ of 0.6–0.8. After a 5-min centrifugation step at 2,100 rpm at 23°C, the yeast pellet was resuspended in 10-ml Milli-Q water and centrifuged again for 5 min. After resuspending the pellet in 1× TE/LiAc, 100 µl of competent cells was mixed with 100 µg denatured salmon sperm DNA, 1 µg bait plasmid, 1 µg prey plasmid, and 600 µl fresh PEG400/LiAc. The yeast–DNA mixtures were incubated in a 30°C shaking incubator for 30 min. The yeast cells were transformed *via* heat shock at 42°C for 15 min. After a 1-min incubation on ice and a 30-s centrifugation step, the pellet was resuspended in 1× TE and plated on a minimal synthetic drop-out medium (SD) lacking leucine and tryptophan (SD/-Leu/-Trp). After 4 days of incubation at 30°C, colonies were picked and incubated overnight in 200-µl SD/-Leu/-Trp medium in a 96-well plate. Transformed yeast cells were grown overnight in a 30°C incubator. Cultures were then stamped on SD/-Leu/-Trp and SD/-Leu/-Trp/-His/-Ade/+ X-α-gal (40 µg/ml 5-bromo-4-chloro-3 indolyl-β-d-galactopyranoside) plates using an iron 96-well stamp and then incubated for 3–7 days at 30°C until blue colonies were visible.

### *In Situ* Expression Analysis in Nvn

SP6 RNA polymerase (Promega) was used to generate labeled RNA probes. Fixed Nvn embryos were transferred into wells and rehydrated with 60% methanol/40% PBS with 0.1% Tween 20 (PBSTw), 40% methanol/60% PBSTw, and four times with 100% PBSTw. The samples were then digested with 10 µg/ml proteinase K (prepared in PBSTw) for 20 min. The reaction was stopped by two washes with 4 mg/ml glycine. The embryos were washed first with 1% triethanolamine (v/v in PBSTw), twice with 1% triethanolamine/3 µl acetic anhydride, and then twice with 1% triethanolamine/6 µl acetic anhydride. After two washes with PBSTw, the embryos were refixed in 3.7% paraformaldehyde (v/v in PBSTw) for 1 h and washed five times with PBSTw. Samples were prehybridized in 50% PBSTw/50% hybridization buffer (Hybe) [50% formamide, 5× SSC, 50 µg/ml heparin, 0.1% Tween 20 (v/v), 1% SDS (v/v) 100 µg/ml SS DNA and DEPC water] for 10 min, 100% Hybe buffer for 10 min, and 100% Hybe buffer overnight at 60°C. Labeled RNA probes were diluted to 0.5 ng/µl Hybe buffer and denatured at 85°C for 10 min. Hybe buffer was removed from the embryos, and for each reaction, 250- to 300-µl working stock probe was added into the *in situ* plate. The sieves with embryos were transferred to the *in situ* plate and sealed to prevent evaporation. The embryos were then incubated at 60°C for 48–72 h. The sieves were transferred to a clean rack filled with fresh preheated Hybe buffer and incubated at 60°C for 10 min. Then, the samples were washed with 100% Hybe buffer and incubated at the hybridization temperature for 40 min. The embryos were washed at the hybridization temperature for 30 min; once with 75% Hybe/25% 2× SSCT [pH 7.0, 0.3 M sodium citrate, 3 M NaCl, and 0.1% (v/v) Tween 20], once with 50% Hybe/50% 2× SSCT, once with 25% Hybe/75% 2× SSCT, once with 2× SSCT, and finally three times with 0.05× SSCT. Prior to the blocking step, the samples were washed three times with 100% PBSTw (each 10 min) at room temperature. To decrease the unspecific background, the samples were blocked in Roche blocking reagent [supplemented with 1% (w/v) 1× maleic acid] for 1 h at room temperature. The embryos were then incubated with antibody solution [Roche anti-digoxigenin-AP (alkaline phosphatase) diluted 1/2,000 in blocking buffer] at 4°C overnight. The sieves were rinsed with blocking buffer and washed 10 times with 100% PBSTw (each 15 min). The embryos were developed in AP substrate solution [5 M NaCl, 1 M MgCl_2_, 1 M Tris pH 9.5, and 0.1% (v/v) Tween 20] at room temperature. Color development was checked every 10 min for 2 h and AP substrate solution was replaced if an extended developing period was required. Once the probe development reached the desired level, the reaction was stopped by washing with 100% PBSTw. Next, the samples were washed with 100% ethanol for 1 h and rinsed several times with 100% PBSTw. Finally, the specimens were washed with 85% glycerol (in PBSTw) at 4°C overnight and embedded to microscope slides using polyvinyl alcohol hardening mounting medium (10981-100ML, Sigma-Aldrich).

### Microscopy

Images were captured with an Axio Scan.Z1 confocal microscope (Zeiss). Images were acquired with a 20× Plan-Apochromat 0.8 NA dry objective, using a Hitachi HV-F202SCL camera.

### RNAi Silencing of Malt1 in *C. elegans* and Phenotypic Analysis

SID-1 is a transmembrane protein, responsible for the passive uptake of dsRNA but this protein is only present in all cells outside the nervous system. Therefore, feeding RNAi is robust in virtually all cells in *C. elegans* except neurons. To enhance neuronal RNAi, worms [TU3311 strain, uIs60 (unc-119p:yfp + unc-119p:sid-1)] were used which express wild-type sid-1 under control of the pan-neuronal promoter unc-119 (104). Synchronized L1 worms were transferred to NGM plates with RNAi-expressing bacteria (*malt1* RNAi sequences: CCTGATCCGGAACAACAAGT and TCCAGCAGATGCTCATCAAC). RNAi efficiency was evaluated by qPCR using the primers TGATGATGAAGAAGGTGTCTCAA and CATCTCAATCGTCTTCTCTGGAT. The control RNAi is the empty vector L4440. To prevent progeny, FUdR (200 µM) was added before worms became adult. Online application for survival analysis was used to perform statistical analysis of the life span assays (105). To test whether RNAi inactivation of *malt1* is accompanied by neurotoxicity, we performed a motility study with the WMicrotracker. This device is a high-throughput tracking system to record the amount of animal movement in a fixed period of time. The animal movement is detected through infrared microbeam light scattering. A 24-well plate, filled with nutrient agar, was used. Six wells were seeded with the control RNAi bacteria and six others were seeded with the RNAi bacteria against *malt1*. Neuronal RNAi-sensitive worms (TU3311 strain) were grown on RNAi bacteria until young adulthood (day 0). Around 100 adult worms were inoculated in each well. The exact number of the worms was counted afterward. Three independent biological replicates were measured over a time period of 20 h. Data acquisition and analysis was performed as described in Ref. (106). The detected signals per hour were divided by the average worm number in each well. The difference in motility was expressed relative to the control.

## Author Contributions

JS designed and performed experiments, cloned genes, integrated information, and wrote the paper. The paper was commented on and improved by IG, PH, AG, BB, FR, and RB. YD and MH provided technical assistance with HEK293T cell experiments. ID, BB, and AB investigated the role of the *C. elegans* paracaspase. LL and IG performed the Y2H experiments and ISH staining on *N. vectensis* embryos, which were prepared by SS in the laboratory of U. Technau. AG acquired images of the stained *N. vectensis* embryos. PH and IG assisted with phylogenetic analyses and illustrations. JP re-analyzed RNAseq data to identify the coral bleaching-associated paracaspase paralog. RB, BB, YS, and FR supervised parts of this work.

## Conflict of Interest Statement

The authors declare that the research was conducted in the absence of any commercial or financial relationships that could be construed as a potential conflict of interest.

## Footnotes

^1^https://www.ncbi.nlm.nih.gov.

^2^http://metazoa.ensembl.org.

^3^http://genome.jgi.doe.gov/.

^4^http://marinegenomics.oist.jp.

^5^http://reefgenomics.org/.

^6^https://imcb.a-star.edu.sg.

^7^https://www.ebi.ac.uk/Tools/hmmer.

^8^http://www.clustal.org/omega/.

^9^http://www.drive5.com/muscle/.

^10^http://atgc_montpellier.fr/phyml/.

^11^http://mrbayes.sourceforge.net/.

^12^http://ugene.net/.

^13^http://www.archlinux.org.

^14^https://dendroscope.org.

^15^https://inkscape.org.

^16^http://www.genecorner.ugent.be/.
