# Supplementary material for: Ancient Origin of the CARD–Coiled Coil/Bcl10/MALT1-Like Paracaspase Signaling Complex Indicates Unknown Critical Functions
Source: Front Immunol. 2018 May 24;9:1136. doi: 10.3389/fimmu.2018.01136 (PMC5978004; doi:10.3389/fimmu.2018.01136)
Supplement: Supplementary file 1 [file Data_Sheet_1.doc]

**Supplemental Data: Sequences**

***Species key:***

Vertebrates: Hs, *Homo sapiens*, Human; Mm, *Mus musculus*, mouse; Gg, *Gallus gallus*, Chicken; Ac, *Anolis carolinensis*, Anole lizard; Pb, *Python bivittatus*, Burmese python; Xt, *Xenopus tropicalis*, African clawed frog; Dr, *Danio rerio*, Zebrafish; Tr, *Takifugu rubripes*, Fugu; Cm, *Callorhinchus milii*, Elephant shark; Pm, *Petromyzon marinus*, Sea lamprey.

Tunicates: Ci, *Ciona intestinalis*, Vase tunicate

Lancelets: Bf, *Branchiostoma floridae*, Florida lancelet.

Hemichordates: Sk, *Saccoglossus kowaleski*, Acorn worm; Pf, *Ptychodera flava*, Hawaiian acorn worm

mollusks: Cg, *Crassiostrea gigantea*, Pacific oyster; Lg, , Limpet, Ob, *Octopus bimaculoides*, Califonia two-spot octopus, Bg, *Biomphalaria glabrata*, freshwater snail; Apc, *Aplysia californica*, Califonia sea hare.

Brachiopods: La, *Lingula anatina*

Annelids: Ct, *Capitella teleta*, polychaete worm

Arthropods: Dp, *Daphnia pulex*, water flea; Am, *Apis mellifera*, Honey bee, Nav, *Nasonia vitripennis*, jewel wasp; Sm, *Stegodyphus mimosarum*, African social velvet spider, Pt, *Parasteatoda tepidariorum*, common house spider; Si, *Solenopsis invicta*, Fire ant, Lp, *Limulus polyphemus*, Horseshoe crab; Zn, *Zootermopsis nevadensis*, termite; Ls, *Lepeophtheirus salmonis*, Salmon louse.

Nematodes: Ce, *Caenorhabditis elegans*; Dv, *Dictyocaulus viviparus*; Hc, *Haemonchus contortus*

Cnidaria: Nv, *Nematostella vectensis*, Starlet sea anemone; Ep, *Exaiptasia pallida*, sea anemone; Hm, *Hydra magnipapillata*, Hydra; Ad, *Acropora digitifera*, Stag horn coral.

***Paracaspase sequences***

>HsMALT1

MSLLGDPLQALPPSAAPTGPLLAPPAGATLNRLREPLLRRLSELLDQAPEGRGWRRLAELAGSRGRLRLSCLDLEQCSLKVLEPEGSPSLCLLKLMGEKGCTVTELSDFLQAMEHTEVLQLLSPPGIKITVNPESKAVLAGQFVKLCCRATGHPFVQYQWFKMNKEIPNGNTSELIFNAVHVKDAGFYVCRVNNNFTFEFSQWSQLDVCDIPESFQRSVDGVSESKLQICVEPTSQKLMPGSTLVLQCVAVGSPIPHYQWFKNELPLTHETKKLYMVPYVDLEHQGTYWCHVYNDRDSQDSKKVEIIIDELNNLGHPDNKEQTTDQPLAKDKVALLIGNMNYREHPKLKAPLVDVYELTNLLRQLDFKVVSLLDLTEYEMRNAVDEFLLLLDKGVYGLLYYAGHGYENFGNSFMVPVDAPNPYRSENCLCVQNILKLMQEKETGLNVFLLDMCRKRNDYDDTIPILDALKVTANIVFGYATCQGAEAFEIQHSGLANGIFMKFLKDRLLEDKKITVLLDEVAEDMGKCHLTKGKQALEIRSSLSEKRALTDPIQGTEYSAESLVRNLQWAKAHELPESMCLKFDCGVQIQLGFAAEFSNVMIIYTSIVYKPPEIIMCDAYVTDFPLDLDIDPKDANKGTPEETGSYLVSKDLPKHCLYTRLSSLQKLKEHLVFTVCLSYQYSGLEDTVEDKQEVNVGKPLIAKLDMHRGLGRKTCFQTCLMSNGPYQSSAATSGGAGHYHSLQDPFHGVYHSHPGNPSNVTPADSCHCSRTPDAFISSFAHHASCHFSRSNVPVETTDEIPFSFSDRLRISEK

>MmMALT1

MSLWGQPLQASPPLAVRQPPTASSGPSTSPPAGATLNRLPEPLLRRLSESLDRAPEGRGWRQLAELAGSRGRLRLSGLDLEQCSLKVLEPEGSPSLCLLKLMGEKGCTVTELSDFLQALEHTEVLPLLNPPGLKITVNPESKAVLAGQFVKLCCRATGHPFVQYQWFKMNKEIPYGNSSELVFNTVHVKDAGFYVCRVNNSSTFEFSQWSQLDVCDVAEVTDSFQGSMDGISESRLQICVEPRSQRLVPGSMLLLQCVAIGSPMPHYQWFKDESPLTHETKKHYTVPYVDIEHEGTYWCHVYNDRDSQDSKKAEVTIDELNNLGHPDNKEQTGQPLAKDKVALLIGNMSYWEHPKLKAPLVDVYELTNLLRQLDFKVVSLLDLTEYEMCNAVDEFLLLLDKGVYGLLYYAGHGYENFGNSFMVPVDAPNPYRSENCLCVQNILKLMQEKETGLNVFLLDMCRKRNDYDDTIPILDALKVTANIVFGYATCQGAEAFEIQHSGLANGIFMKFLKDRLLEDKKITVLLDEVAEDMGKCHLTKGRQALEIRSSLSEKRALTDPVQGAPCSAEALVRNLQWAKAHELPESMCLKFQCGVHIQLGFAAEFSNVMIIYTSIVHKPPEIIMCDAYVTDFPLDLDIDPKHANKGTPEETGSYLVSKDLPKHCLYTRLSSLQKLKEHLIFTVCLSYQYSGLEDTVEEKQEVNVGKPLIAKLDMHRGLGRKTCFQACRMPDEPYHSSTSTSAGAGHFHSSQDSFHDVYHSHLGNADSGMPPDRCHCSRTPHTFISNYPPHHYCQFGRSNVPVETTDEMPFSFSDRLMISEN

>GgPCASP1

MSNYTHWYSEVPGPGCPRQMLAVACSAVCSGFGLGRAADTPTSRPAHRLRPPGAEPAVPPRSRAAAPVASFRAGAPPRAGRGWRRRRAGGSGAMSEPPCSPPGSLPLSRLAEPLLRRLSELLDRAAPGKGWRELAQRAGSRGRVRLSPLDLEQCSLKVLEPEGSPSWSLLKLLGDRGCTVVELVEFLQAMEHTEALQCLSYSGIKIVVQPDSQAVLCGQVVKLCCWATGHPFVHYQWFKQKKEVPHGNSPELVLNPVKVNDAGFYICRVNSESSFTFSRWARLEVCDLQDTAHGSLISLPEKKLCICIQPQPQNLTVGDALVLECGAVGNPIPQYQWFRNGFPLANGNKNVYTVSCVDMEHGGTYWCHVFNDKEDQDSKKIEVIIGRKAMAVECTEEELSDLQKTADLQEQFSDRPFATDKVALLIGNMSYWNHPQLKAPMVDVYELTNLLRQLDFKVVSLLDLTESEMRNAVDEFLLLLDRGVYGLLYYAGHGYENYGNSFMVPIDAPNPYRSANCLCVQNILKLMQEKETGLNVFLLDMCRKRNEYDDTILILDALKVTANIVFGYATCQGAEAFEIQQLGLANGIFMKFLKERLLEDKKITVLLDEVAEDMGKCHLTKGKQALEIRSSLSEKRALSDPIQQTISSAESMARNLQWAKAHELPESMYLEFKCGVQIQLGFAAEFSNVMIIYTRIVKKPPEILVCRAYVTDFPLDLDVDPKEANKGTPEETGSYLVSKDLPKHCLYTRLSSLQKLKEHLIFTVCLHYEYPGIEDTMDERKEVNVGKPLIAKLGLHHGFRNNCLQTCCVANNLFHSQVESSPVVTKYYFPSRCQPNSCPGAYHPNCTCPGSTRQLEACSCSGTSRMLASRHEVQNYSPPAEKSNVPIETTDDTVELEFLLSDSLGLSKQQ

>GgPCASP2

MGDWSLPIGSLGEEVVAQLCELLDTASRGWRKLAEVAGAEKRFKCSEEELEMCSLKVLEPLGSPTQCLLQLLAERECTLRYLQGCLHRMGHTQACQVLSSAVHDVIRITVQPESQVVAEGTRVSLTCWASGPPGLTYQWFCGRQEVPGATAPGLMVDTAAPLGLPQWYICRVSCGAAFAFSRWARIQVERSHSPRISQRLLPQHGGAADPAAATAVPPAGGGPAGVECRALGNPPPQYQWFRNRRPVEGARAPQLQVQLVTTAERGTYSCRVFNLFHELWSQEVDVGIGPRLFASGAPWQDGDGDSPEPGSPDQLYATDKVALLVGNMHYVHHKHLRAPMVDVHALSALLRQLDFKVVSLLDLRRDEMQMAVDEFLLLLDKGVYGLLYYAGHGYENFGTSFMVPIDAPGSYTSAHCLCVQHVLQRMQQRRTGLNIFLLDMCRKRNLNDDVIPQVGALEVTANIVFGYATCADAEAYELSQQGELSNGVFVTFLKRWLLEDEKITVLLDKVAEDMGTLELTRGRQALELRSNLSERRALTDPIRSLGQAETSARNLQWAKAHVLPESRHLRFDCGVTVQLGFAAEFSNIMIIYTRILAAPGDVTECVAKLTDLPEELDVDLKCTNRESPEELGSPLVPTWSVTCPSCCLYSRLCGLQRLRQELTFTVCLQYRYRHVADFVEERRAVSVGRPLIAKLNLSPPDPGKLPASGGSGSPLSASPPSSWGNSPEENLSPEVPGSHGV

>GgPCASP3

MQTAIVITEQPVSVSVPVGYSFTLHCRAEAHTSLQYQWFCQCQSVCHQIPGATKQDLPITAQKTKLYTCRINDLYRNVIFSDWVKVEVHPCVARGLLPQLWQGEPIIILNPTEQKVEVGKPLQLQCAAIGVPAPSYQWYRNGNLLEHQKKKKLWITHTKVSDSGTYLCCASNSHGEHWTNAVDIHIGTCCSEKFFATGKLALLVGNNHYQHHPNLMAPVTDVFELSLLLKQLGFQVVSLLDLNKAEMVAAVSRFLQLLEKGVYAIFYYAGHGYEHLGRNYMVPVDAPQLYAPENCISVQRILQKMQQQQTALNLILLDTCRKWYNPECALSQVQPLEPWGNTVYGYATSEDAEAYELQNGEFSSGIFMKYLKKHILQEKKVTHMLEDVLEDLGRDPVVTGKQVMEIKHTLKEARSLTDPICPTGAATERWGCSREPPSETVTFPCGVQAELRFHRLFSNVLSVCAKLQDLPAHLTDAQLMLRQPTEVDDVANLEESHPDQMDSLLTSVYKQEELDCIFQLCGLQKIQMDVVLQLDLHYTQLSTKQRTCESLQKILPKLLLGQFFTQRNYTRPNYDSSPARADPGGALSTGTGRSLPSSSPSKSRSEPEENDERSMSELCSALLRAGPGQEHP

>AcPCASP1

MAGSGPGPSSSSSSSFCSNSLPLQRLPEPLLALLSDSLDRAPPGKGWRELAQVAGSQAGLRLRLSSQELEQCSLQVLEPEGSPSRSLLQLMGERGCTVLELTELLQALKHTEALQYLNPPSIKIVLEPESLTAVSGQTVKLSCWAAGYPLLHYQWFKGDKMVPHGNSPELIFNQVSVDDAGFYICRVNSDFSYGFSQWARLDVSEGCGACHEDLSCLGEPDLTEEQSTERQYAVDKVALLMGNMSYRNHPKLKAPLVDVYELTNLLRQLDFKVVSVLDLTEPEMRNAVNEFLLLLDKGVYGLLYYAGHGYENYGNSFMVPIDAPNPYRAANCLCVQNILRRMQEKQTGLNVFLLDMCRKRNEYDNTILVLDALKVTANIVFGYATCQGAEAFEIQQSGLSNGIFVKFLKERLLEDKKITVLLDGVAEDMGKCHLTKGKQALEIRSSLSEKRALTDPIQQTVTSAEALARNLQWAKAHELPESMCLQFNCGVQIQLGFAAEFSNVMIVYTRIIKKPPEITVCNAYVTDFPLDLDVDPKEANKGTPEETGSYLLSKDLPKHCLYTRICSLQKLKEDLTFTVYLHYEYDGVDEVVDERKVVNIGKPLIAKLRIHQGFGKNSCLQHCCVSSGPCFTQSSDAGPAVYHPSHPNHHPHSYAGFPQPNSAHPEGISHPATCHCDMTPNRLMAWQTTWPYSFSSNQGNMPVETTDEQESDFSGNFRLS

>PbPCASP1

MGERGCTVLELTEFLQFLELTEALQLLNPSIKIVVEPESQAVLSGQMVKLSCWATGYPLLYYQWFKEKKMVPNGNSPELVFNQVTVDDTGLYICRVNSDSSYEFSRWAQLEVCDSQGDSDGNYFELPEKKIRICLHPQSQNLMVGEMLVLQCEAIGIPVPQYQWYKEEFPLTNGDKKVYVISSVNIEHQGTYWCRVYNSQENEESRKVKVTIGGKSIAVECTEEELNDLERPDLEQPTERQFAVDKVALLMGNMSYYNHPKLKAPLVDVYELTNLLRQMDFKVVSLLDLTEPEMRNAVDEFLLLLDKGVYGLLYYAGHGYENYGNSFMVPIDAPNPYRAANCLCVQNILKLMQEKDTGLNVFLLDMCRKRNEYDNTVLILDALKVTANIVFGYATCQGAEAFEIQQSGLANGIFMKFLKERLLEDKKITVLLDGVAEDMGKCHLTKGKQALEIRSSLSEKRALTDPIQQTVTSAEALARNLQWAKAHELPESTCLQFQCGVRIQLGFAAEFSNVMIIYTRIVKKPPEITVCNAYVTDFPLDLDVDPKEANKGTPEETGSYLLSKDLPKHCLYTRISSLQKLKEDLTFTVCLHYEYMGLEDTVD

>XtPCASP1

MSDLHQFNSLNINLLQEPVLQKVCDLLDQKSDKGWRKLAGVIASDSRFKISSQELHQCSLKVMLSEGSPSRSLLKLMGERGCTTKDLTEFLQSIGQSNAIQLLKPPGIKVIMQPEPMAVLAGQMVKLTCVGSGHPFVNYQWFKMDKEVPQGNSSELVFNPVQVQDAGSYTCRVNNGNSYDFSGWAQLDVRESHLRSHAGLPGIPDYRVQICTQPQPQSLMPGERLVLECAAVGKPLPWYQWYRNAVAIKDGTKKQYTVSSVSAEHQGLYYCQVTNGNESELSREVEVVVLGTKKLWTNTAVECTEEELKVLTISETGNEDLFAVDKVALLIGNMSYKHHPQLKAPMVDVYELTNLLRQLDFKVVSLLDLTELEMKSAVNEFLLLLDKGVYGLLYYAGHGYENYGNSFMVPIDAPKPYRHTNCLCVQKILKLMQEKETGLNVFLLDMCRKRNQYDDTILILESLKVTANIVFGYATCQGAEAFEIQYPDLANGIFMKFLKKRLLENKKITVLLDQVAEDMGKCQLTKGMQALEIRSSLSEKRALTDPIRQTACSPEGLARNLQWAKAHELPESMAIEFDCGVKMKLGFAAEFSNVMIIYTRITEKPAEIIHCNASITDFPLDLNVDPKETNKGTPEETGSFLVSKELPKYGSYTRLSSLQTLKENLTFTVCLSYMYQGIEETLDENKRVDVGKPLIAKLDIHSGFKRNLSVQTLHSPCHSPELGLGVAEASHVCSSPRYSQPHFNAYHSNARTAVYPGTGPTDIYSGFSPVSNRSRYFPEPIITYNVPEETTDDNNLDFPQ

>XtPCASP3

MLKDIVIAEQPVCACVPRDFPLTLRCRAQGSSPLYYQWFLHSESFCKEIPGANQPDLHIVTPQTQLYICRVNDQHHHFAFSHWVKVKVLKDITKDYVLPGWEGDPVFITYPTPAHLTSQQPVQFQCCAIGIPAPKYQWYLNGQCLPHRRGRKMQIKAVKPKDCGSYLCCATNAKGERWSEPVELTLEHSPTRQKDIFFAVGKVALIIGNNCYLKHPNLLAPVVDVYELSNFLEKLEFCVISLVDLTHSEMLIAVNKFLQFLDNGVYGLFYYAGHGYERSGRNYMVPIDAPQPYRPENCISVQKILQKMQERRTALNVVLLDTCRKWYNADCALSKVNPLKPFGNTVYGYATCENAEAYEVQDGDSSSGIFMTYLKKHILEEKKVTHMLEDVLEDIGRDPLITGKQVMEIKHTLTESRALTDRICSPQSERLNRKAWDKENELPKQILLFSCGVEVELSFQSVFSNLIHIFARLIQTPSYLTDIRTILYICSDFAELSIPNTCRLGHEDSLLAMGSEGEGADCMLRLPGLQKCQNDIFIKLDLHCTNMGTGVCLQESLEHRIPKPWVARLFSQKDFSLDSAAGSSDDSRSRQRLQSSTNSHTLGHAGGISLGAQSKSSSEPEEHDEGDIFAAK

>DrPCASP1

MSESNESSLNINFLKESVLKRLCESLDKANNKGWRKLGEIVKNDKRFKVSLEDMDMCSLKALKADGSPSRTLLKLIGDQGCIQEELMEFLQIMGHTEALQCLKPPGIQILLQPQSVSVIAGHNLRLSCYAVGASPVQFQWFKKTEEVRSSSSPDLVFSPVQIRDAGFYICRVNCGSTYEFSQWAQVDVLDVPTRYGLVSMGSEVRLRVLIQPQPQKLLTGDLLYLECGAAGRLLPQYQWYRNGAPIKKANKRKFTVKNVLPEHQGRYRCEICCNNERTWSSEVDVVIANPCLSQMKEARIFPQISGAMECSEDDFLSINKDNSFEKPYATDKVALLIGNLAYRNHPQLKAPMVDVYDLTNLLRQLNFKVVSLLDLTESEMRNAVEEFLSLLHKGVYGLLYYAGHGYENFGNSFMVPVDAPNPYRSGNCLCVQSILKLMQEKETGLNVFLLDMCRKRNMHDDAMPNVMLKVTANIVFGYATCQDAEAFELSSSGFTNGVFIKFLKERLLEDEKITVLLDRVAEDMGQFDPTKGKQALEIRSSLSERRSLTDPICHGGYSDATEAHNPLWSKAHELPESKVLDFECGVRIKMDFAAEFSNVLVIYTSIVKKPEEMQSCQAHITDLPLDLDVDPKLMNKETLEETGSFLLSGSLPQHCLYTRLSSLQKLKEELAFTVCLQVQFSSLDDLVQWNMHVNIGKPLTAKLDLYRPTRKNSCQVTCHMPHSPSTSPSHSPGSDRRFHSQEPCCPQPYANPYLNVCEHLHGAQAGYRDFQLNTDHAYQYMPLSPLTNPPSIPIETTDDIDDMQNDFINSLHLYKHP

>DrPCASP2

MEDKNLGLENISEAALSRLGYMLDNAECGWKQLAKAVAEQPQFCYSDREMTDCSLKVLSPHGSPGRYLLALLADRGCTLAFLLQCLKKIEHREAVGFLTMNVMEQIDIIQQPQSQRIAEGSPVVLSCKAVGPVELQYQWFKGKDEMPEGNSPDLVFNRASPAQQGYYICRVSSGDKYIFSNWAHVYLLRSGGASADSSSSYFPSTASGLNITQQPRPQALMEGDTLCLECSAQANPPPQFQWYFNKELMPKCIRNFLKIPCITTADRGIYACRVYNLYHEMWSDQVQVNIGPGSCSESSWEKTAVDNPEANLKQMGDCCATDKVALLIGNMNYLHHRQLRAPMADVHELTNLLRQLDFKVVSLLDLNWQEMHSAVTEFLLLLDRGVYGLLYFAGHGYENYGNSFMVPIDAPASYTFKHCLWVQEVLQRMQERQTGLNVFLLDMCRKRNPNDEGIPQPDPLRVTANIVFGYATCVDAEAFEVNKDDLSNGIFMSFLKKRLMEPEKVTVILDKVAEDMGRCEITRGRQALELRSNLSERRALTDKIQAPACQIAASTRNLQWAIAHVLPESRCLQFECGVTVQLGFAAEFSNIMIIYTQILETPTDIVSCSAQLSDFTEGPEVDLKRSNQESLHEAESFLLTIDDLLPLKQPQLFTRISALQRLKKELSFTVCLHYKYGNLDEELLEEKRITVGKPLVSKLNLHEPRLFHSSSSDQQMSSFLDSSSFSESFRANLPDLKQNFIGSASTSLPNYRVPMESFACFGLKNEPEETLCPEFLESEEPPAIKSLPSASSEELPFKFSNFQNSI

>DrPCASP3

MTDYLKRSWPSTLKSSTIVMRTEVVILQQPLPVCVPPNHEVTLRVQAKGTGALKYQWFDKQQTEVVGGTEPELTVSLQRSGNYVCRVSDLYCNYQFTEWVKVRILDEAGVSNTWRGEPHVVIHPKSQTIKPGETFTLKCTALGKPAPTYQWYRNGRILTEETKETLKVENAGAEVAGSYLCAVSNILEERWSEAAEIEIAPPDQLPTTTLMATDKVALLIGNLNYSHHPGLMAPTMDVHELANLLQQLDFRVVSLLDLTLEEMQAAIDKFLQLLDRGVYAVFYYAGHGYEHAGRNYLVAIDAPRPYRTENCVCVQRVMRSMQERKAQLNVILLDTCRKWYNQKGPPSEIKPLAPLGNTVYGYATSEDAEAFEVQDGGKSTGIFTKYLNKHVLQPERVTRVLEQVSEDLGKDPLVRGRQVVEIRHTLMEPRALTDPVRTSGHTMELRRRDAYWKQANVLPGRRTLRFHHSGVQVELNFSALFSNVLVVFGTVRNTSAMAQDCTLILESEPNLQDIFSASGRSEEMDSLFVSGGQTPDCTLRLCCLQRLQGSVVIRAKLHYTHAQTKERLTESREVDIDRPLISSCKLNQMKTVDRRQDARGHTLDHMSNIRWSQHHRPHHHQQQNHSRIGRPYTRKAENLLQSSAKSSSMSSNEPEENDENDENEFTM

>TrPCASP1

MSDGLERSVKVSLLKEPVVRRLCEVLDQSSNKGWRKLGEIVGNDRRFKVSPDDMEMCSLKVLQPEGSPSRTLLKLMGERGCTTGHLMDYLQTVGNAEALQCLKPPALQILVQPQSVAILSGHNLRLSCYAVGKSPVQYQWFKGKDEVHNSTSPDLMISPVSLRDTGFYICRVNCGDAFEFSQWAQVDVLNTNMPCGQSYQSAGGRLKLVIQPKLQRLQVGENLQLECGAVGRPIPRYQWHRNGVPIPKATKRKLVIPHAMQEQHGRYRCEISSSTERLWTNEVDVVIDDIYAIGGGPSDFLLNSIPEQLYATDKVALLIGNLSYQNHPQLKAPMVDVYELTNLLRQLNFQVVSLLDLTESEMRNAVDEFLLLLHKGVYGLLYYAGHGYENYGNSFMVPVDAPNPYRSANCLCVQSILKLMQEKETGLNVFLLDMCRKRNVHDESTPNIVLRVTANIVFGYATCQDAEAFELSSTGFTNGVFVKFLKKRLLDDEKITVLLDRVAEDMGQFDATKGKQALEIRSSLSERRALTDPILPGNSPNMAHTLSRHWAKAHELPESMCLDFDCGAQIKLGFAAEFSNVLVIYTHIIKKPEDMSLCQAHVTDFSQDVDVDPKEMNRETPEETGVYFLSSSLPQHCLYTRVGALQKLKEELAFTLCLQGTFVSMDDDTVHWTKTINIGKPLIARLDLHRHVRRNSCLQTCLMPHSPSHSPCHSPCHSPCHSPGPEHRLHIIPGPYQVQDYARLSPQHHYLDVYEPLQGAVGGSGESYYENVSQPQYDPAYVSAEKLSSSSSRMSIPIETTDDISELQTVFINSLQLQQQ

>TrPCASP2

MEEKSLVSLGEDIVNKLALMLDRSTCGWRQLASMVSEHPKFRFSENELTSCSLQVLRPTGSPGRAVLAVLADRFCSLNVLLDCLRKIDHQEAVQYLTSSVAEIQISVQPQSQQAAVGSRVSLTCRAFGPPDLNYQWFRGKEEILGEKGTSPELILCPLAPVHQGHYICRINNGIKCIFSQWAHISVIRSADCSSSLLTGSVSGLFITSHPQSQVVSEGDTLFLECNALANPPAQYRWHHNMVPMEEEKSRLLQIPCLTTAHRGQYRCKIFNLYHEAWTDAATITIGPSSITDASWIESSLGQEDTMTISEHKIQHSTNRTPLAKQFYATDKIALLIGNMNYIHHTQLCAPIADVHELTNLLTQMDFKVVSLLDLNWQEMHSAVTEFLLLLDKGVYGLLYFAGHGYENYGNSFMVPIDAPASYTSEHCVCVQSILTRMQEKQTGLNIFLLDMCRKRNPNDDIVVLPGLIKVTANIVFGYATCVDAEAYEVKREDVTNGIFISFLKRRVCEDEKVTVMLDRVAEDMGRFEITRGRQALELRSNLSERRSLTDPIMTPENCSASSSARNLQWAVAHVLPQSLCLQFDCGVKVQLGFAAEFSNVMVIYTRILEKPKEVSSCCAQLTDFPEDVDVHMKKSNQESLLEAGSLLINEENLSSPEGPSLYTRVRSLQRLKNEFTFTTCLHYNYSHMNEEVQERQTVTVGKPLVSKLNLHEPRPSFSASSFICRDSLEFARCSSITEDLISAGSNACPHSAETDKKHQTDADFPYSSIGSNTPVETEASEIVDAPQPLVTGKSLPHNQINDTNYNFSDIYGFHSY

>TrPCASP3

MSRPCRMMIGEVVIVSHPVSMCVPVNHTVTLSVRAEGTGLLQYQWFTNEANVVCEVFGANEADLTVKAKKTRPYVCRVNNHFNKAVFSEWVKLKVLDIDKSGLPSDWQGEPHIAVNPDPQTVQRGTDVTLRCTAFGIPTPHYQWYRNGQELLKKTGDTLQIVGATEEDAGSYLCCISNVLEERWTEAVEVLISTAQQELPSAAFTATDKVALLIGNLNYSNHPKLMAPVVDVHELSNHLQHLGFRVVSLLDLTREEMLASIMHFINLLDKGVYGLFYYAGHGYEHAGRNYLVAIDAPQPYGPENCVCVQRIMLSMQKKHTALSVILLDTCRKWYYQDCLPSCLMPLGPSGNTVYGYATCEDAEAFEVQDGRKSTGIFTKYLNMHILESEKVTHVLEKVSEDLGRDALVTGKQVVEIKHTLKEPRSLADPVRTTGHTRELHLRDICWSQANVLPRKKQMLFLCGVEVQVSFSALYSNVLVVFATLKSICSHIQDCTVTLSSLPAMKDIFSRPGTSLEMDSLLFNPLDNPDCTLRICSLQKLKESLVIKVDLHYTHMTSNQRQTESQQVDIGRPLVASCKLYKRSPLSTIR

>CmPCASP1

ISHLNEPLLNKLSHLLDQSGWRKLAEMASADKRFKISSEELNNCSLKVLTPEGSPTRNFLRLMADRGMTLRDLSGYLQALDHAEAIQLFRSAVSLMIIIQPVPQAVLAGQVVRLRCQAAAHHVVEYRWFKMQVEVPNGNSSDLVLNPVTVNDTGRYICRVNIGMDFNFSQWVRLEVYNFSATYGRPFLLLDNRLQIINQPQSQHRMVGDMLVLECTAIGVPVPHYQWLRNGERVDCTERHYKVARVSPQHQGVYRCRVFNGKEDYWSTEAEVVIGKHMNIRISRQKKTRSIQFAILAVTCTCVPILAATDKVALLIGNMSYHNHPKLKAPMVDVYELTNLLRQLDFKVVSLLDLTEFEMRNAVYEFLLLLDKGVYGLLYYAGHGYENYGNSFMVPINAPNPYRSAHCLCVQSILKLMQEKETGLNVFLLDMCRKRNVYDDTIPNVEALRVTANIVFGYATCQGAEAFEIQHYGLANGIFMKFLKERLLEDEKITVLLDRVAEDMGTCDLTKGKQALEIRSSLSEKRALTDPIRSSGCSVESLARNLQWAKAHELPESKWLDFECGVKIQLGFAAEFSNVMIIYTRIVGKPKEIVMCDAYVTDFPTDLDVDPKKTNRGTPEETGSYLLSKDLPEQCLYTRLSALQKLKERLVFTVCLRYQYQGLSELVEEAKEVDVGKPLIAKLDIHRGVRRNSCLVTCPMPHSPSHS

>CmPCASP2

MGDWNLLLLSVSEEVMQRLGKKLDNPTNGWRRLARMVGIEQRFQCSEKEMDQCSLKVLEPVGSPGRSFLQILADRGCTVWYLLSCLDRMDHREAVGCLLPLVPAAVCITVQPESRDVVVGERVVLYCGAVGASGLSYQWFQGSREVKRSTFTVCMGYGVCMGVCVYGVCMRVSTRDSSRLQFLPHYYFHRPVIAFAHRATRYVCSLRILVHPNSLRLQEGQHMSLQCSATGTPPPQYQWFRNNQSIPDATESSLKVTGVTTADRGVYCCRVYNLYQQVLSREAHVEIGKVSPEIDWQPPADCTPWFLSPATDKVALLMGNMNYQHHKQLRAPMVDVHELTNLLRQLDFKVVSLLDLSRREMQKAVNEFLLLLDKGVYGLLYYAGHGYENYGNSFMVPIDAPDSYTSEHCLCVQGILQRMQDKQTRLNVFLLDMCRKRNLHDGIIPRLEAFRVTANIVFGYATCLDAEAYEISHGNRSNGIFISFLKKRLLEDEKITVLLDKTGCLIHLPDVVSDMGTYELTKGKQALEIRSSLSERRALTDHIQQQEPPLRPAPALRTPHEIAAPKLPESRFVTFDCGVSVRLGFAAEFSNIMMIYTQIVQIPDGILHCEATLTDFSEDLDVDIKQTNKDCPEETGSVLFTFRNFDCADRCLYTRLSKLQKLRKELRFCVCLQYCYQGLLESVVEKQMVDVGKPLVAKLNLHD

>CmPCASP3

MVGATQPALAEKKPGMYICRVSDQQDHYVFSSWARIKVHPIKSGLPHAWQGSLVIGLQPESQTVRVGHRASLRCIAFGIPAPSYQWYRNGTPLPHHRKEEMLIPHTELRDQGTYLCAVTSDRGGEECWSCPADVSVGNYQPLSSSPLATTDKVALLIGNMNYTNHPRLLATMMDVFELSHLLQQLGFRVLSLLDLSKEELLGAVGHFLQLLDSGVYAVFYYAGHGYECSGRNYIVPINAPNPYKPENCINVQKVLWKMQEKKTALNVVLLDMCRKWYNQNYIPSEAQALAPTGNIVYGYATCEDAEAYEVQDGEHSSGIFMKYLKKHILKKEKVTQILENVSEDMGRDPLIVGKQVMEIRHSLKERRALTDQICSSGRTAQLTARNQCWSQANELPGRRTIVFPCGAEVQISCCAVFSNVMVVNASVKTPGPWAASAKVSFRKPADLEDIFSDLEGGSDSLLSSAVSEHQSDTVLKLCGLHRLQRPLTVPVVLQYRAWPENLRKEEMVEVDLGRPLVAKLELYKMLHGTRIEGASPSPTPSPYSSPCPSPTPGVPKSRSRAWGPGRGQTPSPWEAEASLSTGATSNNQPEENDESETHSEVTR

>PmPCASP3

GSLCVALRREEVPRGGPMEPSDAAGLDGLSPGVAARLCAVLDANGGRGWRGLARAHNFRINSPRGRGLLRHGECPRLPREDELEIGEPGPSARLLSALGARGVRVSALARALGAMGRDDAVRALGQEPVFVEWRSESRQAWRGESVTLECRARGNPPPRFTWFHGKHEIPDAVGPELLLVSVSPRDSGHYTCRVHNEISYAFTDWIRLRVLGDQTTPGHCNAQPTTISATSICNSVVAEADNSPASLVMVRCSACWIVFIFKHIQFDLAASCGGWFIRPHLARSSRGLTRKLIINPTSSCENLHVTAIGTLSDSLACSSSDSLRAHSFVITATEKVALVIGNLSYRNHTALRAPLVDARDLTSLLRVLDFRVVTLIDLDLQHMRLAVARFLELLDEGVYGLLYFAGHGFENYGQSYLVPVDAPRPYLSEHALSATTILRGMQARRTGLNVLLLDMCRKRNVHECTVPPVETLEVTANIVFGYATCQDAEAYELDVGPGEKAGVANGLFMRFLKERLLEDRKVTAMLDSVAEDMGRCNLARGRQALEIRSSLSERRALTDPRRPAHGFPARSIYQRWAQAHELPPSRTREFDSGCLVHLGFAAEFSNVLVIYARVLERGAGVVSCRAYVTDLP

>CiPCASP3

MHTTSSIRNLILISEVPQEILNRLISYLRNDGESGWRTLVYHLQQRDHLFSEDEINSFETIHAPTLVIEGLKKRNKTVDYLLSFLHLLPCRCAAEKLANDLWMLEKSQQKFMSTPMPECSKYSSVGPSYVPNGHSTHAQPNHLFDQAMFSSLNNLNKPSKPYISIMKQPTPQSVKIGDDLMLVCKAEGDPVPVHYQWYKDGHMLPNQNKHVLMIGETLLEHQGFYYCHLRSPNPPAVEKRTDEVFVSVVLAPSATPFCATNKVALLIANEHYAHNPRLPATSHDIRALTKILQEDLGFKTLSFTNLTYTNMMSAFILFKDLVSSGCYCVFYFGGHGYENDGKCFLVPIEAPPDYQNHDCVQAQSELENVKRRGDPNLMLILLDICRKRNPMNPTSHQSNGSSFADKVVFAYATTESAEAYEKYGEKHGIFVDSLRKHLTTPIRVNHMLSDVQDEIKKSDVQISEVKSTLSENRCLTDKIQLNDVKAIKAYKEVDRIWNSLHFTPLPVLLKFEACDAEVTISFTNILTNAVNISVNVIKCGKGVECKPFICTNYFGNLVVSHCSSEFEPQSCQVSNLEHLQEHLPVTVGFRYKFPSEEQWHLLRYTYSMRLPFIKLWNDAKPKRS

>BfPCASP3

MATLQSGGSPSKLLLQDLGRRGKTVRQLISYLEKLQHERALMLLKQEEQLQITRQPESVSSMEGDTVELKCQAIGFPYPRYQWYHGNREITTGTDSVLRMEEVRPEDAGQYICRIHKVSSRDPRKPQSYVFTEWVQLTVIPTSNYVFSFEYSPLKTATYASEQYDDPPHIAVHPSPVRVALNGPVSLSCGARGKPAPFYQWYKDNVLLQGETRREIYYDQAKASDQGVYTCRVWNRVGEVWSQGAQVTVGEYMTGAVCPEKPVERYYATDKVALVIGNQYYRNQDNLEASINDAYTLSNILRNELDFKVVSLTNLDLQDMTNAVQAFCELLNEGVYGVFYYGGHGYENLGQQFMMPIDVPTTYRASDCVCAQMVLKKMQEKSTGLNLLLLDMCRMRYCNMHDNSDVISWDIERPRGNVVFGFATCSNAEAFEVQGERNGIFMSYLKRYITQPLRVTHMLEQVQKGVGQDKQASHVQFPQIMADLREDRTLRDPISFHGHTQEFCVRNQRWAAAHELPEAINICFQSCGVHVKLGFFAEFSNVMVMYLTVLDLGKTQQCLPSLRDFPPVGTLS

>SkPCASP

MSGVSVHLNICDIPYNIQLKLQTFLDVLDTNKNWRGLIAAIPDNPYSAQEIDMFGLDILRFGSSPTASLLRDLGNKGRTVKQLISYLEVLKHEPALMLLKKEEPISIISQPQAVNIQNGGTLELSCNAMGFPYPKYVWFRTVDKEINGNRIRQSIEVENGCDRVLRIEQITTDYNGMYICRIHNRISYQFTNWVHVKVIAKTDPPVIIEQPRSQCIEIGRPLKLTCEAIGRPAPKYQWFRNEQHMVEQTSSEIYYDVSASQDAAKYRCKVYNNSGEVWSDTVQITIIQAGPPVIRRQPDSVRCPLGHDVSFHCEVTGIEPISYQWYDAKSLIRGAIKSELCINKVKYQHRGWYQCHIRNGYGDVITEKATLEIESRPPLPPPQQLTQYFATDKVALVIGNQDYRCEQQLKAPVKDAYTITEILTTECNYKVVSLIDLTLSEMEAAVSAFSELLDSGVYGLFYFAGHGFETQGQSYLLPVDAPSYYTTEDCLCAQYILDKMQQRNTGLNVLLLDICRKENVCNGAKPENVIPYKPRVCGNTVFGYASSPNTQAFELKDASNGIFMKYLKLHLASRLKVNTMLDKVIEAIKDDKDVREKQYPEVKSDLRDHRTLRDEIISDGHTQEFNIRRMRWEEAHLMPMNPLLVAFDLQTFQVKVKIHFTAEFSNVMLIYVQVVDPGKATRCIATLHNFPKELDVNIKYANRSYNDTIADCTYPRLQLPVKDKSLITRIKNIQKLTSDLELTVILDMVYEGQKIQSTCPALKIPQPGISKSKIFQRRPVEQSCPLK

>PfPCASP

MSKDLSPHTNINDVPYAVLLKLHTLLDIGDRRNNWRAFIAAIPDNPYREISDVETFGLEQLSGRSPTASLLRDLGNRGRTVGQIISYLESMKHEKALKLLKREVERNPGPKVGRASTSTALGIDLATKDNILGINEKLDDIIFALEYVQKKLDSLQMRLENIEDDVPVLDEQLQENSTKVEPVRIIKQPVSISLMANDTLTLTCEASGFPYPSYQWFRTVIEEVNGKPQHKSLKINNGTDKVLEIPDVSDYDTALYICRVHNRISFQNSSWVKVDVISKHVPKGPPVIMRQPTSVDCVVGDKVVFTCEVRGKEPLYYQWFKDGKKLAGENKKELCFKESQHRDRGEYECQIRNANGGVTSQPVRLMFMNCGLPPPEPQHATDKVALVIANEEYRSEVRLHAPGRDAKTIHRILTEQCGFKVVSLIDLTKEEMWTAVHAFCDLLGKGVYGLFYFAGHGFDANGQSYLVPVDARSAYGIEHCLCAQSILEEMQKQNTGLNVMLLDICRTQNEYIRDAPVKTYKPEVKGNTVFGYASSFTTGAFEVSNAENGIFMKYLKKHLANDEKVTVMLDRVIAEVPIDSPVCVKISLDLGSETIHVIVKMSFYAEFSNVLWIDVHVEDPGTTSQCKVKIQDFPPELDVSIKYLDRQYGNTVCDPRHTHQTINNKILRATVKNIQKLYLRV

>CgPCASP

MLSKQQCRNLTVDPDTNICDLSSKLMLDLREYLDNGPETSNWRAFVSATARLYRKYTFSYQDIERIASRVHRDSPSKALINELGALNMTAEELARVLDQLKLEKILMYIKKYDQIKITSQPAETLELTEGDLLRLEVDGVGFPYPRYQWFRLNPSTSKYQEVHGQNLKVLKIDKCRFDDSGTYCCRLHNGKGDTQVHFSGESVVVVKSRPLKKAGYEEDTSACTTDTNHHEVPYNYSTALDRTPFYRQKDVMPEKAVLPYILEHPKSQQIMKGSTLIMNCQALGKPPLIYQWYKNGQFFTTTDESFIKIPALSSQIGAYFCLVKNAFGEVKSKEAIVTVSENVPEPVVQAEIEIISNPKSCTVEYGGSCIFSCEARCRGLQLCYEWYKDGRKLQGHCSSEMRLDRLQDMEQQGNYMCMVSVPELNLSRHSKIACLTIKMDPLKRPEFNPTDKVALLIGNYDYKNETELKAPETDVSTLAQLFRHLDFKVVTLLNLTKTEMLSAVDYFSELLGKGVYAVFYFCGHGFEEDGKCYFVPPDAKAGYTIEDCVSAENVLSRMQKPDPDLIVLILDICRIRNRVESSHAADMINIQQRGNTVFCYATSLGMFAYEDAVNGILVKYLQKFIVQDKSVIEIFSQVQEAIGKEPKHYKLQIPEIRSNLLQPKRSLADKISTKGATKLYQQRTLRWLECHQKPEKKLIHIQDLQMDLEMEFQSDFSNVLHVITTVKNPGITLQAVAYICKIPSAVSLKDMNPKVTSNNPTRTKSTLQEIQKVTEDLVIDVGIRYALAPEDQAVSPQYKLHIVQVNLGLPLVAVLKLWKPIENEAMEHEDFSDKNGYS

>ObPCASP

MASDFAGVAFKITQFSPSQLLQEGDVLNLWCNATNTCSQLTYNWFKDGQLIATTQDYRKEKTSIHDSGIYWCEVISNQGSISSEKITINVSADTLEIVIQPKFTMVLLNGFALFLCEAKGPAEIHYQWYKDDTILPNENKTELKIHVESESFKGMYYCVVSCLTATRTSDFAYLQITKSVPENISKEVCQATDKIALLIGNEDYRSERKLKATSKDVKLLSGIFERLGFKVLSLLNLTLTEMKMAVYHFVELLNNGVYGVFYYAGHGFEVSGDPYSYLLPVDAPTGADITACLPAQKIHQLMLEKNPALCCLILDICRTICEEAKYYSLPLEIKPNTSSLYMYSTSRGQSAFEPLNGRMGNFVYVLKDYIYSDDTVETMFGKVRRAIAINSKILQEQGKRCGNQNPELFSNLANVNISLADQINTTNHTQEYNIRSELWMQANRLPRMTYVDIKEFGAKLKIEFSAQFSNVMSITVTIVDSGTVPDCYAWIANIPSTLTADKATVLQDDKKKIRIVVKDLQKLQEEMLAIIRFVYTDESGNSRCRVEDLHLDFPLAANLELCKLSTELLEMNCIQKKMS

>BgPCASP

MCASFLTISVFKYIHSIYCCSIKDEGEYKCAAYNDEDILKHIFSTSCKVYVEPVQPVPPLEIKEHPRPCFVFTGGTAVFKCEVWSSAQLSYQWYKDEQKLQDGLCVQGSSTEELRIKTNEMDSLQWEGNYFCQISLLHPCINYPYEYVFTQAAALKVQPKPNPSVQSNKFTYNATDKVALLIGCYDYRSDRLLSAPGYDVQTLSSIFQSMNFKVVSLLNLTKSELLAAVDEFSKLVWKGVYCVFYFCGHGYEVSNQCFLVPVDAPFMYQHPECVAANSIFDCLLKRDPQICCMILDICRKPYIGQPPEVPLSQQTYEKGNAIVCYGTSHGLAAYESKNYGILVKHLKNIVGQPLDIESVFRKLREAINEDPMLNNNGSKQAYKQIPEVRTNLLEPKRSFTDPIDIKGNTEIYHDKTAIWELAHRKPDSLNLDVEFDNFIVKVKLDFQQEFSNVLKIYTSILDPGPTQSCVAYVSGVPADVAEKAKVRTISSGLDNRLKKNFVVIHNIQRLKKPMKINVTISCKVPDCKKTMESIDLGLPLIARLNLWKERPDFLSQRFAEEVAENDDSIL

>ApcPCASP

MNDLNAIGDQNLGQLPGLIYLKVRDHLDISPTVQHGWRAFVAVLDGDFTIRSDIDVAAIERVKSHNSCAAKLFNMLGARGMTIRQFVHYACLAEDNFIMDLFNLQAAIKIIDHPPEEVHVKEYDPLRLTVKASGRPQPRYQWYKNNNRMDGETQSILSFDETHGSDAGVYICVVHSEDMKQCLRCEPTTVIVDVESSDLEVKENPSSCFVVHSGNALFRCEVWGAENLTYQWYKGETALVDGQDVKGSQTRELRIYNIKSVDWQGWYYCEVSSRSQTVTTRGALLQLVSRMKKHHETTEYTATDKVVLLIGCSDYRGDKLLTAPINDVQALATSFQALNFKVVSLLNLTKLEIISAVHEFKELVSEGVYCVFYFCGHGFEGSSGLRYLVPSDAPLGYDNTHCIAAEQIFHVLLQKKPGNCCMILDTCRRIRPESKPEHNHFSAVETGNAIVCYATSYGLCAYEERDRGILVSHLKDILGEPIPIEHVFTRLRESLGKDTQVNVDNPSNPHKQIPDVKTNLSEPGRSFADSIVYTGYTSEFNRRQLAWHAAHKKPDARELEFSFADFTVTVQLDFQPEFSNILKVYMSVTDPGPTEECIAYVSGVPTDVGEKMQNKIISTGRSCQLNKTYVTITNIQRLKSPMNLEVTVKCKRASDQSHLEKAVRTDLGWPLVSSLHLWRERPDLSLRREAVEQEESSAGDESRDFYG

>LaPCASP

MFKSEDRTYLIKKDTNICDIDFLILKKLFNYLDASPSSTQGWRDLVAAIPDSPFRPEEVEQIAMCIHSHQSPSQKLLQRLGTRGRTVEQLVSYLWAMKNEKCLELLIAHEPVKICKEPVASVVLEEGDTLTLECEATGFPFPRYQWFNGQAEVLSGSESLLCIPAIKLRDKGRYVCRVHNRGNEGVCFSKPAMVQVKPCNYNQDQGCENLYSRTPTESYVTPLHPNAPYIARQPELNDNRIIAGKPFEIVCDASGQSPLKYQWYRNGQPLHVETGMRFYCSGASTDHSGIYQCVVSNRYGEARSKEVQVQVELAYGSQPVPISPTITKSPKACKLEEGGTTVLNCEAIGMEPIAFQWYKDGKPIEGHAGPRLELNNVTVDDKGIYQCSVSNRHGKALSKVAKVEVFCSDNREFTATDKVVLLIGNHHYRCEKNLIAPPNDIRDLQNLFEEMNFKVVSLINLTKEQILNAVDEFCKLLFKGVYGVFYFAGHGFEKQGQCYLVPSDASTGYGANDCVCAEYVLKKMQEMNPALAVMMLDICRKENVHATESVEIYKPAVRGNMVFAYATSFSMEAYEEEPLMPVDTSLCSVNGIFVKHLKKLIKQKKMVSTMFQEVAQAVNKDALAKEVQFPEVRTNLNEARSFTDPIDFRGSTQRFDKNLLRWQQSWECPSSTTYRSPESNIIVKLDFEAAFCNQLEVHTTVLDEAACSNVAAWVEHLVDAYSTQNRCKKSCKSSRPYIAKSEFNDIQNLRGPLHAVLFLQYKVYEESHTEKAHIELGEPLVVPLLLSRPRVLANQRVPMEQEEVIYHVNR

>CtPCASP

MPLDINPRDFLQGIPPKKLLKIIEVLQSRWADVLEHITEDDVRSPGDFNLEDCRSNRTGKDQARYMLNWLGNRGMRISCLIRVLDELRLERALICLKDPESLQIVEQPKSFLEVYEGDTIKMTCTAIGFPYPRYTWFKNRQEVSSGLNGTLEIRDAREDHSGHYMCRIHNEGSSNILWSRNGRWSEVSVKEAVLRTPPFFTLQPPEKLNVNLGASFYLECCVESATNISYQWYKDGKEIQNADKALLEISSASHDLGGEYHCCVENKYGSAQSISCLVRIYREPSTYREIIPSFSPHNPIPADETNIIEHPVNQTTVIGGRAFFSVRVDNPFNVSYQWFHNGKPMPNQTLNTLIFDVRSKEQKGTYQCSVERLNNKKQQVCQLLSLEAKLEVNMSRLVKTAEDKVALILANADFSNQKDLPGVYNDTIETTKELEKLNFKVYSYLNLTIEEMWAALIIFSATISEGVYVLVYLAGHGFIYHNQEYLMPSNALKEKSYADDNLCIARVINYLQKKKPGLMVVVTECCLKQGTEYIIEVPGEECEVLKTCATNYIIHSNGPEERAFEEKNVAFPRGLFNTYFLKAIKQELTLPDMLKSLKKDFDDRPYDKVKYPMTINHNTDLQDEELGLHDPIKVTDMARMNDRSARWEAVHCIPAIKAIRFECGGALHFECYKKTTNLLDMRYRQEIQSGSPLKAIVVRQIEVMSKGVEGEKIWPNYFRFYNLQKLQGALILKVSGCLILHQSASHPQGNRIDFDETLDLQRPLISKKQDYWRDSISRMPGAKNPDQTTYQRQATEQTEPETDSLNYVTGEFDTV

>DpPCASP

MNTTVELGFEVSSDGFPKPKFQWFLNDAPLASESATFNPLVIPNFRNSDAGFYKCEITQQCRDGSFQTISTGTSKMTLENCAPFVVTEPQDLCTDPGNVIELSCMIEGNPIPRFQWYKNDYPISNEVTTILKISSAVPQHSGRYRCLATNSEGKTLTKEVFVEVRLPPTSQRLRAAFSDVPTQHSASMKQALLIGNWTYDAHGLRKVRADVKTLHDILYKMEFEVISLCNLGKVEILHAADRLCELLLPDAYVVVYFAGHGIHYSGHDYLMPVDLKMNSEYSVRDLVDGNEIIARIQQRHPKLLVVLLDMCRTIPQQGINSRIADELLRQVPRDSRSNLVMAWATAENSFAYEMGYEENGIFMTYLKRAMENRNILVTDMLERLKQNFKQEIPIEAKDQMPITSSSLAEPLSLYDPVDGSVIGGSLWNDCQMLAISWKELPQKQIATIQTNGWVIHIYIQFGMMDDCFSNGLKIELTTEGYDKITFQPSIHPPVLELLEVKHQMDLKKCTITLHKLQALMEDASLVLRIAEFPNERILVHFGQPLIAVARLSANYF

>NavPCASP

MLKFTEDLSIEHLPLDVYKDLVAELNRDDSWRTLAIYVSEKLDYSQYDNSAWVNGLEDNNGSTNDTPASKLLVELNIRLCTVGILSMLLEDCNLLKILAILHTPDPVRIITHPDEETLPSEDLCVSLRNKVRLICRAYGLPPPNYQWYHEDVRLEGETSEELNILMTSTSQQGSYRCKVYQINLKGEKLSEIFSRPLHIEKFTAKDEGKYYCHIFNGISEKWNYLVKRLXXXXXXXXXXXXXXXXXQEILRTPKNDVAKVAKILDGLGFTTICLTNLTLLQMKNAIKIFSDSLVEGTYGLFYFAGHGFKMQENYMLSIDSPTNYLRKDAICESELLSVALQNDPALLVVILDMCQTIPPIEFNPEIHGEIPSINTYKGQKNLRNLLQAYSTSCYRPSYERMGKDYGLYATHLCKYLDENIPVTKLFEQVGKSIDTWFKGAERKQIPMFATTVTKPYKLTDSIVQSALPHSIVQLEKLIKFSNKVIDINFQQTGVKCKAKISQYAKPCLNCIQISVIDLDDKWEVNFFNSVHTKRNNLFKCASKNKCWFQSPETNEGPLVISLLDNGVPVGATLLEINELVPTFLYRLNQVNA

>ZnPCASP

MSKAPHPDVVVTNLEFEIARRLRVLLDTNDSWKLLAEGFNKCYGIFSACDIRMFQSKSSPSEMLLHEIGNRFCTVGVLCEILLNCQLYDALALLREPERVVITQQPSDDEDTIYIPEGTDLRLECRAFGLPPPKYVWFQGNEELKAQTSSILLIRDFSVQHEGEYYCCVSQDVDGEEVCQVYSHTVNVELLPVPPTIHEHPVPCVVFREGDVITLSCFATGYPEPQYEWFKENFQLPGEETNTLTIGGVGASCEGSYRCYVKNRGGAVWSNHSNLSMIVPNPDDESRTRITATEKVALLIGNDTYTDLSDLNTPRNDVATIASILKDIGFKVIALHNLTLNEMRNAVYEFCQLLPQEGYGFFYFVGHGFEIQDKFMLPVDAPGSNEYRRCNSLCEKEVIRDVMKVKPKLFLILLDMCLKIPSRNENPGIHEELPKAFLYEPKRNLIQQYATTSNLGAYERSTEVNGIYVNHLKKHLEKNITILNILKMVQKEIANESADAADKQMPLTSSNVVDDFCLTDSVTGHPETEIRFQKLMALPAVDTIVFDNIGWKSVVKIQPHRDCFHNSLDIYITNMAQWAVVCCMACEKLKLELHREGDVLVITIHNLQKTKMPIVLSVILKDPESKKEVSSYTFDLEMPLITQAHLWFNCSRPSSP

>LpPCASP

MDYNSCILDINTGIYQKLCTVLDRDSLWKQLVENIPCVAERISVEEISQKYKQKPTWMLLYELGSYGLTLGELVEWLKYLQLEAPLLLLIADEPLEIITQPTPYVEVEEDQNNGLTLVCEARGFPFPQYTWFHDGEELETSKNSVLHIFPVKIEHKGSYFCQVFNYNRQTEGFNKVISTPVEVKVKPKHQSFYFFELPEIIAHPINPSNGKTSQGSSLLLNCTASSKLPVHYQWYRNRAIMSGETRSDLELRNIYPKDGVHKWTYQCQVSNDCGSVLSKEVVVELDSPNDDTPYYAVDKIALLIANANYMDNIWKPVPASIADVREMSDLLTKAGFRIFAFQNLTLDEMKNAVSLFCSQLRKGVYGLFFYVGHGFEHYKQNFLVPVDSSCNMNPSENLCAQFVAECMVEKNTALNVLLLDTCRFQLPNSNPEKIYISEGMRNTIYGFSTGSNHYSCEVRGEKNGKYVKYLKKYLLQKEPIYGILLKVQEGQLKVNLYVYKASYIKSE

>AmPCASP

MTRFDKDAYIECLPITTYNEIINALNKDATWTILANHVAKELQYPCTWLQSLQEIKHSNDSPGQKLFSELNIRMCTIEVLCTLLNDCKLYNILSIISDPEPLNIIMHPTEKFPINILKVSFGHHLHLCCKAVGMPPPNYIWYHNDKQLQHYTSDELDLIITNVSQAGEYKCKVFQIKNDGTLISTLTSKAVIVQIFPMPVIIEVQPQQFLEVKENESFTIFCKASSNPEPCYQWFHDNIKIDGETSNILHIKQFTSKNEGKYYCYIHNNISEAYTEKSHIMVKIYFQRLKAVAKIALIIANEEYEYHKCLLTPKNDAACIANLLKEIGFEVICFLNLTFTQMKNAIEIFSKALMEGVYGLFYFAGHGFKMQESYMLATDAPEKFLRKDAICESELLSAFLKNNPELLIIILDTCQSLPSRELNPEIYQEVPIVKEYKSKKNLRNLIQAYSTSSYRPTYEKIDSKYSLYMTHLSKYINKDITVTKLFEEVGKSIDSCFKDKERNQIPMFAASVTKPFRLIDAIYKQNRPNIIDHLYELTSYSSRTINVTFKRSNICTRITISLFMELYLNVIKIKAHDLSNVKVTFYNSVPMKRNNLFQNLYEKECWIHNPQLTEKPLIITISKNEILLGATELHIKDYIPSILKDINV

>SiPCASP

MSATSAFLFNVYATQADNSFVRTSELIRWLELVDAIDALLLLKPSEPVVITLQPEPYLQLENGEESVLRLTCRASSFPPPKYEWYFNGKPLISRIQAGPVLELFNLTTDHSGTYKCRAWNACGSESFSNDCQVVVTPCRRKLVNNPPTLYATDKVALLISNANYEHDEKLFLTENDVESLAKSLAALNFRILAFKDLGKRDIINAVRTFCYFLQNGTYSLFYYAGHGFKCCGKDYIQSIDCSNECSVEDCVCFQDVIACIKSTPSSFNLIMWDACRDCRETSLDSRTSIVQTDPPHGSLRARRSSVVVYATSPYNSAVEVKGHQNGLFMEHLKRHVGRTVPVHEAIRCTLRDFESHALSDWQIPVVKYDIAGDHSLCDAILDGPGYRGNETLWNFVSTLPPDEEFRVTETGLRLRVTFSCDREVFCNSMLVTASLLPPVPSIHIDLQTLVCDGARAVNLSTAVLRVSGLQTLVSPLRLSVVLRDWESKNVVYLRNLHCGHPLVAAWKLRNTG

>SmPCASP

MDLDLNILDLDYPTFYKIRSILDVKSTREDFLAAFPPGYFSTEFLFTMDVEAFKMSKTPAEYLLLHLGSRGVCVYHLIELFEAINLEKALVILKEPEPLEIVEQPPTRLELREGAKLLITCKATSYPPPEYHWFKDDEELYLQQDPDLSIASVKLNDSGIYRCKVISHNANTNENSCIVSEPAEIIVFSNETSVKSCAVSTLQVPKSSHFMTSEKNDCQFETTETPRDDKKAIKIEKQPYVNGKVPQGMILCLNCEVSSAYPVNYQWYRNGEIIDGQTSSKLELNYLYPDQNGDMKWQFFCKIFNEYHETFSETVVVELQDADETTPYFAEEKVAFMIANDTYDNLEELKKPAPDVADLAKLLSSMDFKIFAFRNLNYTEMKNAFHTFCQMLKPNSYVLFYYAGHGFESCGQTYLQPTDCSFESLLDESICSEYILKYMQDTKPAVNILLIDACRKLPFQFKETCEKECQSGKIFEAYMKNNTIYGYSTSKLCASHESDSEPNGFFAKHLKKYITQNKKIQDVLMKVQTDFAGDSQLKNIQSPVICTSLSAPRKLCDRLRKPTAVNGILSWADIKRPHAYKKFDLAKLKSTIVLAFQHHMDVFCNAFDVTVYIDTKEDAPLKILCDMEVNILFNSPDIKVIPIEGIHLRDTPLCLDARNILVRKEIHDVHKMQSPLLDGKLLLRFCMQDKQIATEFSMPVGKLAELKLSINQLKSNEETKIKAGKPKDLNLFNSVSSEVDNDFLDH

>PtPCASP

MKWQYYCLAFDDYDREFSNTVEVELEEEKDSTTYYATGKYAFMIANDTYANLQHLKKPARDISDLAILLEEMDFEVYAYRNLNLAEIKNAFKNFYTKLEPGCYVLFYFAGHAFEQFGQVYLQPVDCSEKYDPLKAVCAEEILQCLQTAKPALNVFLIDACRKLPDHIRNGEKLTLNVQEAENFKIFDGKMGRNTIFGYSTPFLYSAYECDSEPNSFFVKNLKKHITKNKRINDILDLVQQDFMKEEKVKNRQFPEIRTTLSGNRKLCDEIKKCTNKSLDLDFTLKKFRENLAPSPTEIPLSSAGSTIIIKASPYLDVFMNDIDILVSIFINMKAPLNILDEMEIDLISADEEKIKLLPATSVCLRNIEKKPSENNFLLKKQAFGIQKFQGETIDCKLLIKFPRAKKCIEASVKINVKSITDLKLFVNHLGTLIIEGNSL

>LsPCASP

MMVFYYSSSRDGIPYNWPLWKIKREKSEEWNAVVQMISSVKYELLEKILKDDDVKAIVNGCPNFSKNFKEDQFSSSLINLFANRSQKTFGWLTTLCLNFFGAKSSTISFLSDLHEIYKPITILKDLSPDLISIPLGSSTTLEIDALGIPYVHYQWLFMPYDEEGNLKKWIRIDVDSQCKILDIFNIQLSDCGLYRCRIYHNIPVKTPDDKCAQDVLSSCVQIKVEVGSINIINQSSDVTSFLCGSAEFDITAESTHPLQYEWYFEDTKVFSQDDHNGKLKLANLRCEQSGRYKCKISNEFQAVESDVVKLVIDIPSLEDIQYNNITFENENIQILKQPSFNSSSRVNIGDKISLEIVASCKYYLNYEWCKRGLKQDLIDRNEPEERTDLILVCQGPQLVDIVTEAPKCTSYGFIGTWVYICLITCPRTNERIVSQSVNVPVSFCSSIPNSFPGFKIALIVCQEDYKTYDFHELQAPKYDGSVLMTALKEMDFKIFSFINLTTEEIRSAVELLCSFIDDTTYVLFYYNGHALGSGTNVYLTGINSSLSGSLKEFIWHGDLESKIDSCGPLLCVFLYDSCRDDPPKYFQEKLQNTKHPAISFKSSFVISYGTQPSMKSFECYEKSLGACQGLYMKYLLQHIRENIRVEEVFIKLSDSFMSGEDTSVVTKMRPEIKVSTRQAFYLCAPLRSNTCNTKQVLFFQMCRFESCVLSADVSSIFDNQFKYVIGLSYNKENKTFVWITGASNTNALNVLSYIVKITVRESNFTNEAILKLHINKTSPKIDNICKNFELQLSSPNVELISTTKNQNSHAPITVLFEPCRSKNVQRRDGPIFPLMDERDDILFDEIKLVNLQKVEGNAKLRLNLRTENDSVIVPIKGVLEIPLPILHNFPCLKLNSKSVLQCIK

>CePCASP

MNTNLAELPVRIFKILSEELQKDNIWIKIVEFDEDPIYYMKSTEIESFLKREKCCEQVLRKWGNRGQTVGSLLARLQFLSRTYEDEFDSIQFHLRRKFKPLRWIPDPEQQVTKEIDEGNIKLECKAQGFPCPEIKWFTKGSKEPVHIGRVYTILRCKCSNEHQYKCVAKNEIREGSPYSEIYRKAGKQFSSVIESEYVDVTSCIRDDELCESCKKYEMGRLSQILAEDQEKPENKVSPVRPNLDITLRAADKVALIMSNCSYVHLPELRTPHCDAQTLADALQKMNYKTVTLADLTLDEMRYFIRVYQKLIGNGVYAVFYFVGHGFEVNGQCYLLGVDAPADAHQPQHSMSMDWLLSIFRHKTPDLNLLLLDVCRKFVPYDAISAFVEYSEQFKKFHRAHRNMVYGYSTSGGVGAYEVKGEVNGVFMKYLKNHVQLEISVIDMLNKVLLDIGDDQKVCDLQVPEIRSTLTHPRSLADPLIFDGHTASFDNHTIHWRLMHELPTPATIRFETQQLVATIWFQFCGNFTNKVYVFASISDFRPCQEDTDMGENEELSENALNHRAFVEFPEELHCSDVREYNDDEEGVSMLWILSGLQKIKKEAGLTCEVHLRHVDDPEKTIEMKNVDIGHILITRIKCLQ

>DvPCASP

MTSLETNLVDLNLKTSQLLANCLSSKDLWLKLVDKNKNSMYYMRWVINKNELKRALKRHMLLFEKIWRESLLPSFCAVRLHYVNSLKSNLNSEDDVRRISREANPGEAVLRAWGNRGQSVYDLLVRLQVLSRHYGSLMDDAQLILSRKFKPVRWTKSDEIVVTFIQENVRLQCKAVGFPTPSYHWYRNDDLVEGATSYVVDILRCQCSSDYKFYCVVTNEVEDGHVYSEFYRKPGKQYFSRIVSKTLDLAPIIGKEHRCGKCRTEGLENWKEVITNMNVLVENPPLMAAQHGNDALVAADKVALIISNCMYKNLPKLVTPHCDAETLASVLQDLKYKTVTLADLTLAEMKFIIKEYRKLLGNGVYAVFYFVGHGFEVNGQCYLLPIDAPLDVYKPEYCLSMDYVLYELNDYHPDLNLILLDICRKFIPYEFIHPFVEYAETYKSKHRPNRNTVFGYSTSEGVGAYEIKGEMNGVFMKYLKEHLHRPISVIQMLNDASRDIEEDEKVCDVQVPELRSTLTKPRSLTDPLIWDGHTASFNHHTVHWRLMHELPNPVYVRFVDLSLTVTIWFDYCGHFTNKVYVFSSVGDLVDEAVEETENKELSENALSHIAFLSFPPEIDASKERIVSDDDEGVSLCLLISNLQRSKGELRCKILLNTIADVETIVATREVVLGHVLITRIEMK

>HcPCASP

MSCSNYDELSEDEVERIGREANPGEHVLRAWGNRGQSVRDLLVRLQALSKHHGAAMDQAQLILSRKFKPLRWAKTEEIVVSTIQDNILRLQCRAVGFPSPQYQWYKNGEIMEDALSDVIDVIRCKCSSEFTFYCVATNEVEDGHIYSEFYRKPGKQYCSRIVSRTISLEAFVGQDQSMCIMFLFLYKLSSHFSSRVRTSSECENCRNGDMENLRELMASMNTSAIEEPPELPEVDNAEALVAADKVALIISNCMYQHLPKLATPLCDAETLATALQDLKFKTVTLADLTLAEMKYLIKEYKKLLGNGVYAVFYFVGHGFEVNGQCYLLPVDAPSETYKPEDCISMDYVLHEFSDHNPALNLLLLDMCRKFIPFEHVPAFVEYAQPFLMKHKPNRNTVYGYSTSGGVGAYEIKGEMNGVFVKYLKKRIHQKISVIQLLNDTSRDIEEDAKVSDVQIPELRSTLTRPRCLTDPLVWDGHTVSFDHHTIHWRLMHELPNPVYVRFEELALFVTIWFDFCGHFTNKVYVFSSVSDLQEEPSEEFDKKLSENALSHIAYLSFPSELDASKERLLSDEDEGVSLCLLLSHLQRSKGELSCRILLKNVNDKDTVVATRDVVLGHVLITRIEMIR

>NvPCASPt1A

MRMGGSPTECLLQDMRTKCRTVKQLVSYLELMEHSEALELLKPDEPPVITEQPMSLPVQEGLKLELTCRATGFPPPQYQWVKNGAELPYGCDSTLTIDRVSRQDQGKYFCIISNRIGSVKTYEVEVNVLRVQRHEQEEETPLPESSSLPYITQQPQSALIPIGHRYHLTCGTKGPVKFQWFKDGFRLHEGQRPNLHFPTFNFQDEGNYTCRVSNTAGNALSATATLQALPDQLDINAGQHPDVGRSMELSQTAADKVALVIGNRDYINLPEDGSPLVHSCSDAQRLASVLRKNSMNFKVVSMVNLTRVEMDKAINLFLDLLGENVYGLLYFAGHGFEEQGQNYMVPIDAERDWAPDEAVCVQKVFEMMHARGTQLNIFLLDICRKRAVTSQAYKMETYSFPFDAQLVTGYATCKGSDAYERKSDSNGIYMKHLLNNITLDVKVEDVLHRVAADVEDEVAMDEYTERQRPQYDSKTSSSFSLRDPVDPKLSPQEEREQRSGIWLELHRLPEKHVIEHRGCRVEVRFEHTNWLSNVIVVCLRVLELGETKICDASLSLRNLPQDFQDCHFHPSTTNILRQVVPREYQDEIRARTWKIDSSPEKEPAPVPVERISEIYNIQRLKKNLTLTIKLKYVLRGCEEPESKYEIKAFNIKEFGIAMAFC

>NvPCASPt1B

MADSLCIILFGLKRNIQFRVSNDFLLLKDSRNIPKDLSSLQQKFTPLSGFTVRSIRSMAACCPTISDLPKVLREKLAHILDESNTPTWRELIHVMPPDMYRAHQVESFAMATLKCRSPTQELFDDLARRSVTLDELSGWVQKLPQSNKKQSMIQLLYGHPVVTRQPGNIMVAVGANAVLSCGATGHQPLNYQWFKSKKALEGQTGSLLRVKNVSKLDEGHYICRVANSNGDYVFTEWAKLSLQNPSETHQFSRPIITSQPEYPEVHTVLGGTLRLYCDALGDPAPTFQWYKNDLPLPDGDQREYCKRDICADDQGRYMCVASNSSGSTESRPTRVFVRHQVPRGQMEANDKVALLIGNKDYKMAQHLNTLFHPLNDVCDLAGVLRSIGFKVVSLVNLTLKEMEFALDEFYKLLVKDTYALFYFAGHGFEINGQGYLMPIDATERYHSQENLPAWKVLETMQNSEAKLNIIVLDCCRTTPNDKPIETIPVDNGCYQPIVTKENVLVIHGCLPQNSVFESEDERNGFIAKHLIKLIGQTKPLSKIKLEFSKAIHDEKIYDPEDRKQQVVHIHTTEIDDMCLADNTAVHMQGACAVSGSEGWKAAHGRYTQGFRYFRSSGGGGGPEIPEDPVTVHERDGVVVELVFMAEFSNALLVTARCRPDNYHVTFDMPECVSGCLVTKVTGKESTIPQRFSRAESAIRIADLQRLQVFLCLNMDRQEAWGLGRAHIVQPSP

>NvPCASPt2

MEFALDEFYKLLVKGTYALFYFAANDKVALLIGNKDYKMAQHLNTLFHPLNDVCDLAGVLRSIGFKVVSLVNLTLKEMEFALDEFYKLLVKGTYALFYFAGHGFEINGQGYLMPIDATERYHPQENLPAWKVLETMQNSEAKLNIIVLDCCRTTPNDKPTETIPVDNGCYQPIVTKANVLVIHGCLPQNSVFESEDEKNGFIAKHLIKLIGQNKPLSKIKLEFSKAIHDENIYDPEDRKQQVVHIHTTEIDDICLADNTAVHMQGACAVSGAKGWKAAHEIPEDPVTVHKRDGVVVELLFMAEFSNALIVTARCRPDNYHVTFDMPECVSGCLVTKVTGKESTIRQHFSRAESAIRIADLQRLQGNLEMVLVLELDNEEPRRICYTMREKPLFAKIACQN

>ApiPCASPt1A

MPVDTYTQNDVNQFDRLGMQRGKKTAEALLQNLGQKCITVKKLIVYLEAIGNNEALEQKKPDERVEITEQPSSSPVMAGERVELVCRATGFPPPEYQWLRNGEEIRNACEKKLIINNTQAKDSGRYSKKTSNRLSAEKSNCVEIQKKDRVVGCQPQKKSQHPISAVVPVGQQFKKTCQVENPKDCDYQWFKKKNKLRSETTNEIFFDSFNFEHEGNYNCRAYNTKGDTLTRLATLQAWAPEXQCPNKGIATDKIALVIGNRDYINLPPNESPLVHTCGDAQRVAXILRKNLMNFKVFSLVNLTYNEMKKALSIYYELLDRGVYGLLYFAGHGFEXSGQNYMIPIDAXTDWTPDKXXCVQKILQEMHNRGTALNVFLLDICRKLTMTGNGYKREIYPYPPDAQSIYGFATCPGAEAYERKADANGLYMTHXLKNITKEATVEDMLKKVAKDVEDEVANDPDVWRMRPQFYSKAVKNFSLRDPTVREDQESKXRNQKWLEIHKLPQKHIFNVAHVGITVEIRFEHTNWLSNVILVCLRVLDLGEATMCDASLILSALPKGLQECHFHPSTANKMRHVVPPQAESSTGVVARAASWASQEEXSHAPVPVERISEIYNIQRLQKNLSLTIELKYILRGSQEEQTKYEIKTFNLKEFGIVNAFAKANPTDDFQDYSLY

>ApiPCASPt1C

MYSSKVTVQTLISNLKPNVLDELGMYLNPRMPLKDFRTLAGKMGYTFMRVRNFERESNPTVSLLEDWWTSFGKKGESKTVTDLIRYLEEMRRDDAVDLLKPYEYTERAVQLPRCQHQKPVPFGTYSPDDLQQNRCPEENELLFNPDAPEAVVSGVQNKIENFIHPSSMSESRAQGRHPATVDQNSPLPRSEVPRPMEPPIVSDKVALVIGNKDYQCERLRGLFYPERDAQEIAKALSELGFKVVSLVNLTLSEMRIAVLSFCRLLGKCVYAVLYYGGHGYEDSGKNYLLPIDGDLMYKREDSLCAQEILQTMQDCDTALNLLIIDSCRIRLPDRGTRPTFIKRGAKGNNIFAYSCCSEQEAHEERDQENGLYAQHLLRHIHRNVRIENILMDVARDVSFSXNHYINQRPCHESDAYLDCRLTDPICPXALPNEFRERVALWEKAHMLPKPFVVLETDHIYLMAEFRANFSNVMEVMLIASNRTPFPLTRFVMDLSVPAPVRSEVGMLSGDVLEPGLELKQVFKLSALQKLPDKLMTNLNLFYDFKGRSVELRPIINLGCPLVSSVFTEWDWWVTCGRAPPTKSTQV

>HmPCASPt1A

MDSQINPNTQIRKLPYRILRDIAALLDIPGNRDWKALIAQMPDNKYTPAQISMFERQSLRMGGSPSYELLKDLGFQLMEVAELAAYLEAIDNQEALLLIKPYEHVTITMHPSSEVVQVGGSVTLHCQATGFPKPCYQWFHSNNCLKGQCHSTLKINNITMAMEGKYYCKVTNPVGSAYSNNADVHVVPMIAKIVNGITAPIYSEACKDENHNEPLKDVASTYVDSGQYYKLSSSTHANSYQWYKNGYPIPGECKKSLIFEPFHAGDEGHYSCKTNSNGHVEFSDKENLHLGIGSLSFKNKQFATAKLALVIANQNYMSPRNKSEQLVHPINDAKLLTEKLTSIGFKVTCLVDLIKKEMEYAVDGFCKLLDCAHGMYSLFYFSGHGFEVNGKTYLVPVDASASWTTDSAMSAENILSRIQLCKKTKLDVLLLDVCRVSTNIKEDLSSFAPFVPGAQSVIGYSTCPQSQSFERRNDKNGIYMKHLSKFICENMRVENLLFEVGAAVRKETESHHLTNTMSPSVKSTTTQEFSLCDKILQGTGFVSGERDPTVEWFAFHRLPSNRVIEFDMGVFVEVRFEYVCSNTCFCVLYVQKVGQTKNCRPFLQEISDDHSCIKYDNVEDSFTINQEFISKSIRFDSVTSLSTSVNQEDRERWTLLTSIQRLNGPCFMKVRVEFEFNDERYILTQKLVYTPEEFGIISFFNAGF

>HmPCASPt1B

MEEPLIKDLPDRLCWYIAFTLDKTSKPNWKTLISKIQNNLYDSKTINKFYMEVLSPHGSPSMKLLEDLGRKNKTVSQLVTWLECFVEWNELVSLLNRAKKNEIYPLILRQFPEESVFVGNEITLSVEASGEPPLYFQWFKGEKKLDGETKSVLQISPICPESVGNYICRVWNQFGFVFSKWIKVFDGVHLSNLPTITTHPRSSSFENGGTLRLYSDAVGNPPPMFQWFFNGKCLIGHCGRDLVIERATIDNEGLYQMQASNKYGTVTSLSSNVVCLKSSSNSLLEGRGVPSLTSIDTKVAPSNLPSQKVALLIGNGEYYQEEKLGKLVHPINDVHEIAETLTTIGFKVVSLVNLDLFAMKRAIDFFYEQLDSGTYGLFYFAGHGFEVNNEVYLMPVDATENFSVDENIPISRILRTLAIKHAKLSVLLIDCCRSKPEYNQPCISNPLPKLLDTTSDSMLPHTIQDKNVIIGFGCCSQSRVMESPVMNNGFYAKYLKEYIIKPIKVDDMLYEVARCIDSSNIIDPSSGKKQVMYRHSTVVEELKLTDEVLPSRFQTRLSNLWELAHIAPESPITILQNNSVTIKLSFTAEFSNVLLIHSVVENHNINEYCDVKFILPHKIGGAPVKIARICNLGSVLEFVRISNLERLEGDISIHLNVTYESFGVTKQYLTSYSLKEKPLYAKVA

>HmPCASPt1C

MQMVDLNDSLKQEIRCTDAKDLPADILAELSFILNPPLIGKDFRNLAGKMKKSFQVVRWLEMQKDPTASLLEHWWSDNGSRTVEDLIVLLKEIQRFDAVKLLEPHTHYIVRSRPPSTVSTSSKEPTEHDSSEISFYRSTDRQSICNTVPSELSTVTRDEGDPDDDLNFYLQSEEDEKLLRKISGPCETKDIHPIYIYNNLKFSQSDSSKSLSASSLSTKSPSASSSTISYCTDFYTNAHNSQHAQLNEVNKLNTVVQQQQHKVSLTREAPPIVNQKIISNESTAVVASDKIALLIGNRMYRHRSTLCNPSKDVEALTIALRELGFKVLSLVDLNLTEMRTVMLSFCKLLGPGVFAVFYFAGHGFEENGENFLIPVDASPYSRRDEFIAAQEVLREMQLRETALNLCIIDACRVIGNELNNSDASRSCRLGHGSSGNTIVAYSCQPATQAYEIVGEENGVYMTELLKHIKRDCRIEHILMDVNTAVNRNPIITQRPVFETDSADDCRLTCKIDGHANNALWQSKRFDMWSESSIPPEPQIFKKNEESVIFMFTYQALFSNKLRINIFMKNFNEQPLELYDVQLLNGSDLVIIDKPICNIVRPSNDEWTEIHDFIIKDIQKITSNLLLVFQLKYRKKIDTSDFFPLELKIDAGFPLISAHFREFHKWICEENFPGHKNTWV

>AdPCASPt1A

CLERIHSQDFSSRILINRFSTVSRIRLSIDSTTRNSYFSISHPATHDSEKEFAAWGQLVVWLKEIENDDALEILGYVEEVEITEQPKSQPVRAGGSIILRCKAKGFPKPQYQWYKDNQLIQDGIDMELTIDHAEMKDSGTYKCIVSNQTETVKSNCVEVQVLPFAQNGLDAQRSLPPQAKHPPVIIQQPQSCYVPVGHPFSLSCEVVESPVAYQWYKDGFLLLGETGPVLEFQPFYYRHEGNYTCRVENSAGDALSNIATLEASLPNGYGPLDFHSAVHGRPAHLKQKATDKIALVIGNRDYVHLPLDESPLVHTCSDAKMLASILRKPEMGFKVISLMNLTRDEMTEALKKFYSLLGEGVYGLLYFAGHGFEQGGQNYLVPVDAGHWIPEKAVCAQKVLKEMNKFHTELIVFLLDICRKRANVDTSFTLECHDFPHDAQAVLGFATCPQSEAYERRQDPNGMYMKHLLKHITKDVKVEDVLHEVAGDVEAEVESNQWINRQRPQYHTTTTRSYSLCDQIAPDQSSEGRQKLWHEIHRLPHKREIEIDSGIWIELFFEHLNWLSNAVVICLRVAELGEATMCEANLNYDSLPKDVQNIHFHRTTTNVLRQVVQNSPEAENGIVARAWRDGGGRESPSKCPVESISDIYNIQRLKQPLGLTITVKYVLNGSHVDRTKHIVEVFHSKEFGIAAAFFND

>AdPCASPt1B

MTEEKFIRDLPDAVKKELAFILDNQRPNWKSLMETIGYGASSITQVRQEDQGHYVCRVANRFGFCAFSNWTRVSVSGEQAEDLQCFDFPIITYQSGPVVTTASGSILTLYCDAVGRPAPSFQWYKSNAVIVGATVRELTIHNATAEDGAVYFCKVYNSAGEISSEPIEVKVTDVPTNNMRVRGVSVSGPSENERVANKVALLMGNKDYQYSQQLGKLFHPINDVCDLTGRLLDMNGFKVVSLVNLTLAEMREALRQFCNLLVEETYALFYFAGHGFERHGHSYLMPVDATDKYHPHENMASSEVLKAMQNTKAKLNVVLLDCCRSDCPQSCVFECPEERNGFFIKHLLKNMTNEDINKSIEEVLLGVSR

>AdPCASPt1C

MATVDTVEIERVPTPLRCLKMNVLEELSRKLNPRHNTKDFRYLAGLMNYTYEMVKNLEREKNPTAYLVSEWAMCHASGGEPKTVGDLLELLKEMRRDDAVEILKPFEFTEVSPKPLNNLNNGLNKSHDLSGVRPVGLLFGEYASGDLNEKHPAQENEYPCSNSLSSSRIFNDYIGDDERPHSYQDYLNYVGSPLRSQIPPYHFQNGYSHGEMIRTVQPSHIQQRFTREVPSHPGSRPYQEVLANIQHTVFHNFGSPLASEPRPPVRSSNLQHGSLNDTMNGAIQPPRVQPRFIPEIPSRPVVSDKVALVIGNQKYVNKRLQGLVYSEKDAYDMAHALSELEFKVVSLVNLTLAEMRIAVLMFCRLLAKGCYGVLYYAGHGYEDGGKNYLLPVDSDLKYDRQDSLCAQEILETMQTCDTLLNVLIIDACRLRMPNNSGSVENGVKRGRLGNNIFAYSCSSQHEAYEEPGQTNGLYALHLLRHIRRDERIEFILMDVARDVSMASNRNLIQRPCHESDAVFDCRLCDKIAPSNLSGEFMETMKLWMQAHIKAELAQISGDFLAENGGSLQQRVLLSRLQKQEDPIEVTFKMMYTIDEEVIDWEVQVQLECPLVSSVFVKWDWWITCGRLPAQKSTQV

>EpPCASPt1A

MPVDTYTQNDVNQFDRLGMQRGKKTAEALLQNLGQKCITVKKLIVYLEAIGNNEALEQKKPDERVEITEQPSSSPVMAGERVELVCRATGFPPPEYQWLRNGEEIRNACEKKLIINNTQAKDSGRYSKKTSNRLSAEKSNCVEIQKKDRVVGCQPQKKSQHPISAVVPVGQQFKKTCQVENPKDCDYQWFKKKNKLRSETTNEIFFDSFNFEHEGNYNCRAYNTKGDTLTRLATLQAWAPEXQCPNKGIATDKIALVIGNRDYINLPPNESPLVHTCGDAQRVAAILRKNLMNFKVFSLVNLTYNEMKKALSIYYELLDRGVYGLLYFAGHGFEDSGQNYMIPIDAXTDWTPDKAXCVQKILQEMHNRGTALNVFLLDICRKLTMTGNGYKREIYPYPPDAQSIYGFATCPGAEAYERKADANGLYMTHLLKNITKEATVEDMLKKVAKDVEDEVANDPDVWRMRPQFYSKAVKNFSLRDPTVREDQESKIRNQKWLEIHKLPQKHIFNVAHVGITVEIRFEHTNWLSNVILVCLRVLDLGEATMCDASLILSALPKGLQECHFHPSTANKMRHVVPPQAESSTGVVARAASWASQEEQSHAPVPVERISEIYNIQRLQKNLSLTIELKYILRGSQEEQTKYEIKTFNLKEFGIVNAFAKANPTDDFQDYSLY

>EpPCASPt1C

MYSSKVTVQTLISNLKPNVLDELGMYLNPRMPLKDFRTLAGKMGYTFMRVRNFERESNPTVSLLEDWWTSFGKKGESKTVTDLIRYLEEMRRDDAVDLLKPYEYTERAVQLPRCQHQKPVPFGTYSPDDLQQNRCPEENELLFNPDAPEAVVSGVQNKIENFIHPSSMSESRAQGRHPATVDQNSPLPRSEVPRPMEPPIVSDKVALVIGNKDYQCERLRGLFYPERDAQEIAKALSELGFKVVSLVNLTLSEMRIAVLSFCRLLGKCVYAVLYYGGHGYEDSGKNYLLPIDGDLMYKREDSLCAQEILQTMQDCDTALNLLIIDSCRIRLPDRGTRPTFIKRGAKGNNIFAYSCCSEQEAHEERDQENGLYAQHLLRHIHRNVRIENILMDVARDVSFSKNHYINQRPCHESDAYLDCRLTDPICPXALPNEFRERVALWEKAHMLPKPFVVLETDHIYLMAEFRANFSNVMEVMLIASNRTPFPLTRFVMDLSVPAPVRSEVGMLSGDVLEPGLELKQVFKLSALQKLPDKLMTNLNLFYDFKGRSVELRPIINLGCPLVSSVFTEWDWWVTCGRAPPTKSTQV

>MlPCASP

MLNKIGNQEAVSSLLDIISDNKASTPVKITIEGNHELTAGQTLRLKVNAPSSYTDIQWYFEKTCLPAEKSKDLLLEEVSIEDKGRYKVRLESNSGDVKWAFVDVNILQEIEAPAEELSFLLHPKDYLLKESSSEAHFFVDVRPHDAQLQWYCEGVLMHDKTKPYLYVTGLDQTAHRRQYKCTATHEGRTISSDTALVRWSPEKPKDFTGTSFVETATDKVALIIGNQSYWESTFGDIVHAEDDARDISAALKSMNFKVPVDAPSNVRIVHCIKAQYVLHRMQFRETKLNLLLLDMCREFNNVGGIPGDIQPVKSRGNTAIGYACGPGSRAYGSENDKNGIYVKYLKDHVTRNITVTNLLDCVNKAIGENEPTGYQRPHYQSSIVKNISLNDPIDTTNHTAEFHHRSILWSMANTVDKEEMSVTLLKTELQLEFSAAFSNVLQFSVSPHVDSVRLDPESQANGLLLQKLSQNTFNIRNVQKLVGSVIELSIISGCERGTVRIERPLFAKITANRVNFGNVLDVNLAGSHRPGISQAPQHQLADRSCKNPQNKSCETVDEKANRKLKEIEEFDKKTEEEKSDCIRGQTTDTPVESEKESDTNSLLPPLVKNFDDRIVYPFVGIYLSLKSNGENTEQLVRLVENESRESYRTEQMSRVEMSTTVLNRESSQPWIEVVLKERDYSKSTRNMLEGKHWHGSKVLFSCHVISLLNMLQMLTINHEISEMWVTDHDKFRQVVPDEQLVIDVSPYSLPVA

>TaPCASPt2

MYIWLMKRLDIKVTIILSSFKDDHYDYDSLASSNSEENVGFPEAIPHPAESGRHYHQKLPQFADIPRKVAEDEYGYYNPSLHMATAKYALILCNQNYLYNPQKLNHCLHDALVMSKQLTELDFKVMTLVDVTLEEAEAALAMMNRLIRKDSYVFVYLVGHGLNINEKRYFTPIDAKPEILDSRQHLAIPDIIHLIAKNDPKWIVFLCDTCGSSRVRDHESDVGHYEERLPDIGQNIFLAYSSGFKAYERGFDTNGLYVKHLKNYLTQMDMTIDEIFEKTKEDVINDYESQEPYHVSNMTDIELSLCDDVVQTAEKTPSDIFWDQLHGYQIEPRISIRDPKLEIEAALAYDEFYDALLTLRVKYLENVDYCDVHVVGFHTNIESDETIVKLERVKSCIIDKQQGFVPNLVINATGFLNLRKIRGTGGFIEVVIYTGKCGGRRIRQFLKMSIPPICTIRNWTNFVEFQEVKETIWNAFG

***Paracaspase sequences from RNAseq of coral bleaching (Pinzón et al., 2015)***

>comp264554_c0_seq1

ENMATNKTNVVPTLMSGLSMDVLEKLGMKLNPRHPLKDFRYLAGKMNYTYESVRNFERQKNPTAFLLSEWWMSNAEKGEQKTVTDLIKYLQEMKRDDAVELLRPVEFTEMPLHPPREDVHQTHRSHDRPLHVPPVGNLFGAYSTGDLKETRPPQENEYLYNNNLVPDREIYFEGPDGRPRPYQEIVAFNAQHTMLSDYGSPLALEPRPQVRHPAMDQQNGSDHGEANVVLQPTEHRQPSVIPDSPARPVVSDKVALVIGNQQYECDKLKGLFYSEKDAYDVAQALYTLGFKVVALVNLTLSEMRIAVLSFCRLLGKGVYGVLYYAGHGYEDGGENFLLPVDANLKYERQDSLRAQEILETMQACDTMLNLLIIDSCRIRLPNKSGTVHYCKRGPKGNNIFAYSCCSQQEAYEEPEQPNGLYALHLLKHIGRNERIELILMDVARDVSNANRNRNLIQRPCHESDSVVDCRLTDPIEPTALTDEFVERMKLWTQAHCLPDSIPSINKDGVIISFECRPVFSNVMDLKIAALNANPVPLHQVVMELEVPHPVKAKVRLLSGEELLENGGMLQQVVQLSHLQKLLDPLVVTFKIYYLMGNDSIDWQAPVRLGCPLVSSVFVQWDWWITCGKLPGVKCTQV

>comp267047_c0_seq1

MATEITKAGAKAAMDLSLSGAGFSVSLKIDNVWLAGAVSIGALSLGAFYLVCKGPSENAIKRALERKIFGTGDPEVTNVTDGHSILVELQCHTETSLLLFLEDVETKTVKFRLEEEFKKIGFKEELVVSISNAEKVNEHVRQIRERFKAGQQEVNQNEDWKQKYEELAKATNKIYEIQAPVLHLPETQTITPSTKEPIKRLQELATLLNDLTQRERCVNDSSKRGGLEVLELKDLLEEVKNLKETLRREIAISKGLRRTIEKLQEDNKALAKTTKHERVEESNVMTSDIPELREKVHELEKENELLQQKLTKKDKELERFQGQDTDDATTQARPRASSATSLGASSGYQSEEEPDESNLEIIQMPQSQAIQEGKKLVLSCRIRGLPDVRYRWVKDDVEIPGVNRSDLVLEPVEMQDFGRYFCRVWDKSGSVTSDTADIDVFPATQMRFRGLHEMDQATKQVVSDLLSKKRLPGLATWKQVARRYAMRETEISLLEIEKSPASAMLDRLASLAPNLTVYYLCKTFKEPGLRRQDVVNILSKQIVVSIE

>comp268876_c0_seq1

MDRRSINDLSEDIKNRIAEVLDKGDQLPNWRLLIRDVIRQYMPLYDEAFVTKYFAMETLLPGGSPTLKLLNDLGERKITVGVLINWISSLNQMRPNLQLQAVLNLLTGSPIITQDVESEVHGTHGQHVRIHCEASGQQPLHFQWFKKRVKLHGQTGNTLTLQNVWERDEGYYICRVADSSGCTFTSWAKVIINEDYTAYGSSQFDLPVITSQPDHRVPVHVLQGSQLRLYCDGVGRPAPSFQWYHNNTPIQEATNRVFTRSAASTEDQGLYFCKVYNSAGETLSQSTQVIVARHGGDLPVRRGGSGPTDSDSSDSERVANKVALLIGNKDYHFQQLGRLFHPINDACDLTGRLVNMGFNVVSLVNLTLAEMREALVEFCKLLVKDTYAVFYFAGHGFERGGRSYLMPIDATDSYLRNENLASAEVLSAMQATEAKLNVVLLDCCRTEPDHEVHGSPLLGGDLQDIKEPNIVVAFGCCPQSSVFECEQEKNGFFAKHLLKNITDEHRNKSIEEVLLEVSRGIHEENLKDPYTDQKQVVNRITTLVKPMSLWSPVNSSSMNPSAVEATARWQTAHEIPNVPVTVFQDGRVRIELNFNAETSNVLIVNARALGEEHLDLTVMFDMGDVVSGCSIERGDMSGKKCGPSEATLKVKDLQRLTEPLVFQLKFMYTLNSDRQQKLVSYRMEEKPLYAKLVTHW

>comp268876_c0_seq2

MDRRSINDLSEDIKNRIAEVLDKGDQLPNWRLLIRDVIRQYMPLYDEAFVTKYFAMETLLPGGSPTLKLLNDLGERKITVGVLINWISSLNQMRPNLQLQAVLNLLTGSPIITQDVESEVHGTHGQHVRIHCEASGQQPLHFQWFKKRVKLHGQTGNTLTLQNVWERDEGYYICRVADSSGCTFTSWAKVIINEDYTAYGSSQFDLPVITSQPDHRVPVHVLQGSQLRLYCDGVGRPAPSFQWYHNNTPIQEATNRVFTRSAASTEDQGLYFCKVYNSAGETLSQSTQVIVARHGGDLPVRRGGSGPTDSDSSDSERVANKVALLIGNKDYHFQQLGRLFHPINDACDLTGRLVNMGFNVVSLVNLTLAEMREALVEFCKLLVKDTYAVFYFAGHGFERGGRSYLMPIDATDSYLRNENLASAEVLSAMQATEAKLNVVLLDCCRTEPDHEVHGSPLLGGDLQDIKEPNIVVAFGCCPQSSVFECEQEKNGFFAKHLLKNITDEHRNKSIEEVLLEVSRGIHEENLKDPYTDQKQVVNRITTLVKPMSLWSPVNSSSMNPSAVEATARWQTAHEIPNVPVTVFQDGRVRIELNFNAETSNVLIVNARALGEEHLDLTVMFDMGDVVSGCSIERGDMSGKKCGPSEATLKVKDLQRLTEPLVFQLKFMYTLNSDRQQKLVSYRMEEKPLYAKLVTHW

***Bcl10 sequences***

>HsBcl10

MEPTAPSLTEEDLTEVKKDALENLRVYLCEKIIAERHFDHLRAKKILSREDTEEISCRTSSRKRAGKLLDYLQENPKGLDTLVESIRREKTQNFLIQKITDEVLKLRNIKLEHLKGLKCSSCEPFPDGATNNLSRSNSDESNFSEKLRASTVMYHPEGESSTTPFFSTNSSLNLPVLEVGRTENTIFSSTTLPRPGDPGAPPLPPDLQLEEEGTCANSSEMFLPLRSRTVSRQ

>MmBcl10

MEAPAPSLTEEDLTEVKKDALENLRVYLCEKIIAERHFDHLRAKKILSREDTEEISCRTSSRKRAGKLLDYLQENPRGLDTLVESIRREKTQSFLIQKITDEVLKLRNIKLEHLKGLKCSSCEPFAAGATNNLSRCNSDESNLSEKQRASTVMYHPEGESSTAPFFSMASSLNLPVLEVGRTENSSFSSATLPRPGDPGAPPLPPDLRLEEGGSCGNSSEMFLPLRSRALSRQ

>GgBcl10

MEVSGPASSAVGRPLTEDEMADVKKEALERMRPYLCDKIIAERHFDYPRSKKILTREDTEEISSRSSSRKKTGKLLDYLAENPKGLDALVESIRRERTQNFLLQKITDIVLKVKNEKLEALKGLSCSTCMTSLYGGTNNLSRSYSDESNFFDKTKDKESTQMHNPEEDYSTAAFMSAVSLHSMNLPVAEMGNAESAVFSVTLPGPGDSGAPPLPPELQSEQQEPCTSSSDNCFLPLRSRSLHPQ

>AcBcl10

MAGSGVALPERPLTDDEMAEVKKEVLEWLRDYLCDKIIADRHFDYLRSRKILSREDTEEISCRPSSRKKAGKLLDYLAEHPKGLDALIGSIRREGTQNFLLEKITDAVLKAKNEKLKSLKGLSCSSCMVAVYEQTNNLSRSQSNDSNVFESRKDRETAYLPQGEYNTAAFVSAASLCSMNLPITEVGNTENSVFSATLPGPGDSGAPPLPPQMQNEQEETCPTSSDNPFLPLRSRSLLPQ

>PbBcl10

VLERLRRYLCDKIIAERHFDYLRSKKILSREDTEEISCRTSSRKKAGKLLDYLAEHPKGLDALIESIKCEKTQNFLLEKITDAVLKAKNEKLESLKGTSCNNCMASVCEQPNNLSRSQSNESNLFEKQRDRENTYLHSEEYSTAAFASASSLCSMNFPITEVGNMENSVFSTLPGPGDPGAPPIPTELQNEQEEISTSSENPFLPLRSRSLLP

>XtBcl10

MNKLTDDEMAEVKKDAIESLRPYLCEKIIAERHFDYLRSKKILNKDDAEEILCQTTSRRKAGDLLDRLAKNPKGLDALIESIRLQETQDFLIEKIIDEVLKIKNKKLESSRGYSLSTYEPTLNGSSREYLRQYTFEDKLLVPETESTVLYHPEGESSLPILLNDSQMLVSKSLSEKAKNSSQSGSICSKLPKPGEPGAPPLPTALPCENDNVLANSPIDNQFLPLRSSSFSEA

>DrBcl10

MDVTHLTEDEMADIKKEAIERLRPYLVDKIIADRHFDYLRSKKILTREDTEEISCRTTRGKRTSKLLDILAENPRGLDMLIESIKWGRTLNFIIARITDEVQRVKNERLEALKAGSNSAYPTTKGATNDFSKMDSDYDKYSTMCLHPEGEASSSSSVATGSFNLRYSSTRGNEALMVSGGSGTMNVSSTSSILPKPGDPGAPALPEEMENDEQDMDGGVCKSAGSSGDANFQPLRSRSLSP

>TrBcl10

MDVPQLTEDEMAEIKKDVLTRLRHYLCDKIRAERHLDFLRSRRILTRDDAEEISCRTTQTKRTAMMLDMLAENPRGLDALTDSIREMRSQNFIITKITDEVQKAKNEKIETLKAAASSSPSNNATSSPCITSHLSTMFSHDSTLLFHPDGEQSTSSSALLTAASLQGTNASGVCPDGINVASSTASSNLPRPGDPGAPPLPDEFPDQAPSNIDAASPGCNSPGGDSNFQPLRSRSLTPISQRNII

>CmBcl10

GYVFINANRISVGTLDDGVFVQFLWMFPYIFLVKMSLYHLLKKRTVFQVGTGNLWETWVYIFMTSFFFLTQALEKLRPHLCGKITADRHFDYLRAKKILSREDTEEISNQPTNTRRAGKLLDYIAENPKGLDTLIDSIIREKTQIFLIPKITDEVQRCKNDRIMQKGSSILSSYIMSKDESSTNNLSRLSSFEPSIQKRVDANILFHPEGEVSPGASLLYSFPSLSLKSGSTLEKATTMSNMETMPATLSLPRPGEPGAPPLPDELQAESEGTCSNSSDEFLPLRSRSP

>PmBcl10

NNNGSSSSKTTMPLGEEVEQDIKREVLTNLLDYLSERIQADRHFAFLRARKVLSRSDTEEISYQSTRRRQAERLIEFLELHPFGLDGLLSSLQHEGTQKHIVDRLNN

>CiBcl10

MCEADLDEDVLYELQKEVLKSQRHYLCDHLQPQRLFPWLQSQNSLTMDDCEHMQSLPTTKRTVDKLIDILIKSGPGCLIKLQRAIQRSSKTQVFIADRLKKKYEEYIKQHIEQENLRESEFTTQRTPPPPYTTFPHIHAPHMELRLPDASAFAPSAKFSTSKVGTQFRGSDRLSTSVEVSNPDTPHVKPILFSPTDRVPVTDEWPSKSPSSIPSSNYEDFIRDRISIFGDGSYTAAPNNRDSSLSGKVENREENGLPDHISLQARKIASYSSSPSNTKVFVRDNGREYTSQTSQVLSNLFNEQSVEMEKHQANMRFSSNGRSFTRQSRNPDEHDVSLSYTRSPTHNVPSRIEPVKTSIAADPVVANTNESSSVDVTKENDVSPLLKVTMPKRSFNSDKSIYNATSPTSDNNYLMLHFPNSDSESFEITDDDE

>SkBcl10

MQVAKPEPSAPVSWPTFENGEDYYHYLKCQVLEEQREFLCKHLRHDRYFAYLRSQRVLDADMCEEIELPRKHAAQVNKFVDCLINQGGPHAFDHFIESLKKRGTEGEWFIIRQLVKGFEQVKASSKAVLLPPKDDDKDDEDAHPPPIPGSPGGPILPILSLTNDTGTVTHSSFVCSHGAVSPSYGMSHSPGELVQSSSLYTKQNPRVMVFGDSK

>CgBcl10

MNGNKIKEEEILHEVRLNILEEQRHYLCKHLNPQDHFAYLRSKHLLTREDEELIKSEVTSKRRAERFLDILVQKGPNCYKSLVDALLKNGTQLFLAEKLNKEYERKKINLHNLLTGSEPMDPRGDIGVDPAALPRPTVGSIENQNIYKSHTDSASSSLTTYKSSYISSNDSNA

>LgBcl10

MAELKARGSPIDYNVQVSNERILAAIKNEIIQEKRSELLVSLNPDRHVDYLRSKCLLSEEDIEEIRHLTTRKKRASLFLDTILKNGPKAYDKLCESLLQERTQLFLLEMLNTEFERRKNSYISLIQPEEKEDSSYLQISNDHFSLPKPSSRRLPVIEVDDPE

>ObBcl10

MNEEEILKGIKKEILQSDKDYISQHLKAANHYSFLQEKGVLTKENCDVIEKEASDKEKADKLLEIIISKGPSSFDYLCDALQHEDTQQFLLERLKQKFDMRKHFLLDVCAAKENKPRKVPTSLPIASIDLTSTPCVDSQPSGNSQN

>LaBcl10

MRTDSRQVNEMSKFDPSVSLDNLLYHVKLQILEESRPLLCKNLQPKRHYAYLRSVNILSEDDCAEINHEVTPSSQAGKFLDILQKRGPTVYYHLCQALLKEGSQLFLLRHLNEEFERRRALVIAENPHLVPASLASEHS

>CtBcl10

MTMLSEQRKQILTEKLVKLIECIKLNGLWAHLRSHKIVTKQDEARIKLNKTSHEQVGELLDHLAKGTDRDFEAFCDCLKENNQEHVVAEFLRYSAPSPSNQDVREQLVGRYSLHLQEGETVTTSQSDFYRWIQERLGSGMFRDCRQPPQPNLPADSASIFSAELLAIYQVLTLQELT

>NvBcl10

MPCETAVKNQHAPRLSGSYYEGISTMRDRNMATSVLQDNLYWELKIEVLQELRDDLVKNLYPDKFFAYLRAKRVLDKDDCEDIGAERTRRKQAEKFLDILESKGPDAFMEFCEFLRQNKTQLFLLRKIVERFNEKRELLDQMPFPTELPATTPVSSRPHKSKPDSPSSSSLWEHNTVTGARMTTYLPAPGDPGAPEPPEPDNPPASPPMASLSQDRHSASGNAVTHPTSQENSYTGPPPPPYTSLPPDDPPPPYDEPR

>ApiBcl10

MAXAYISDDDVYWNXKIEVLHEMRDELVXNLTPEKFFAYFRSRRVFDADDCDMIEAERLRRKKAERFLDILESKGSNGFDEFCNFIQNNKTQVWLLKKILDLFERKKRDKLSAEMPQEVIDEDKDXPVTVVMTTSISTPSLNIPLSYQSSDXMLPGPSTPPDELPSRTTEASSLNTDCNCTTTTTLTGNXVSQATSPEENYPGPXLPSYSSEPPCSPPPPYTE

>HmBcl10

MSDRENEVLNFYKEWKNEVLEDLRDELVKDLFPHKHFSFLRVQKIFDEEDEEEIKYEKTRKQQAECFINKISKKGPAAFDNFCESLLNTAGSQHLLEKLLEAFEKKTQQINEQGHIRNSFYRYKIDYIPEPGQIGGPPLPNETNLEPPPPYYE

***CARD-CC sequences***

>HsCARD9

MSDYENDDECWSVLEGFRVTLTSVIDPSRITPYLRQCKVLNPDDEEQVLSDPNLVIRKRKVGVLLDILQRTGHKGYVAFLESLELYYPQLYKKVTGKEPARVFSMIIDASGESGLTQLLMTEVMKLQKKVQDLTALLSSKDDFIKELRVKDSLLRKHQERVQRLKEECEAGSRELKRCKEENYDLAMRLAHQSEEKGAALMRNRDLQLEIDQLKHSLMKAEDDCKVERKHTLKLRHAMEQRPSQELLWELQQEKALLQARVQELEASVQEGKLDRSSPYIQVLEEDWRQALRDHQEQANTIFSLRKDLRQGEARRLRCMEEKEMFELQCLALRKDSKMYKDRIEAILLQMEEVAIERDQAIATREELHAQHARGLQEKDALRKQVRELGEKADELQLQVFQCEAQLLAVEGRLRRQQLETLVLSSDLEDGSPRRSQELSLPQDLEDTQLSDKGCLAGGGSPKQPFAALHQEQVLRNPHDAGLSSGEPPEKERRRLKESFENYRRKRALRKMQKGWRQGEEDRENTTGSDNTDTEGS

>GgCARD9

MCSDQEKRSEGFCLHKPRKDEAAHCYFSLTTMLEEDNDETCWNSLENFRVKLISVIDPSRITPYLRQCQVINHDDEEQVLNDPSLVMRKRKAGVLLDILQRTGRKGFEAFMESLELYYPQLYKKITGKEPSRVFSLIIDTAGESGLSQLLMNEITKLQRTVQEERQKAQELTVWLHTKENMIREMWVRDSLLRKHQERVQKMREERDSLSKELRKCKDENYNLAMSYARQSEEKSSALMKNRDLLLEIDSLKHSLMKAEDDCKLERKHSMKLKHAIEQRPSHEVMWEIQQEKELLLAKNQELENTLQVAREQNLETSLSHETVQNDCSQVLERQDLLNTLYHLRKELRQAEVLRDKYAEEKEILELQCTSLRKDSQMYKKRMEAVLEQMEEVASERDQALLTREQFYTQYSKNLVERDTYRKQIRELGERCDELQLQLFQKEGQLLATEAKLKRLQLELPALTSDLDDTSSRDSQDLTLHGHLDEDSHLTKKDCCKGQTQQFSMQESNLTAESPTFEECSSAHEELSEKERRRMKDCFERYRRKRALRRAPAGRRPEADWEPSTGSDNTDTEGS

>XtCARD9

MSEEEDEVCWTKLERYRVKLITVIDPNRITPYLRQCRVLNSDDEEQVFNDPNLVIRKRKVGVLLDILQRTGCKGYVAFLESLELYYPHLYKKITGSEPTRVFSMIIDTAGESSLTQLLMNEMLKLQQIIQDERQKYKELNKELLDKEDVIRQTQVKDNELKKHQERVKKMKEESDKLITERNKLKDENYDLAMRYARQSEEKNAALMRNRDLQLEIDRLKHNLMKAEDDCKLERKQTRKLKDAIEKRPSQEVILELQRENDLLKARIQELDNPMQNVPKGEASEKTRMYIQVLEDDRRQALEQHQDLVNSVYTLKMSLRLAEDHRDKYKEEKELLELQCTTLKKDSKLYKERIEAILQQMEEVCAERDQAMTTREQFHTQYSKSLINMDSYRRQLREMGEKCDDLQIQLFRMERETLAMKTDLNKLKSPALTSDFEELSPQESHNSPDNKHPETAQQEQKNNPNSKNVAEAAQAQNSSPVNGFGIRRKVNNSFEDIRRKRVLKTRRNSEEYYTTESDAEGTP

>DrCARD9

MSDGPGVFEVEEDDECWARLEDYRMLLIKTIEPSRITPYLRQCKVLSSEDEEQIYNDPSLVIRRRKVGVLLDILQRTGLKGYEAFLESLELDYPDVYRKITGKEPARVFSVLIDTAGECGLTQFLMSEVSRLQKLAQDERRARLEVSAQVKEQHETIRQLQLCENELHKQQERVHRIREERERMCEEARTLKDENYRLMHDLTRLSEEKNCALMCNRDLQLEIEKLKHSLMNAESDSKIQRKKTVNLKNAMEQRPSPEIIWKMQRQNDLLKARIQELESASKVQTPEQEKPDSQTLEDFKQQSQAQYQELVNDLYNLRRDLHDAEKLRDKYREEKDELELKCLMLKKDSKMYGDRMEDILKQLEEVIKERDKAICTREEYHLENSKNLQDKDQYRKQIREMGERYDELQVQLFRTQGEVLALQATLRKQKSPIRVNSGESSLLSSFELKSSEEESRERGEKDMSEESQSQTSGEFNVRFSRRVSGEDISDNCKNNRKCNFHYRRKRALRTKRCTDKDNMQGLLDNTTGSDTDGM

>CmCARD9

MSENEDADVEELWTSLESYRAKLTSLIEPCRITAYLRQCKVINHEDEEQIFNDPNLVIRRRKVGVLLDILQRTGKKGYIAFLESLELYYPELYKRITGQDPTRTFSVILDAGGESSLTQLLMAEVQKQQQVVLQEKKKVNELNRQVVSRDELIKQLQVKANELLKHQQRVRRLKEESDKSCEELKRCKEENYKWAKDFATLSEEKNSVLMKNRDLQLEVERLKHNLMLAEDGYKMERTHTMKLKHAMEQQPRQELIWDVQRENDLLKAHIQELENTVQVDKLDKNKMYIQILEADRRLALEEHQGLVNNIYSLRKELRQAEELRDQYLEEKEVFELQCTTLRKDSKMYKDRIEAILHQMEEVGTERDQALQGREELLGQYSQSLREKDEYRKLLRELGERCDELHIQLFRKEEQLLSLQNRVKQSQSASPTATSDLEESASPCDAQNLGAAAENAAEKKNVKQRMPKIFKDYRRKRAIRLNKRRVHHLSERCENTTGSDNTDTDFSEGDGRSNVQ

>HsCARD10

MPGRAEAGEAEEEAGAGSGSEAEEDALWERIEGVRHRLARALNPAKLTPYLRQCRVIDEQDEEEVLSTYRFPCRVNRTGRLMDILRCRGKRGYEAFLEALEFYYPEHFTLLTGQEPAQRCSMILDEEGPEGLTQFLMTEVRRLREARKSQLQREQQLQARGRVLEEERAGLEQRLRDQQQAQERCQRLREDWEAGSLELLRLKDENYMIAMRLAQLSEEKNSAVLRSRDLQLAVDQLKLKVSRLEEECALLRRARGPPPGAEEKEKEKEKEKEPDNVDLVSELRAENQRLTASLRELQEGLQQEASRPGAPGSERILLDILEHDWREAQDSRQELCQKLHAVQGELQWAEELRDQYLQEMEDLRLKHRTLQKDCDLYKHRMATVLAQLEEIEKERDQAIQSRDRIQLQYSQSLIEKDQYRKQVRGLEAERDELLTTLTSLEGTKALLEVQLQRAQGGTCLKACASSHSLCSNLSSTWSLSEFPSPLGGPEATGEAAVMGGPEPHNSEEATDSEKEINRLSILPFPPSAGSILRRQREEDPAPPKRSFSSMSDITGSVTLKPWSPGLSSSSSSDSVWPLGKPEGLLARGCGLDFLNRSLAIRVSGRSPPGGPEPQDKGPDGLSFYGDRWSGAVVRRVLSGPGSARMEPREQRVEAAGLEGACLEAEAQQRTLLWNQGSTLPSLMDSKACQSFHEALEAWAKGPGAEPFYIRANLTLPERADPHALCVKAQEILRLVDSAYKRRQEWFCTRVDPLTLRDLDRGTVPNYQRAQQLLEVQEKCLPSSRHRGPRSNLKKRALDQLRLVRPKPVGAPAGDSPDQLLLEPCAEPERSLRPYSLVRPLLVSALRPVVLLPECLAPRLIRNLLDLPSSRLDFQVCPAESLSGEELCPSSAPGAPKAQPATPGLGSRIRAIQESVGKKHCLLELGARGVRELVQNEIYPIVIHVEVTEKNVREVRGLLGRPGWRDSELLRQCRGSEQVLWGLPCSWVQVPAHEWGHAEELAKVVRGRILQEQARLVWVECGSSRGCPSSSEA

>GgCARD10

MEAPVGNQGFLGLETTELGFVTQTMNHKARCTEEKLLVNHILPEINFSPEQQLEETDGPRAEQNPSPAPPGEQRPRGGCPRPAALENASLQACSLSEQEEEEDALWEKIESARHQLTRSLNPAKLTPYLRQCRVIDEQDEEEVLNSCRFPCKSNQTGYLMDILRRRGKRGYEAFLESLEFYYPEHYTRLTGREPAQRCSMILDEEGPEGLMQFLLVEVKKMRAQRKEHLLKEHQLQAKNQTLEQEQARLEQRLQELLKVQERCQRLREEWDSNSVELLRLKDENYMMAMRYAQLCEEKNMAVLRSRDLQLVVDQLKCKVTSLEEECSLLRKQASTMPQRETEERGHPDAVSELWAENQRLTASLQELQGMLQTPGEVPVPGSEQILLDILEHDWKEAQDDRQDLCQKLNSLQSELQWAEELRDKYLQEVEDLQLKYRTLQKDCDLYKHRMNTVLLQLEEIEKERDQAIQSRDGVQLQYSQSLIEKDQYRKRVRALEEERDELLSKLSRVEGLNSTLEAQLQRCRGNRSLGKMCSSSYSLCSNLSSTWSLVDKPSDLTSGLTDPLGVSPIIFPEDSAIHSMEGTPDSEKDINRLSTFPFPPCIGSILRRQREDRCMPYKSLSCGSFSSMEDTAGNSFVSIPPGAFSSSSSSIYTRVKSETCLSMLTSHQEISRRSLVVQLTSMGHPSSLSPREKRLGADISILGGNRTGIYVQWVKPGSQVESTGLREGCRLIKLRVPSLKEEVLSLENCTREVAYLSLLHWDEPSSLVFQLDLQGKALAAGFPSSLSSFLPFPGLSPSPGGYQPLREALEEGKKFSGDSFYIRTNLSLLEPSDPYALCVKCREILHVTDTMYKGRLEWYCSRVDPLTMRDLDKGTVPNYSRAHQLLKIQEKVHIPGQQRSHRNNLKKRALDQLRLVKSKQQKSPEQPCQQLWLDPCSDPERSLKPYSLVHPVVVKTPRPVVLSPSCIAPRLIRNLLDLPTSRLDFHVCPAEKLTEGNPSAAHVQEPPVSRNAPQERRESSRVCMIREAMEKNKHCLLELGVQSVRDLIKREIYPIVIHVEVTEKNVRGLRSLLGQPGQRDAEVLKACRGAERALHALPCSWARVEPHAWSHAEELPKVVRGCIFQEQNRPLWVEDGDD

>CmCARD10

MVKSLQILRVLGALLSVSRDRDTEKQRARGHSMNCAGEWTGVEAVQGTGLELESEAERGERLWDEVESKRNVLTRSINPAKLTAYLRQCKVLDEEDEDEVLNSRQLTSRKGRASRLLDMLRCRGSRGYEAFLESLELYYAELYRLLTGEEPTRRCSVLVGESLALAGLTSAPSSEAVLLDLLDQDRREALEDRQELVQRNLSISQCLHETEQQRDQYLREKLGLELRCSQMAAQCELNKRRVTVVCQQLEEVEKERDQAFKARNEAQASYVQSLLARDEHWRQNWALQERLDRLETQMTDRDGKSSDRQGIKCSLPTGHSLDLTDFLDSSLVSVGYRGDGNKKVEKETSFSPNLRRRNCVKRSSSSPIKVSELRAIGDEKCTELCRPAMVASVDMESEGDINRFSILPFPPSQGSLILRNKEDDPIGSLASLSLASTMDILEPSTGVRELAVSRAVYIRVNVSVQGQLDGCSLQVERGEILQVLDIELPLDSDWRCARINPHTKEDLQQVTVPGYHRACRLLQANSSPAVSCQCPCERTQKQGLRSDVVMKPRSLNRIRSVQSIYPPPQDPDTPTPLCTDTPSRPRTTDPLIPAQDTASAHPWTTPFVCCEKNTVALCPYTVVQPISVRRPRPVLLLPPMLAGALTQRLPSPFHPLSPEVVSDDESKRKSGFCLRKIPGQDKSEYISFRAIEEMAAKNKHCVLDMELLSASALVTEGIYPIVLHIKVTEANISQCRNLAPVCEASDHELLQCDRRVEESLRTLPYAHRTVEPGTWTCPQQLVDVVTHGISQEQSKILWMEQSWDQGLLLSGHQHREQPVSHRA

>HsCARD11

MPGGGPEMDDYMETLKDEEDALWENVECNRHMLSRYINPAKLTPYLRQCKVIDEQDEDEVLNAPMLPSKINRAGRLLDILHTKGQRGYVVFLESLEFYYPELYKLVTGKEPTRRFSTIVVEEGHEGLTHFLMNEVIKLQQQMKAKDLQRCELLARLRQLEDEKKQMTLTRVELLTFQERYYKMKEERDSYNDELVKVKDDNYNLAMRYAQLSEEKNMAVMRSRDLQLEIDQLKHRLNKMEEECKLERNQSLKLKNDIENRPKKEQVLELERENEMLKTKNQELQSIIQAGKRSLPDSDKAILDILEHDRKEALEDRQELVNRIYNLQEEARQAEELRDKYLEEKEDLELKCSTLGKDCEMYKHRMNTVMLQLEEVERERDQAFHSRDEAQTQYSQCLIEKDKYRKQIRELEEKNDEMRIEMVRREACIVNLESKLRRLSKDSNNLDQSLPRNLPVTIISQDFGDASPRTNGQEADDSSTSEESPEDSKYFLPYHPPQRRMNLKGIQLQRAKSPISLKRTSDFQAKGHEEEGTDASPSSCGSLPITNSFTKMQPPRSRSSIMSITAEPPGNDSIVRRYKEDAPHRSTVEEDNDSGGFDALDLDDDSHERYSFGPSSIHSSSSSHQSEGLDAYDLEQVNLMFRKFSLERPFRPSVTSVGHVRGPGPSVQHTTLNGDSLTSQLTLLGGNARGSFVHSVKPGSLAEKAGLREGHQLLLLEGCIRGERQSVPLDTCTKEEAHWTIQRCSGPVTLHYKVNHEGYRKLVKDMEDGLITSGDSFYIRLNLNISSQLDACTMSLKCDDVVHVRDTMYQDRHEWLCARVDPFTDHDLDMGTIPSYSRAQQLLLVKLQRLMHRGSREEVDGTHHTLRALRNTLQPEEALSTSDPRVSPRLSRASFLFGQLLQFVSRSENKYKRMNSNERVRIISGSPLGSLARSSLDATKLLTEKQEELDPESELGKNLSLIPYSLVRAFYCERRRPVLFTPTVLAKTLVQRLLNSGGAMEFTICKSDIVTRDEFLRRQKTETIIYSREKNPNAFECIAPANIEAVAAKNKHCLLEAGIGCTRDLIKSNIYPIVLFIRVCEKNIKRFRKLLPRPETEEEFLRVCRLKEKELEALPCLYATVEPDMWGSVEELLRVVKDKIGEEQRKTIWVDEDQL

>GgCARD11

MSTQGGEPEMDDCLETLKDEEEALWENVECNRHMLSRYINPAKLTPYLRQCKVIDEQDEDEVLNSLMLPSKINRAGRLLDILHTKGQRGYVVFLESLEFYYPELYKLVTGKEPTRRFSTIVVEEGHEGLTHFLMNEIIKLQQQVKTKDMQRCDLLAKSRQLEDERKQLKLNKIELMTFQERYNKMKEERNNYNDELVKVKDENYNLAMRYAQLSDEKSMAVMRSRDLQLEIDQLKHRLNKVEEECKLERNQSLKLKNDIENRPKKEQMLELERENEMMKTKIQELQSIIQADKRSLPDSDKAILDILEHDRKEALEDRHELVNKIFNLQEEIRHVEDLRDKYLEEKEDLELKCSTLGKDCEMYKHRMNTVMIQLEEVEKERDQAFRSRDEAQTQYSHCLIEKDKYRKQIRELEERNDELRIEMVRKEACIVNLECKLRRLSKDNNCHDQSLPRNLPITIISQTFGNSSPKANGQEADDSSTSEDSPEDNKFFLPDQARLKRRVNLKGIQINPRAKSPVSMNRTSEFQAVRAQDEDGANASNGRTDTSSSNSVSISNSISSCEVSKIQTLRNRNDSIMSTTPEPPGNDSIVRRCKEDAPPCSMVEEDNDSFGFDALELDDDSHDRHSHGAPSVHSSSSSHQSEGLDAYDLEHVNSIFRKFSLERPFRPSVTSVGRIRSSCHTIQRITLNGDTLNSEITLIGGNDKGSFVSSVVSGSLAEKAGLREGHQLLLLEGCIKGENQSVPLDTCTKEEVHWTIQRCHGPVTLQYKSNHEGYRKLLSELEDGLIASGDSFHIRLNLNISSQLDCCSLSVKCDEIVHILDTMYQGTCGGCDWLCARVDPFTDKDLERGTIPSYSRAQQLLLVKLQRLMHRGSKDETESSHNTLRALRNTLQPEEPAPQSDPKTSPRLSRASFLLGQILQFVSRSENKYKRMNSNERVRIVTGWPSGLARTASEAKKPFPDKLEDLDSKSEINKRLIPYSLVRPIHCERRRPVLFTPTMLAKSLVQKLLNSGGALEFDICKPDIVTKEEFLRRQRMETIIFSREKNLSTYECIVPANIEAVSAKNKHCLLEAGISCTKDLIKAKIYPIVLFIRVSEKNIKRFRKLLPKPEAEDEFLRMCRLKEKELEELPCLYASVEADAWSSIEDLIRTIKDKIGEEQRKTIWIDEDQL

>DrCARD11

MDNGGSMSMLDYGETLWENAEKNRYILCRFINPNKLTSYLRQCKVIDEQDEDEVLNSRLLESKVNRAGRLLDILHTKGERGYVVFLESLELYYPDLYKLVTGKEPTRRCSTIVVEEGQEGLIQFLMNEVMKLQQQSKAKDMQRMDVMTKCRTLEDEHKKLRLTNQELLTFQERYNKMKEERNNYNDELMKVKDDNYQLAMRYAQLSEEKNMAVMRSRDLQLEIDQLKHKLNKVEEECKMERRQSLKLRNDIENRPRKEQVFELERENEVLKIKVQELQSIIQPGPLPDSDKAILDILEHDRQEALEDRQELVDRLCNLHEEVRQAEELRDKYLEEKEDLELKCSTLVKDCEMYKNRMNTIMVQLEEVERERDQAFKSRDDSQHLVSQCLIDKDKYRKQIRELEEKSDELQIEIVRKEAKIVNLECKLRRMAKENGLDQSLPRGITPLIIAQNFGQHDEDSGEDTVDDVEFRLQRRSNLKGRINRPKSPAAGPKSPLFPAQPFTNGDLSEHAAGDSTGFSNASPMNTPAELFSKNIRSRNHSILSTAPEPPDNHSIVRRIKENSDALHKSFLPSDTADTDSGDNADMDDHEPSSINSSSSSHQSEIMDSYDLEQVNNIFRKFSLERPFRPSLTSGPPRNSLRTVQSLSLSGENLLSEITLIGGNDSGIFVNSVQSGSDADLAGVKVGFHLLMLEGNVHGKAESVSLDTSTKEEAHWTLQRCSGQVHLHYKSSYDSYRRLVKDIEDGTVVSGDSFYIRLNLNISSQSDNCSLNVRCDEVLHVLDTMHQDKCEWLCARVDPFTDKDLDKGTIPSYSRAQQLLLVKIQKLMCRGGREDVDTLRVIRSPLQPDEPAPSSDPKCSPRLSRASVFISQILQFVSRVDNKYKRMNSSERVRIVNGGNPSSVSRPGFETLRPEDTSDPESDLNKSLNLIPYSLVTPHQCQRKRPILFTPNILAKTIVQKFLNLGGAMEFTSWKPDIVTKDEFLMKQKIEPIIYSKEKQAGTYECITPENIEAVAFKNKHCLLEADLCCVKDLLRREIFPIVIFIKVCERNIRKIKRLLRVDSEEDFLKMCRFKEKELETLPCLYTTIEPDCWGSVEDLVKIIKDKIFEEQKKTVWVEQDLL

>CmCARD11

MSVQGHEMDNGHLESDNLSVVKDEEEVVWENVECNRHVLSRYINPAKLTPYLRQCKVIDEQDEDEVLNSHMLLSKINRASRLLDILHTKGQRGYVVFLESLEFYYPQLYKLITGKEPTRRFSTIVVEEGHEGLTQFLMNEVIKLQQQAKAKEAQRCDMHIRNKQLEDEKKQLKLKNMEMHTFQERYNKMKEERNNYNDELMKVKDENYNLAMRYAQLSEEKNMAVMRSRDLQLQVDQLRHKLNKVEEECKLERNQSLKLKNDMEQRPRKEQVLELERENETLKTKVQELQSVIQAGKRSLPDSEKAILDILEHDRQEALEDRQELVSKIFNLQEENRQAEELRDKYLEEKEDLELKYSTLVKDCEMYKHRMSTIMFQLEEVEKERDQAFRSRDEAQTQYSQCLIDKDKYRKQIRELEEKNDELQIQIVRKEGKIVALESKLRRLSKDTISADQSLPRNIPATVISQAFCHSCHKSNGQEVESSSTSEESPEDNEFFVQDHRLKRRPNLKVYGIGRPKSPCTLLRQQEGQDEDLSDVSSTKIETPNSIPGCDPLVKMTLRNRFPSVLSTTPEPPCNDSILRRNREEDREIPTRSITEEDDDSFCGYSLDLMQMEMDDDYQDRNSHGPPSIHSSSSSHQSEALDSCELDHFNSMFRKYSLERPFRPSVTSVSQVRNPLRHMLYTTLDSDKLRSEISVVGGNERGLFIQSVQQGSEAEKEGLKEGHQLFLLEGCVRGESQNVPLEMSTKEEAHWTFQRCNGPVTLHHKANLDGYKKLQKDMDEGCITSGDSFYVRVNLNISGQNDSCSMCVKCDEVIHILDTMYQGKCEWLCAQVDPTTDKDLEKGTIPSFCRAQQLLMVKIQRLMCRSCKDETDNQYSTLRGIWSVLQPEEQPPIDPKAPRLSRASIFFGQILQFVSRSDNKYKRMNSSERVRIVTGSPSGPARSTFETLKSSSDKQDEKEKDLDAESDIKKSFSLIPYSLVYPCHTQRKRPVLFSPTPLAKMLVQKLLNSGEAMEFDICKPDVLNKEEYLQKQKSETIVFSRETHLNAFECVTPTNIKAVAAKNKHCLLEAGLNCTRDLLKKDIYPIIIFVKVSEKNIKRLRRTPTKPESSDEELLRMCRSNESELESLPCLYTSVDIDAWSNIKDLIKIIKEKILEEQRKTIWIDQDQL

>HsCARD14

MGELCRRDSALTALDEETLWEMMESHRHRIVRCICPSRLTPYLRQAKVLCQLDEEEVLHSPRLTNSAMRAGHLLDLLKTRGKNGAIAFLESLKFHNPDVYTLVTGLQPDVDFSNFSGLMETSKLTECLAGAIGSLQEELNQEKGQKEVLLRRCQQLQEHLGLAETRAEGLHQLEADHSRMKREVSAHFHEVLRLKDEMLSLSLHYSNALQEKELAASRCRSLQEELYLLKQELQRANMVSSCELELQEQSLRTASDQESGDEELNRLKEENEKLRSLTFSLAEKDILEQSLDEARGSRQELVERIHSLRERAVAAERQREQYWEEKEQTLLQFQKSKMACQLYREKVNALQAQVCELQKERDQAYSARDSAQREISQSLVEKDSLRRQVFELTDQVCELRTQLRQLQAEPPGVLKQEARTREPCPREKQRLVRMHAICPRDDSDCSLVSSTESQLLSDLSATSSRELVDSFRSSSPAPPSQQSLYKRVAEDFGEEPWSFSSCLEIPEGDPGALPGAKAGDPHLDYELLDTADLPQLESSLQPVSPGRLDVSESGVLMRRRPARRILSQVTMLAFQGDALLEQISVIGGNLTGIFIHRVTPGSAADQMALRPGTQIVMVDYEASEPLFKAVLEDTTLEEAVGLLRRVDGFCCLSVKVNTDGYKRLLQDLEAKVATSGDSFYIRVNLAMEGRAKGELQVHCNEVLHVTDTMFQGCGCWHAHRVNSYTMKDTAAHGTIPNYSRAQQQLIALIQDMTQQCTVTRKPSSGGPQKLVRIVSMDKAKASPLRLSFDRGQLDPSRMEGSSTCFWAESCLTLVPYTLVRPHRPARPRPVLLVPRAVGKILSEKLCLLQGFKKCLAEYLSQEEYEAWSQRGDIIQEGEVSGGRCWVTRHAVESLMEKNTHALLDVQLDSVCTLHRMDIFPIVIHVSVNEKMAKKLKKGLQRLGTSEEQLLEAARQEEGDLDRAPCLYSSLAPDGWSDLDGLLSCVRQAIADEQKKVVWTEQSPR

>XtCARD14

MSAQWKADPELMDKEEEELWEMIERHRCKIVQRLSPERLTPYLRQARVLDEMDEEEVLHCAKFSTRAMKMGRLLDLLRTRSKNGAIAFLDSLILTNPRIYTLITGKEASMDPNSFSKLIDSNQLSIYLMQTLSNLHEELMQEKQVKSSLMHHLRKMREKQQQLEEEGESVRSMEHENQRLRREKDAQSQLLSKLKDEQYELSMRYSHALQEKDTVQSKNSELQEQFYAMREELNRLRMDLQVSKSWTTHAQCEEELLCLREENRQLRDRPTQEELGTLEEQQPLDVTLQGNQELLAQLRLSKEKLAASEAVEKLWEKEKDTMVQERHNLQLECDILRKKSEAFHSHVCELKKERDQAYRARDMVQTEISTILAEKDSLRQQVMELTDRNSELLQQILSLQEQLQTQREMRGSLHACEQRDVSTKSKHMRLVRMYAVCPSEEGSRSLSSSISETWTENSCQTSSDLGESFNSYAPPHDDYLTDLTKSASQDLPDLLQTEKTETDSEYEEGLPMFSQMQSSESCSSYNNLAISVPRRRHACRDTSRVTVIAFQGDDLLKQISIIGGNRTGIFIHRVTEGSAADEMSLRPGYQIIAVDFDVLNPSYKVALEGMTSEDAHCILNRVNGFCCLSVKCNKDEYRKLLRDIESGSVSSGDSFYVQVNQSMAGRVGGGLQVTCGEILHITDTMYKGSYQWFAHRVNAYTMKDGESGIIPNYPQAQQQLITTIQHLTWQNGTPRRPQKQIRIVSTDRCNSHLLWTSLDCGICQCEDPPAGSVLGTSCFTLLPYTLVTHLKTVIPRPVLLVPSLLGRILSEKLCSSKDFIKCDTEYLTDAEYSARYLRGDIIGEKEREDVRCCFTRQNVESVAKQNAHCILELGLSCLSALLRVGIYPIILHIPLTEKSTKKLKKPLQWWRNCEDLLMECSQREEAELDSLPCLYHTIDPDSWSDTESLLCSVKEAILDEQKRIVWLEKKTC

>DrCARD14

MAESVLDTPDLKDLQEEELWGLINDNRHAICLGIRPCVLIPYLRQARVLTDLDEDEILNCLTLINRSMRTSHMLDLLRIQGRNGAMALLESLMIHYPTLYTQITGRQPSIEPSRFSGLIKYTELTEYLVHAVSSMQKELQVTQHESSRLRAQCCELEGKLGQAEQNNQEFFQIQREHARLHNHLDGLNRELLKLKDEKCDLYVRYTTALEEKSANVIRNRDLQLEIYQLQCDLRKAQKQTEFQRQHSVKNLSDTQKLKDELSAVRAKLLETETFSPVKQDILAHDLKEIETRRSELAEEVNRINEEKEQLQQEKEELLEEKNSLALEVAKLTVDCEMYKHRSMLFQSQLEDVQAERDKAYLSRDEAQAQIARSLAEKDTLRSQLMELQEKLFTMNACSTQRERREKSRDRKFSCESSPPSSPTLRRQRCDLKTIHPKCFISYDASEFSDEHISSIISNQVEPPCSESLRRRDLHFSSRSDTEDNFSIQDLADDCFTDDDYVVIAKGHCLEESVSSSSISSPMSKRSHPDSVSRVSAPPFLMRSRPQALRITGRILTIFFQGETLLNQIQIIGGNKTGVFVHHVTKESSAHNSGISPGTQILQLKYERKRRAVQMVLEDTTMEEALWALGQVQGPCHITLRPNQDAYENLLQQLKNDEVMSGDSFYVRVNMTLPGDVAGNLSVKCNDIIHITNTYHTNDGFWWGSHVHPCHLEDLKSGALPNYYRAQRLLIRAIEDMTYSRKTHRKGRTAADEKQRVVRIVSTSQQRRNPLWVSVEDDNSKSQDNDGCLPSRCLTLMPFTLVTPRFPPICRPVLLLPTILGRVLHKRLAEQEGYQLCEPEQLMTSEHGMQMQKGEILEECDSKTHLCYTLQGVEKIMKRGTHCVLPFGLDCVCRLHRAEIFPIIVYIISTERSLRRLRHKLRQNSVTESQVLECSCSEEPLLDKLPCLYRSIPPESWHDTATLVDTLKTVVTEEQNKIVWVESDPW

>CmCARD14

MDVVPPGKLVIKDLDEEAIWDLIENHRHIITSKVRPCHITPYLRQCLVITETDEEEILFGPHLKHRCMRTGYLLDLLRSRGKKGGVAFLESLGFYNPEIYTLITGEEPTRECSSMLDEKGNLGMIQFLMNEVMKTHKWLGEEKCQKHQLHEKSQILEEQNQQLKKELESTKAMEANLSRMKMDWHRHHDEMFRLKEENYQILMRYTNTLQEKEMAVTRSRDLQQQVDNLIMENKKLIVKFDVERRMSLKLREEFKPKQDELLHLKEEMYTLRFRIQELQLNPVTIDILEQDRVEALEDRQEMMKEMNTLRLDLDQADQLRNEFLDEKENLLMECTTLKMDCNMYKEKIDSLQSQVSELQKERDEAYGARDEVQVQMSKMIIDKDLCRRQVIELQDTCRELNKEILQIRSNRARQDKKERVIPLREKPKLKRLPAVSFTTSSQENDSDDDRNIQSELCQNVKRYDNVELPSSVRSSFVEPPSKESIYRRLTEDDLDQPYSGRYSIDDDPFPSVDHCSDIDGCIENIPDVIPPLKKSDSSPQGSQTLNGSPPPPEKNWIFPPGIQRLRLLSRVSLVTFNGDSILDQIEIIGGNVTGIFIHNIKPESPALNSGLKTGFQIIMVEYNTQERKRTSLEDASLEEAVWTLKHIKGICSLSIRDNREVYQNLVENIEKKLVTSGDSFYIRANITFNKESGSLGGFTVQCNEILHITDSFFRRNSEWKAFRVNPHTMADMASGTIPNYYRAQKLLIGMIQNMAQQAASTEMVKGRKLSHKISSGQSKLVRIVSADISQRNPLWLSFDSDTINPDKDEDEQLPGNCFTLMPYSLVRPCHPPALRPVILVSTLIGKIVLEKLKDQKDYEKCEPECLRNEDYAEKERRGDIVGLKDIQNSLCSCFTRKAVEAVVAKNVHCLLELSLDCVRQLHRMEIYPIVIVIHMCEKNNKKLKKILHKYRVNEELLLKCAQREEGCLDKLPCLYHSIAPDAWNDLDSLINCVKTAVREEQKKIVWIEKAPL

>PmCARD-CC

SRSQGSPDSDQAMLDILETDRREALEERHDLVNKIFSLREEMRQAEELRDKYLEEKQDLELRCNTLLKDCEMYKHRIGTVLQQLEEVERERDQAFSLRDESQRQYSQSLLDKDKYRKQIRELEEKNYELQIGQVRSDGEVLLLTARLHK

>JlCARD-CC

MRLTSVIDPARLTPYLRQCRVIDGQDEDEVLNSPLCPHSLRHAGVLLDILRSRGTRGHVAFLESLELHYAELYKQLTGNEPTRRFSTIIGEQGSHALVRAFAGVEGQDGLLHFLMAEVMRQQLQLAEGKQQRAELHERASRLETDGRRLALQNRELQAYKDRFGRMKEERNGYNDELIKVKDENYTLAMKYAQLSEEKNMAVIRSRDLQLEVQIDQLKHSLNKAEEEYALERRASLRLQHDMEANPGRERLVELERTNMALSTKVAEMQVLLQFQSRSQGSPDSDQAMLDILETDRREALEERHDLVNKIFSLREEMRQAEELRDKSARHAQYLEEKQDLELRCSTLLKDCEMYKHRIGTVQAFTLRDESQRQYSQSLLDKDKNRKQIRELEEKNYELQIAQVRSDGEVLLLTARLHKATNVADSTRDLVSGTWHRGQGGGCTERHANDDNDRKGKRPHVEKGALREEPLQRLSRRRPFRPSAYSVSVPAAPVRPTLQASLSAVSLRDDVTVIGGNESGVFVRSVRPGSSTERAGLKPGYQLLAVSVGCAGHRKLQALLEGGTVTSGDSFFVLVNASVCGLAEGCTMHAACEDVLHVTDTLYEGRAEWRCSRVDPYTMRDGETGTLPSFLR

>CiCARD-CC

MATFRREVDRLKTENYTVCMRLIDTNEENTKLRESNMKLKNENDKVKAELQKLESCVAMERHQSVRLRNRLQCSPTERDLNDMKREIDELKIKLAQAHSKRNLCDGDVDTRIQMLQQDKKEAIEMYAGALESLAKLREDCAAAEALRNKYLDEKEQAELTCAVLQSDCDMFKMRRDSVWQQLKEVEKEREILMRERNDAQHFAKDCMEEKAKYRAQIRSLEEKYDKLSQDLLQKERELCHLKSSNRPKHKVADTQWTGTDKFISRRSNSFSTCTASSTSSQHEILQDCSDISSECEPFRERTYITKHYEKYLKKIKIFSKITLGNFGEYCQNVLPGIELAADYGPGDADETPFSKPTNEKYKKKWSSGSESEVNGPLIRKDVIRRQAIPRRTNTDITRSATNSNVSLPTQEAAISKTL

>SkCARD-CC

MDEEIDRFRDILEVNRTEIVRTIDPDKLFHVLRACRIFDQEDQEEISSQVNYPTLQKRAGRFLDILATKGPNAFDVFCKSLEGTYPHLYKLLTNKEASEFLPVEPDSTCSTVVHEEFSYLAQKYEKLANEKQQLVTRCNKLVNEAVKMRTENERFKKKAEILQYQKALNKNLKKANRDWEEKLYQATEEKYNAVKRSLDYQEATKLAQEKLREALMTIDDMKRELHKAEGEFKLQRTRTTRLQNELNVKPKTHEIDEYKREIRLLKAELTQIRSTQTVRNSMVLECVDERMAILEKEREEARTELKEMVDALYEARQECHAAELQRDQFLEKTEKLEQEIETLQKDCETYLNRKDELWKQLLEREKERNTAIKERDNALRDWTIKTQERDDIFGRFYKLQEKYDTLEQQLRMIRSNTCYMDSESEPSLGNQTPAQNEDESSFSNNIWHRPRSKRLAHRQISVTESAPENEMPDYDVAFADFRAFRSDDDTSLSCKVHPSSSSRRVLESKKIFSSTTEFDQLSFDSSELSDDNVFPLKIKRRHCNRRRKRFRPRSNSCTNLDVDLEKMLHNSSSDGHSSVYSVQSLRTSHNSNTSGYTSIASNTDYFDVLLSDTTMMDIEITGGNSTGIFIKSVKIESRVACDINVGDQVIKIMGKINGQQKKQSFIDLTLEEAVELMNSCSGAMKLTLKHSQVVYEALRYMDTADVRGDSFYVTANVDSGSWQNSDFTLLINHGNVLHVTDTLYKKGCWYARRVDNDDEGVIPNYNTANEILIKQQSIPPKTPLRRLLSEPEAQTTGCFPGEKVYRVSKFSLRPKARRRIKTDTDTAAAGKKDELCYLRWYFLIIHI

>CgCARD-CC

MSDDEEKDIFEELIERKRYKIVKYINAQVFFDKLRQVKVLSMDDTLEISSKPTRRQQAGYFLNILQTKGDNGVKKFLEILEWEYPHVFKDVTNKDAREPPTDYLKHRESVYAGWLYKLPELAESLKNDYEHKKDLKEKCQENKEVMKKVQDNNLKLEKLLLLEKNELETQLGHCFREKEFAQDKSGKYLMLFLTSNLSHIQLKK

>CtCARD-CC-A

MSTYDEEDEVEERWSEMTPHFQVLSHTLEPPKFMANLMAKKVLSIDDFQQIEGKSSRQDVQIMYLIDMVKRRGRNGYDAFLQVIEFTYPETFTKITKRKPRDHPPPDFICIYNPPSPGVTYDSTPLMKTVIDNLTKDKENLEKKNQQSEEQISRLRTENHELEIKRLEAEKRLREETVSKDMLIQKNNKLEIELRISKTQVAASKDSVAKCEELKRENAELRSSLEAEENFRTENATLKRLVEGLTSEKRTLLEEIEQMEIQANTNNARLTRTEDLVKEEKARNRKLTIRLNREITPPEERTMKRSDLINHICTQKILHSEPTSVVDRYRCLFTSSLQKKREEIPKKHTLLLKITPEAVLALHQCKMDPVVINVVFGEIRNMPDRLQVMQEVPSTLNPAISIPHKMQILHIDDKSESYRERLVGRVVTGITRHIRDIQGSQNT

>CtCARD-CC-B

MSSYKKLDEDEYDVCDEGDREWEKLLPYYHMLSQSLELPIFRSWLISKNVLTPPDFEELEKKFYRSTEKIQHMMDMARRRGQRGYKAFVEVIEYGYPDLYRRILKKEPKDPPEDFRERRRKSTTQPKQSNHAAELLQLMIKCAQSQQMQLTPDSGGKHSENFNKMTAAANRLERENTLLNQEAQRLEDKLTIFEETIGELTDQRNEFQDGMDEAIKKRNELKVENSDLKNELRDNEIKLLENQQKINELKDQVAKNEFKHHSQFETLRRRNSELSLLLKSSENTQSNREQAAANKITELNAKLTEAERTSEQYACDANNLRAEIALLKESIQKLHSRLQDREHDLKTALDEAERKENSWRDELKKRMEMEMQSRTHSVRLKRTEQNVEMLRGQLLNLQLLLREKGDATERRSRPEGSCENTSVKRVSPPSPSDRRAVVFPDFVDVTQENPAYGLLNAIDEHVSSQVEEANAEELLEPTAQSVDTRPKRNLSRQDAMTIASRISTPPDSDRRVVIIFAPDDIRDDLFSSLSQFVGGKFKVCPLEEKTMTKSDVANGVCTLNIIHYEEIEANSRYRCLMRSSLDRTIMGLPYNTFLLLNVSPEAALALHHCNVYPLVIHVMFGAMRDSKIPEKAPRHLNPVPFRILSFDDKAEKLRNKLIGKISTQIAEHLEHLNQRQAP

>HrCARD-CC

MDDRQDWQEFLTESYHSLARVIRPQCFYTYLRSKNVFNLCDQEYVENTYVTTVMKAGCLIDTIMTKGEPGFKAFMEIVEYKYPELFQTVTGRSPRLPPRDTQEPLQQARNVDSSLLSPDLTLIILHIALRNSRVLGSIGPRTSIHIGKNSIEGRAFPYFFIFSSAALAAAKPHSLLVRFTYRSARVPGTVTSHAKHLPALQGISVHPSGRLPSCAEMPDGSLRTGIEALEKSRQMAVRRAPSNSPNLEGSMCLDVRRKYINLGKLRALRIIVKASWAERQETRPRANLSSEYFAPEWRTRITSVTDSLGLLMKCLGPLQESLMEKREAFRELETRILTLKEENEECSQQFLAQIELLKQQIDNINIEKEILEKDLTSCRQSEQNYKKLYEDMRAQRDREQERSIQLRERCDVMGSKVNNLSVEIETERQRSMKRRTTTPNDLDDGDCNDDRSYIGAFKKVEEDLKREREKNKKLKEECQGYMKKYKEVIERVENLEKEMEEQVEKQNSESPEQKIIRNKYSKKECLPRILDREIPNQSKSEQTTNASGNSRNDVKRFATIGASPLVVFAPRDVEKELRNILANSIKPYPIEVIRSEEEMNMCAYHSDILYSTPLGNDGGYCCIRYSSLFEMKETYLVTVPCECVFRMIKNNIIPIICLVKFYDMAGFSELNREEERFEKENIKYLSLKLGGSDWEDGVWWR

>NvCARD-CC

MENEEDSDFNLKIEESRFEIVKRLNLDRTSLLDYLRSKGIFDAGDCDLVLSEKTREHKAGKLLDILETKGNNAVLIFVDALQFQNPDLFEKITGKKANPGSLYNDMTLTLADCRYIPDVEILSGQLKQTISDFHDLAMRYHEVLKENQSLGKHLDQASTDLEAKHRRVGILEKQMIDTEAGVAEAHKNASQLSEAVTVQLRNLQQIICERNTFIIALQMKLLTAEEEAKQMRKVSEFEKQCRELQFVNQKLIAERDESGNQLNELKDWTEALKAKFDLVTDEYKQLQQSFVNAAADNCKLKENVEELKLHLSLNKIATADLKNWNDELEEGIKEYRKQRDFFSESRKEALQERDNAQKERDEAVQKYHDVIQTCDAMVTRQCEHTKHFEEKYDKALNELRPLQRKIAAAQLEIEELKSKLRVHDDREKAERMSCEHLNEVNTIKDRIDVTQLYESVSEASSPQGSPTGKLEGFSWADRRATTAIIDSVRKRVAGTQQTQVKVMSLDRALITKPDDTNPDLLQAGKMVFKSVPSYGTLQCMFGGSLGRNGLGSPAEGTTTQDTGNEHSLDSTSTNSSRNCHEEGASPRSEGSKSSGAENDCKVEEDKPEVSKPKSRPHLYSKSLSTSALLLNGNKVKHAVNDELLEEESSDLYDNDSTDIDNSFHWVRTDECGNPLVGSRSEGELRPEQGALGMTENELHAFYNNLIHKPLNFRQRAWAMKKKSTRRQSEPTIRVLHDNGTNLAKTS

>ApiCARD-CC

MDDEEDYRVVLEKSRFEIVQRLQLDRSSLFDYLRSKGVFDASDCEIVLSEKTREQKASKFLDILQTKGKDGLTYFLDILQLLNPGLYQCITGMKANPCRIWENMRDRFAIGNCSQVPDVELLSGQLKRTIDDLQDLALRNQQLLKENHNLGMKLDQASTDLDLKHRRVEVLEKQKHHSERGVLEAHSNANRLSECVTQQLRVLQQGLFERNTYIIALQMKLLATEEEIKESRKTQDELRANNEMLSAKWHELSRNYDHERRESMKLSAKLKSQKGNLKKEFKQINQKMIDERNESLYKLEDLKDWTEALKARYDLVEQEYQQLQQNFVSAATENSKLKEEIEELRLQFSLSKIRVSDLKGWNADLEDTVKQYRDQRDYFSEARNTALTERDEIHKELEVTISRCKEKLTEREAMITKLTEHGKQFEEKYELACTEIKKLQQRNTTAEQQLEDLRLKRVKANSCGTQTDIIDTGQCEENSEASSPDLNTDAIEHIKGYPWKHRRDVNEIMHSVRKRVTGQHHKAMSLDRSLDMLNVDGSLSQEAKKCLSLQGGNLLFKTPPTYSLLRVMFGLSVGGHLGSLADLSADQEEEEKQEGTYTDTTDSVSPDDAFDKFIDLEYDDVDQNSNQGQFISSENTAEEPKLRRTFTKSFSSPNIKVKTADELRIPRDKSQGNEEEDFHWVTEHELDRAQFAVNGKVSGKKIKSRSPAFRQRAWAIKKKGQRSTSEPAILFPCHQNEHVEISSQETTDR

>HvCARD-CC

MLANDMEEELDYRNLLEKNRVEVIKCLNLDRQFLFSYLRSHSVLDDEDCEIILNAGASRQQKCSKFLDVLSTRGPRAIEYFIQALEIDHEYLYEVITGKKPLNQPLSSNYVVPLKPVLSEKELHEHKREYLLNELTKLAEENRNHAYLYSKTLTEKNVLEKRLNDLKEELIEKEKIINSLEEDIKLKSLNNCSKLYSHHFREMLIENADKSNVIIGLQARLLLLKERNEIISYNNVELVKKNEDLMNQIKDLAIKYDFKRLESKRLSLQINLNCDSLKAASDCKKEIYSLKFKLIQANEEKNYAEKKFEEAQKVISDMKLQLELLQNKSYKSEKKEVSLSHLELEEEISNMKLIHEKQKKKIEGLERKISRLDQEIETHKEEKAFYLTERQQAICDRNRIEEERNELQKHNKVLQDLKEETVQKQIKISSHYEKKNKELSSELDKKKEELALKINEINLLKNINQAKDDEDTEAKANLKNENLHKSIQCLPANTNKNEVNKSNEQVLRQNSEFESVNISESEKNENTENNENNENNVVNTSIATSHNPNQQIFEYDGSHVYKMFKEQKSAAAALLMAVPLFGAATLFSGKEPNNHVTEENNSQQKTNFDKTKQNFSQTL

>EpCARD-CC

MDDEEDYRVVLEKSRFEIVQRLQLDRSSLFDYLRSKGVFDASDCEIVLSEKTREQKASKFLDILQTKGKDGLTYFLDILQLLNPGLYQCITGMKANPCRIWENMRDRFAIGNCSQVPDVELLSGQLKRTIDDLQDLALRNQQLLKENHNLGMKLDQASTDLDLKHRRVEVLEKQKHHSERGVLEAHSNANRLSECVTQQLRVLQQGLFERNTYIIALQMKLLATEEEIKESRKTQDELRANNEMLSAKWHELSRNYDHERRESMKLSAKLKSQKGNLKKEFKQINQKMIDERNESLYKLEDLKDWTEALKARYDLVEQEYQQLQQNFVSAATENSKLKEEIEELRLQFSLSKIRVSDLKGWNADLEDTVKQYRDQRDYFSEARNTALTERDEIHKELEVTISRCKEKLTEREAMITKLTEHGKQFEEKYELACTEIKKLQQRNTTAEQQLEDLRLKRVKANSCGTQTDIIDTGQCEENSEASSPDLNTDAIEHIKGYPWKHRRDVNEIMHSVRKRVTGQHHKAMSLDRSLDMLNVDGSLSQEAKKCLSLQGGNLLFKTPPTYSLLRVMFGLSVGGHLGSLADLSADQEEEEKQEGTYTDTTDSVSPDDAFDKFIDLEYDDVDQNSNQGQFISSENTAEEPKLRRTFTKSFSSPNIKVKTADELRIPRDKSQGNEEEDFHWVTEHELDRAQFAVNGKVSGKKIKSRSPAFRQRAWAIKKKGQRSTSEPAILFPCHQNEHVEISSQETTDR

>AdCARD-CC

MDGENITFDEILDANRVEIVQRLQLDRTFLFDYLRSKKIFDLGDCDLVRAEKTREQQAGKFLDVLITKGEEGYRHFIDAIQLLNPSLYEKITGQKATARSNSHGPDLDIMSNHLKRTMSDLQDLTIRYDEVLREKSVLEKKLSKTSRELWEKNQLIDELEKRYFDTKAMMMESHSSAKKVVEGAAQHQQEQNREMLERTHFIIALQMKLLSTKEEVDILKEKLEESNSEKENLLNRFSQISKNYDNQRRESMKLTEKLEHQKDNIQRAEELKVKVRQLQFSNQKLKQEKDEALREQEELKCWTEALKARYDIVEEDRKQTQESHESTVADYSELRDKADELELRLTICGREIEDLKKRCKDYEQTSNTYREQRDLYEKAWKETAAERDEMRKDREETMCKLTELIHGRDEAIDRQMEYSRQFELQYKKTAEELHQTKERLYQTEREMEDLRKAKLQRNPSDLKNIKSLRIDTEAAPGNQLVEGTQIDENSPAESSQSSEDSFDWRSRRKISDIIDSVKKRVATHRSPVTEQHSGNDSGEGSPQKNLSLDEKLEELMPERKCLSPEAQNCLSLDRNRGRSLFKSAPTYNTLEYMFGPSVSKTTSIYGGFDSNHSAYSSFRSSRSRVSELPGRLEESDTVDDGERKAIERECKIFIKLLEAIKSDATEIVTDIELIQAEKTTESKAGKFLDILTTKGESAFHHFIDALQILNPNLYETLTGESATKGSNPIFSDIQRHITSGVGSIYLDVDILSSYSKRLSEDLQELTLWYDQKIKENNELRRKLEEALSEKKRLQRRITALEKHVSDTEEALVNEAHSSVCKVNESLLKRFEGVRYKERTNQFIIQLQMKLLTAHEESETLRQQMEELKASNDDLMRQLSKVNVDYAFKKKETTRLSQQLKVQSAEMKSCEGMKLKLREAQFQNATFRCELQKKEEQILELNQVKYWSEALKARYDLVLKEKEQALKNQKAVEVKCFSQGDEISSLKMKLNQKERDLEEIKTGYNDVEDSSRIYREERDLYRLTLRDTAQNLEQARRQNDATVQRYKKTLDSKETTIQQQEEHMLQIEKKYEESKEKLASAKLRLKQQEEKNEELSKKISILEVQLQEKGETLKSHSLNQDDSLEDLQSVCETKDYGEAESIQEINFPKDREPKEVVGSIIRRFKGKDQQNTKGNQVEDTKFLKSKSMERLTQIIPEFKDLPLETQKGCSIDSRTMKQSLPYKLLYTIFQSTTGLDSSFPDSAMVQPCETPNVIRQLARRSMSLTDVADFLSFQPQDQDTSNNLQNEEGDDVTRGKFPLENTSDFQENQLDTAQTIRGRTQLLLRRNGERRQRRKTEPSLFTPDFTEMEHL

***Syk/Zap70 homolog sequences***

>HsSyk

MASSGMADSANHLPFFFGNITREEAEDYLVQGGMSDGLYLLRQSRNYLGGFALSVAHGRKAHHYTIERELNGTYAIAGGRTHASPADLCHYHSQESDGLVCLLKKPFNRPQGVQPKTGPFEDLKENLIREYVKQTWNLQGQALEQAIISQKPQLEKLIATTAHEKMPWFHGKISREESEQIVLIGSKTNGKFLIRARDNNGSYALCLLHEGKVLHYRIDKDKTGKLSIPEGKKFDTLWQLVEHYSYKADGLLRVLTVPCQKIGTQGNVNFGGRPQLPGSHPATWSAGGIISRIKSYSFPKPGHRKSSPAQGNRQESTVSFNPYEPELAPWAADKGPQREALPMDTEVYESPYADPEEIRPKEVYLDRKLLTLEDKELGSGNFGTVKKGYYQMKKVVKTVAVKILKNEANDPALKDELLAEANVMQQLDNPYIVRMIGICEAESWMLVMEMAELGPLNKYLQQNRHVKDKNIIELVHQVSMGMKYLEESNFVHRDLAARNVLLVTQHYAKISDFGLSKALRADENYYKAQTHGKWPVKWYAPECINYYKFSSKSDVWSFGVLMWEAFSYGQKPYRGMKGSEVTAMLEKGERMGCPAGCPREMYDLMNLCWTYDVENRPGFAAVELRLRNYYYDVVN

>HsZap70

MPDPAAHLPFFYGSISRAEAEEHLKLAGMADGLFLLRQCLRSLGGYVLSLVHDVRFHHFPIERQLNGTYAIAGGKAHCGPAELCEFYSRDPDGLPCNLRKPCNRPSGLEPQPGVFDCLRDAMVRDYVRQTWKLEGEALEQAIISQAPQVEKLIATTAHERMPWYHSSLTREEAERKLYSGAQTDGKFLLRPRKEQGTYALSLIYGKTVYHYLISQDKAGKYCIPEGTKFDTLWQLVEYLKLKADGLIYCLKEACPNSSASNASGAAAPTLPAHPSTLTHPQRRIDTLNSDGYTPEPARITSPDKPRPMPMDTSVYESPYSDPEELKDKKLFLKRDNLLIADIELGCGNFGSVRQGVYRMRKKQIDVAIKVLKQGTEKADTEEMMREAQIMHQLDNPYIVRLIGVCQAEALMLVMEMAGGGPLHKFLVGKREEIPVSNVAELLHQVSMGMKYLEEKNFVHRDLAARNVLLVNRHYAKISDFGLSKALGADDSYYTARSAGKWPLKWYAPECINFRKFSSRSDVWSYGVTMWEALSYGQKPYKKMKGPEVMAFIEQGKRMECPPECPPELYALMSDCWIYKWEDRPDFLTVEQRMRACYYSLASKVEGPPGSTQKAEAACA

>DrSyk

MVDKVHLLPYFFGNITREESEVYLQQGGSGDGLYLLRQSRSFLGGYALSLSYGRQFYHYTIERELNDTYAIAGGKSHRTPIDVINYHSQESDGLICLLKKPFHRPRGTEPKVGPFEDLKEQLIRQYVEQTWNLKGSALDQAIISQRPQLEKLIATTAHEKMPWFHGKIDREDSELRLLNTSRVNGKFLIRQRENSGRNESYALCLLHNNQVMHYRIDKDRAGKLSIPDGKKFDTLWQLVEHYSYKPDGLLRVLTETCPRPSHHSEGSAENPYKITPNGKDDYSKQGNFLDSAMPMDTQVYESPYADPDELRSTKVNRSDLTLEDGELGSGNFGTVLRGVYQMKKTQKVVAVKILKNDDDNAAVKDEMLREANVMQQLDNPYIVRMIGICEAENLMLVMELAELGPLHKFLQKNKHITVKNLTELVHQVSMGMKYLEEHNFVHRDLAARNVLLVTQHYAKISDFGLSKALTEDENYYKAKGHGKWPLKWYAPECMNYLKFSSKSDVWSFGVLMWEAFSYGQKPYKGMKGNEVIQMIENGQRMSAPPDCPPEMYDLMKKCWTYKPDERPGFSVVEPRLRHYYYDISQ

>DrZap70

MTEPAADLPFFYGSISRSEAEEHLKLAGMGGGLFLLRQCLRSLGGYVLSMIWNLDFYHYSIEKQLNGTYCIAGGKPHCGPAELCEYYSKDPDGLVCTLRKPCLRSPETEIRKGVFDNLRDNMLREYVRHTWKLEGDAMEQAIISQAPQLEKLIATTAHEKMPWFHGKIPRQEGERRLYSGSQPDGKFLVRERDEMGTFALSVTYGKTVYHYQILRDKSGKIAMPEGTKFDTVWQLVEYLKMKPDGLVTVLREPCVNQNNAAPAPANAPRRSRGNGYTPPPMVPKPMGAEGSRPSLPMDHDGFTSPYDDPNELKKKTLFIKRDKLMIDEVELGSGNFGCVKKGVFKMESKQIDVAIKVLKNENEKSVRDEMMREAEIMHQLSDPFIVRMIGLCEAEALMLVMEMAPAGPLNKFLSGKKDQITTENIVMLMHQVSMGMKYLEGRNFVHRDLAARNVLLVNQQYAKISDFGLSKALGADDNYYKARTGGKWPLKWYAPECIHFHKFSSKSDVWSFGITMWEAFSFGGKPYKKMKGPEVITYIEGGSRLDCPAACPEAMYELMKECWTYKHEERLNFAKVEEKMRTFYYSIAKKIPDYLKTENGTPPK

>CmSyk

MSDRVNYLSYFYGSITREEAEDFLKQAGMADGLYLLRQSRNFLGGYALSLSNDKTCYHYTIEKQMSGHYAIAGGKNHNSPVDVCEYHAENSDGLVCLLKKPCNRPHGIQPKVGPFEDLKDQLIREYVQMTWNLEGQALEQAIISQKPQLEKLIATTAHERMPWFHGKKSREDSEACMLRGPRTNGKFLIRDRDSNGSYALCLLHEGKPYHYRIDRDKTGKLSIQDGKKFDTLWQMVDHYCHKSDGLLRVLTEPCSNPDHPVDTFGLMPAPPVPSNHPRKPSSVDRSNSAKNVNPYVSRPQRMPGEAGEGARRDMPMDSNVYESPYADPEELKEKTLYLKQDSLILEEKELGSGNFGTVKKGIYKMRKKDISVAVKVLKSNDPAVKDELMKEAHFMHQLDNPYIVRMIGICERECLMLVMELAEQGQLNKFLQAHKDVITITNVVELVHQVSMGMRYLEEKNYVHRDLAARNVLLVTQHYAKISDFGLSKAINAEENYYKAKTTGKWPLKWYAPESINFSKFSSKSDVWSFGVMMWEAFTFGQKPYKGKKIQEVLPMLENNERLESPARCPPEMYELMKDCWTLNAADRPGFVTVEHRLRDFHYTISKE

>CmZap70

MRDIACCLPFFFGSISRSEADEYLRMGGMSDGLFLLRQCLRSLGGYVISIAYESKIYHYTIERQMNGTLAISGGKPHCGPEILCEYYSRDADGLCCVLRKPCIRPSGVEPKSGVFDSLRDSMLREYVRKTWNLEGEDLEQAIITQAPQLEKLIATTAHEKMPWYHGSISRSNAEQLLYSNNQTDGKFLLRERNTPGTYALSMIYGKTVYHYKIDMDKSGKFSIPEGSKFDTLWQLVEYLKLKPDGVVFTLRETCNSPSHPDSLRPPSLPPNRLSVVALNINADGYTPDPVKGRVPLPMDSNVFGSIYSDPKEMKLYLLRENLMIDELELGSGNFGSVKKGVYKLPKKHIDVAIKVLKNDNEKSLKDELMKEAQFMQQLDHPYIVRLIGVCDAEHLMLVMEMASGGPLNKFLSSKKDQISVDNIVELLFQVSEGMKYLEQKNFVHRDLAARNVLIVHQHYAKISDFGLSKAIGADDNYYKARTAGKWPVKWYAPECINFRKFSSKADVWSFGVTMWEAFSFGQKPYKKMKGPDVVSFIEQGGRLERPNECPEEMHKLMQECWIYKWEERPGFVHVEDKMRDYYYKLTQQLAAADDAADKS

>JlSyk

MDASGEPLPYYFGHVGRDEAEGHLRAAGFVEGLFLLRKSTSTVGGLVLSVVCRGRVHHYEVEPRPYDTGPGLEGRGCGIDGGRTHAGPAELCAHHEHGADGLCCPLAAPCHRPDGTPPRSGYFEDVKDQTIRDYVHKKYHVEGAEADALIARQRPDLEKEVASSLHEKLPWFHGSISRKTSEERLLSQRQVPNGRFLIRQRNTGGSFAISLVFKKKVFHYVVDTDKSTKLSIPEGRKFDALWQMVEHYSMKEDGLMCALGEACICPEYVDLYGNYVDARTHEPTRKNWFSSSFPSALRLKNLIPAITNGFSSPEPPIENPGPVQNNVYYSKPPLPGAAVSDAEGEGVYSDLSDVRCIFLNRNLLQLQHNTELGSGNFGTVQKGTYTLAGKEVQVAVKVLKPVENQEMLKKELMKEAELMHQLDHPFIVKMFGVCEGEALMLVMEMAPLGALNKYLREHRGTMPVENILELMHQVSAGMKYLEERNFVHRDLAARNVLLVNERYAKISDFGLSKPLANDEYYKASNVGRWPIKWYAPECINYRRFTSKGDVWSFGVTMWEAFSFGSKPYPKMKGHEVLVFLESGMRLEKPEHCPDPVYQIMLAAWNLSPDARPMFQEIDMQQESLKNETLNLGQDTLPPKMPSRRSTARP

>CiSyk

MAGQLYDEVYYHGPISRSEATELVKGQVDGSFLLRKSLREPDGYAISVSYQGSVLHYSIQHSTVSGWYSVNKGSEHPSPQALCKYYMQEKGGLKTKLLWPIKVASSPPPQTPPHSPQYQQPLPPLPQVRPGRSGSTVSYEEQEWYHGEITRQEDAVRLSHSYRELNKNGIYLIRFKENGVFVLSVVNDKKVHRYKILQNTSNLKYSINGKPPEFLSLEQLIQFHEEQPPEISHLHCRLTLPCKRSTSASVIPQNCFKHLVSQQQRTNSHNASSKRLSGFASALAAEFKSLLPANGLDVNWSENPHQLNLSENPVSQRPQSSLNGKVGKPVMDRFSRSSLPKDSIGYKSPYMNFSDKSSLAPDLYITTDKLIIRGDIGKGHFGAVKVGECKIMGSFVPCAVKNLTGSDIETNKNDLLKEAKLMQQFDHPYIVRLLAICDAMESSGSLMLVMELAPLGSLKDFLSQHNEKSFPEERLLVLMNQVCDGMAYLSGKNIVHRDLAARNILLVTENFAKISDFGMSRIFLESDNYYKASQPGTWPLRWYAPEALLYYKFTSKGEIWSFGVTLWEVFSYGSRPYSGLKGREILAMLEGGKRLECPCGCSPGVYTIMLQCWAYDPEDRPTFNDLLLQFKVFIG

>SpSYK

MEIHNKLCSLQLGPCIMERTSILNSTNHHHHHNNHHQQQHPVSPTPKINHHYHQQLVQPGRIMPVDPLSRRFYHGRITREEAEARLRNAGCHQGMFLLRESLGREGNYALSLCYEDDVYHYAIERQYDGTVAIKDGKNFAGPIELVSNHMNREDGLLCRLLRPCNLQQGIAARAFSDMDHQQMEGALIDLAKSQGMAPEVITKALHSTQRPQLERALKKIMHQDKPWFHHKLSREEAEQRIVSAGLQEGKFLVRERTEEGSFALSLCYNNFVYHYKIDKDCTGQLSIKEGPKFDSLIQMVDHYQLKKDGLLCCLLVPCHDNNRDTPTRRRHSDRNQRRIASEDGGGVPPTPLRTALSVSVSSPPGIRAPLEYNPSPPTPNRTNSSNNVNDLHLTQMGGHGWDEPEHEGETSIEEFVLVEELPSQRPREYHDDDDKIYGVANEQEKLYDYLAKTKTTKKLDATCLNQGDQIGKGNFGSVLKGTCMANGQLIPVAVKTLKADFGIPNSEPEIVREAELMAGLDHPHIVRMIGICHAAEMMLVLELAELGPLHKYLKKHQEMSTRNVLELMYQVAQGMCYLESRQFVHRDLAARNVLLVDETFAKISDFGMSKALGLDSQYYVAETAGKWPLKWYAPECIYKFKFSSKSDVWSYGVTLWEALSYGKRPYASMRGQELMQFIENGDRLSQPDRCPDDVYSLMRRCWLSEAKDRPGFGDIESVLSDILKRLQAGTLRMRTDRQ

>SkSYK

MLRFRKKTDDPYKFRYFHGRITREEAEKRLKDAGCSTGLFLLREKLLESGSYSLSLCFNSMVYHYSIDKQDDGTVAIEQGKRFNGPIDFVNHHQVHLDGLLSKLLRPCNLPQGMEPEMAEGMSRSALQADIHQAAHDMGIDDLPADQLERTVLKVIHTQKPWFHGTISRDEAEKLITKFGLVDGNFLVRERETAGSFAIGLCYKKTVYHYKVDKNDTTGKLSIQDGQKFDSLFMMVDHYTKRKDGLLTTLVRPVVNPKNKKKQSSTFKQRIWGGANGGASTGTASLASPNVSPRLVPKVPHHSSSGSLDFESGMSPKLTPKAQRKPPSSPHLSHRGDSPTSDTSPNGLAIPDLPPRPLPNPPRARSTAEEEIYGSSVSNAEKIYDRVAKKKKSTVIDPRNLKLMNQLGKGNFGSVVKGNCRVDGQEIPVAVKTLKQQDGFPNNTSEIMKEAGLMTRLDNPYIVRMIGMCQGGEGVMMLVLELAELGPLNKYLKENTMMRLRNIIEIMHQVAMGMQYLEEQQFVHRDLAARNVLLVTETYAKISDFGMSKALGLGSDYYIAESAGKWPLKWYAPECIYRFKFSSKGDVWSYGVTLWEAMSFGKKPYA

>CgSYK

MAARFNKPSASPFFYGRITREEAEYFLRERGSEEGLFLLRESISPLGNYAISICHNNNVHHYSIEKRLDGQFMIAEGKPFPGPLELIEHHKTTIDGFVTKPTKACNRAKFQPAIAFRGMTYTDLENELLKKAEKMKNVRMETALGAQRDHLMVMVAKDLHTQMPWFHGSISRDEADRRLAADGHDDGKFLVRLREDKKTYALSLSHKGETRHYMIEKKDKFHILGGPKFDCIMMMIDHYHNKADGLLCKLAVPCPAPNYDKGKWRKYMDYNCSNLAYGSTDQFSRSSDIQDGPGVPSFPAPQPPVGSSPPVPPSRPTGRRSSNRVRAPLPPLPRGIKDETWEEQDFPPERHRSMHGIDANNLMKIYDTVPRNDEMFSLERRQIKLDQENLGSGQFGSVAKGECTLRNGSVIPVAVKTLKNEDIGAKEEMLKEARFMMNLNHKHIIRMIGICDADCVMLVLELAPKGPLNSFLKNNKDMKQSNIVEIMWQVASGMAYLQQKKYVHRDLAARNVLLVDEHTAKISDFGMSKAMSREQNYYEAQNAGKWPLKWYAPECVYYWKFDSKSDVWSYGVTLWEATSYGDKPYRKMKGQEILRFLVEENQRLSKPPRCPDEVYEIMLECWKYDKNERPTFIQLETKMSYIFNCLLERGE

>ApcSYK

MFKKASKLFSRTDKDKENSSKEVSEAEVLGVYIEPFFYGRITRQEAEDVLKVNGNADGLYLLRESFTPMGNFSLSISHNGEAIHYAILKQIDGKFQISDGVAFSDPVSLIEHYRSSKKGFLTVPKTPCLRIPPQEAIACRGLSYTELHSRMKTAAQKMNANIAKAFGPLREPLLVHVLKTIHKDMPWFHGEISRDEGNKRLEMDGHKDGKFLIRSRADGQSFALTMSHEFNLRHYCILVSDGETYSIENGNKFQALTILVDHYHNKRDGLPCKLEMPVPNPDTRHRVWKEYQQLSTDLTQKPGHKPDQPGSKKPLRPVPPIPLDDCFPVSPSTPTENGACFIAPGSSPSLERPPPHKCWEDEEELEDQDSTQFRSLTVEEAEEQEKIYSDIRLDVMNRGLNPDQVTLSENLGSGQFGEVKKGVCRLMGKDIRVAVKTLKNNDREAESEIMKEAELMKKLDHPHIVRMIGVCKSNSLMLVLELAELGPLKKYLERHKEMRTWHLLELATQICDGMRYLEQHDVVHRDLATRNVLLLTEHFAKISDFGMSRMVDSSEYYLAHVPGKWPLMWYAPECLYYHKFDTKSDVWSFGVTLWEIMSFGARPYAHKKPPQILAFLEEGHRMAKPKDCDDRIYNIMLSCWHFQKEKRPTFAELHPKLNKLFSDFQEH

>BgSYK

MLKKIFRSSKTNEQEEPVIPRVYNEPYFYGRITRQEAEAILEVNGMKDGLFLLRESITPMGNFSLTMAFDKKAVHYAIEKKIDGQFMIAEGLTFRDPIELIEHYQTHKTGFVTVPKIPCQRSPDQDAIAFRGVSYQDLHNHMKATAKNLNASLSQALGAKREAMLMHVVKNMHEKMPWFHGEISREECIRRLETDGHQDGKFIIRSRADGQTFALTMSSDNAMRHYFILVHDGETYSIENGQRFKALIILVDHYHNKRDGLPCKLTVPCPAPETKHQTWERYINLSKNLCKANPANPVRRGSSTSRETSSSRLPLPPRPRPAPPIPPNALGWLNNHHDEEEDELDALPDLPAPVISQEQAEHLESVYIDIRDDVLSNDLRPDQVRLEGKLGSGQFGEVRRGMCYLTSRSNPVPVAVKTLKNNDPAGEKEILSEASLMKQLNHQHIVRMIGVCKGESLMLVMELAELGPLKKYLEKNLDIRVWHQLELMVQICDGMRYLESKDVVHRDLAARNVLLVTDHFAKISDFGMSRMVDNSSAYYLAHGPGRWPLMWYAPECLYFHKFDSKSDVWSFGVTMWEIMSNGSRPYDRKKPQQILHFIESGNRLNKPRNCDDRIYEIMLQCWEFSKEDRPHFQLLKISLEELYREFLRLQI

>LaSYK

MAGVSIPPGTMSYFWGRITREEATTVLRQAGCRDGMYLLRESVNKHGNYALSLCFRDEVHHYNIERQLDGKYQIPDGKKFSGPVELIQHHKTFLDGLLCLPSIPVERGNRPLMVFRGVTQTEFDQVLLKKVSEMGLKGDALANALGPQRFQLFNVVAKDLHKDQCWFHNNIEREEAENRLRNAGHRNGLFLVRAKAGSQGFAMTVSFNNAAKHYKLDLHPSGQLAIEDGPKFNSVMELVDHYYNRADGLLCQLREPCTRPDYRKKNVGATNMDDSPAYWTVPANESEQLYASAEPNGLPEASAGPLPRRNPHSPLPAPPPSEVAPPPRGHSSSNAPLRLPTPKSGPRSPASAAQRSPSRVNHAAMGIAAVHIGGTWDEPEMPERNAPPPPETPNVAEERQKIYDSVPISEEIFNLDITHLKLDEDLGSGAFGAVKKGRYTPPIRGHGYGNAAEIPVAVKTLKANDIPNAREDILKEARIMADLKHKNIVRLIGVSQGETIMLVMELAPLGQLNKFLKGHSIRSFPMNKVMTIMHQVALGMEYLESVRFVHRDLAARNVLLAHEGFAKISDFGMSKAVGANNEYYKAAAAGKWPLKWYAPECIYYFKFDSKSDVWSYGVTLWEATSYGQKPYRGKKGQDILQMIEANQRLEKPPDCPQEIYDIMSLCWKYKKKDRPTFKDLVQKVKPFRH

>HvSyk

MADPSLLRWFHGRITRDIAENCLQTTTSMDDGTYLLRESASEIGSYVLSVCKDKKVIHYAIQRQPSGMVGIADGPQFPGPVELVNHHKTSLGGLMTKLTYPCERPAGVKATAYKGISHDDLDEAIRTVLRTEGVVNEEAVRKFKVKMEYVVGNILHRQQPWFHGVIPRTEAERRLKSLSETGMFLIRERGSPRGSYVIGLFYQGGVYHYLFESNNQGQLSIKSGRSFDNLMAVVNFYSQKSEGLFCTLKRACEVSWFEFQPKTEIYKNILLHPEIQAELKKTLLLYEDELKKYRRVSRVERPPVPNRPVSRVSNQIDVKDTWVYEKIYDSKDIVKKEGFKNPFETQERETELDPKRLRLEHELGHGNFGSVVKGVYLLKNNKSVAVAVKTLKEEDIPGQKSEIVKEAEIMSKLDHPNIVRLIGVTQSPGFMLVMELAPQGPLHKFLKKNRNMNVLDILVLMLQVDEGMNYLESQHFVHRDLAARNVLVVSENFVKISDFGMSRAMGVGNEYYRAERAGKWPLKWYAPECIYYHKFSSKGDVWSYGITMWEAVSYGGKPYQGMRGPVIVEKLESGYRLPKPDNCSDAVYTLMLKCWETNPDDRPSFMEIGIILAQYIQNERVKKK

>AdSYK

MSETKIKQMADPLFQHWFHGRISRQEAEDLLARAGKHDGSYLLRESTASAGSYALSVCHNNKIIHYHIQRHSDGMVAIEDGARFLGPVELVHHHQHALDGLLTKLTEPCNRLPGVPPRTFSGANQEHIKDAAIAAIASMGLEEGDENTATLMRAKLESAIGSVLHKNQVWFHGGIPREEAERRLQVLGSQEGMFLIREKDPGFVLGLVHDGSMVHYLFDVDSQGRLSIKSGPKFDNLMLAVDYYTLREDGLLCKLREPCNAELFDGKRRTPSVGSRPVGLFDNTQRRTPSQSFTEGNPFPGNPFLQGAASPAAMVPDILREAVPRCPPPRDPPPPVPTSLPPPARDRTSGRPRLPPRPPTSPRNASPATSSYPMSAEENQILGAARTSPSKGSQFESIYDSVKMRKSSFYFNQFKDVQTHKLRSENLSLERELGHGNFGSVLKGEYIKQNGEKIPVAVKKLKSEEMNNPKSEIMHEAEVMMRLDHPNIVRIIGICEDSTVMLVMELAPEGPLHKYLKKHKSLPMFKIFVIMLQVAEGMQYLENMQFVHRDLAARNVLVVNEDFVKISDFGMSRAMGTGTDYYRAGKPGKWPLKWYAPECIYFRKFSSKSDVWSYGVTFWEAASYGKKPYDGLNGQYILEQIESGYRLECPEGVPQDVYKIMKSCWEFREEDRPSFQALSQELGTAFYELKSVKGY
