# Supplementary material for: Ancient Origin of the CARD–Coiled Coil/Bcl10/MALT1-Like Paracaspase Signaling Complex Indicates Unknown Critical Functions
Source: Front Immunol. 2018 May 24;9:1136. doi: 10.3389/fimmu.2018.01136 (PMC5978004; doi:10.3389/fimmu.2018.01136)
Supplement: Supplementary file 2 [file Image_1.PDF]

frontiers

Supplementary Material

Ancient co-evolution of the CARD-CC/Bcl10/MALT1-like paracaspase (CBM) signaling complex indicates unknown critical functions.

Jens Staal*, Yasmine Driege, Mira Haegman, Alice Borghi, Paco Hulpiau, Laurens Lievens, Ismail Sahin Gul[2,4], Srividhya Sundararaman, Amanda Gonçalves, Ineke Dhondt, Jorge Pinzón, Bart P. Braeckman, Ulrich Technau, Yvan Saeys, Frans van Roy and Rudi Beyaert

*** Correspondence:** Jens Staal: jens.staal@irc.vib-ugent.be

Supplementary Figures

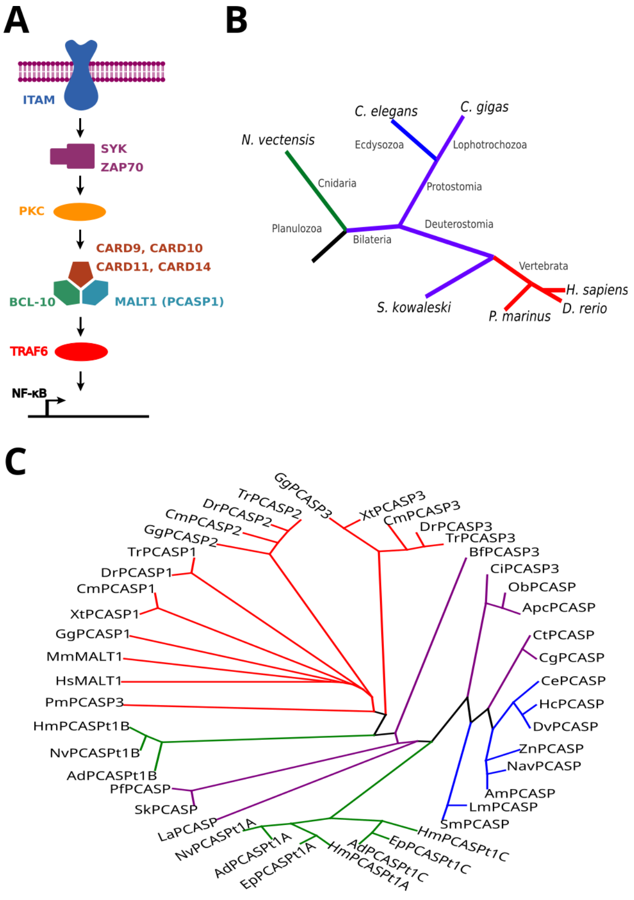

Supplemental figure 1 : Phylogeny of CBM and the type 1 paracaspase N-terminal domains

A) A simplified overview of CBM complex-mediated activation of NF-κB signaling in human and mouse. ITAM : immunoreceptor tyrosine-based activation motif, PKC : Protein kinase C. **B)** Phylogenetic overview of the organisms used in this study and the presence of CARD-CC/Bcl10. Vertebrates highlighted by red branches, bilaterian invertebrate species with Syk/CARD-CC/Bcl10 by purple branches, cnidarian by green branches and species from bilaterian phyla lacking Syk/CARD-CC/Bcl10 (*e.g.* arthropods, nematodes) by blue branches. **C)** Maximum likelihood phylogeny (MUSCLE + PhyML) of the type 1 paracaspase DD-Ig1-Ig2 N-terminal domain, which is likely to be involved in Bcl10-binding. Color coding as in (B) and species key in supplemental data: sequences.

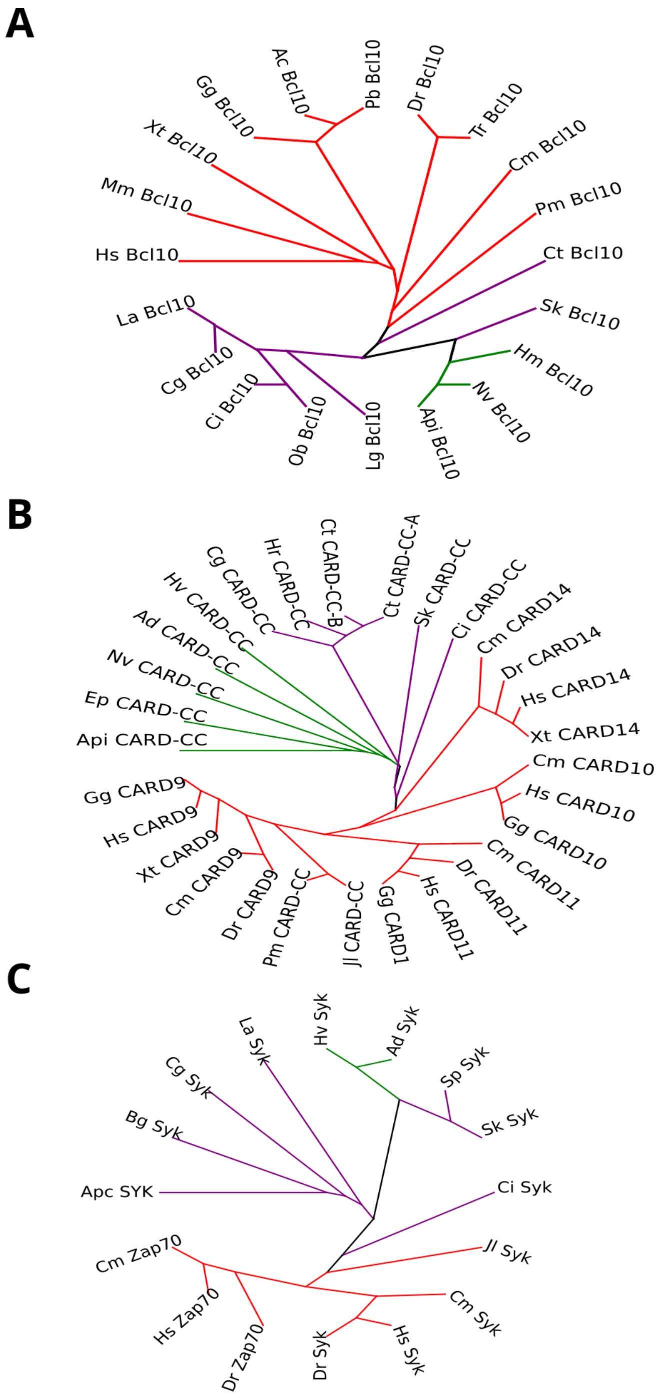

Supplemental figure 2 : Phylogeny of CBM – complex associated proteins

A) Maximum likelihood phylogeny (MUSCLE+PhyML) of vertebrate and invertebrate Bcl10 homologs. **B)** Maximum likelihood phylogeny (MUSCLE+PhyML) showing the relationships between CARD9, the three CARMA paralogs and their invertebrate CARD-CC homologs. **C)** Maximum likelihood phylogeny (MUSCLE+PhyML) of Zap70/Syk homologs. Species key in the “sequences” segment in supplemental data. Red branches highlight vertebrate sequences, green branches cnidarian and purple branches bilaterian invertebrates with Bcl10.

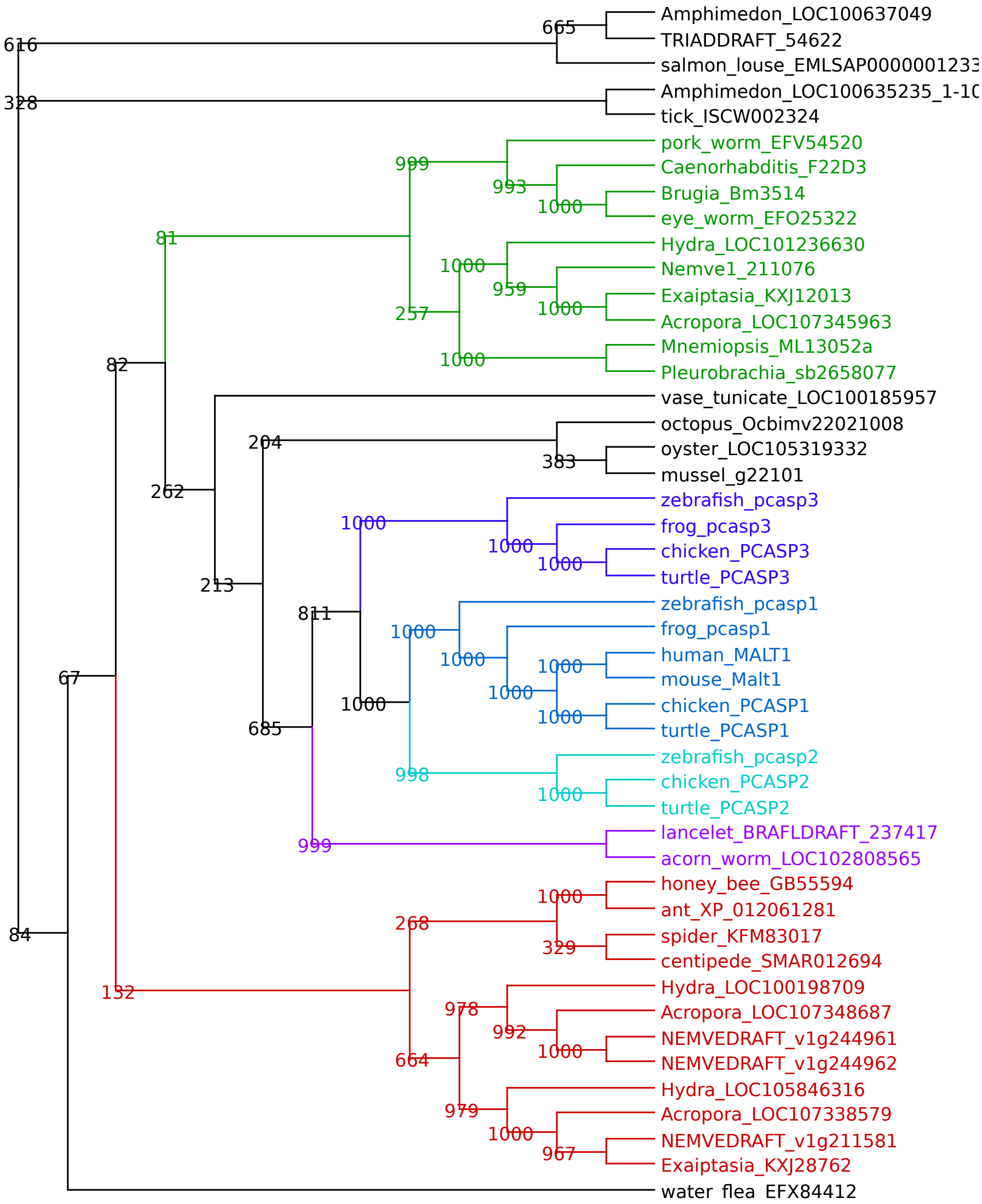

Supplemental figure 3 : Phylogeny of type 1 paracaspases with bootstrap values Phylogenetic analysis of reliable full-length type 1 and type 2 paracaspases revealed 3 major families of type 1 paracaspases in bilaterians : The "arthropod" group (red), the "nematode" group (green) and the mollusk/deuterostome group (several colours). Arthropod and nematode type 1 paracaspases clustered with different type 1 paracaspases from cnidarians, indicating that the last common ancestor of planulozoans already had several type 1 paracaspase paralogs.

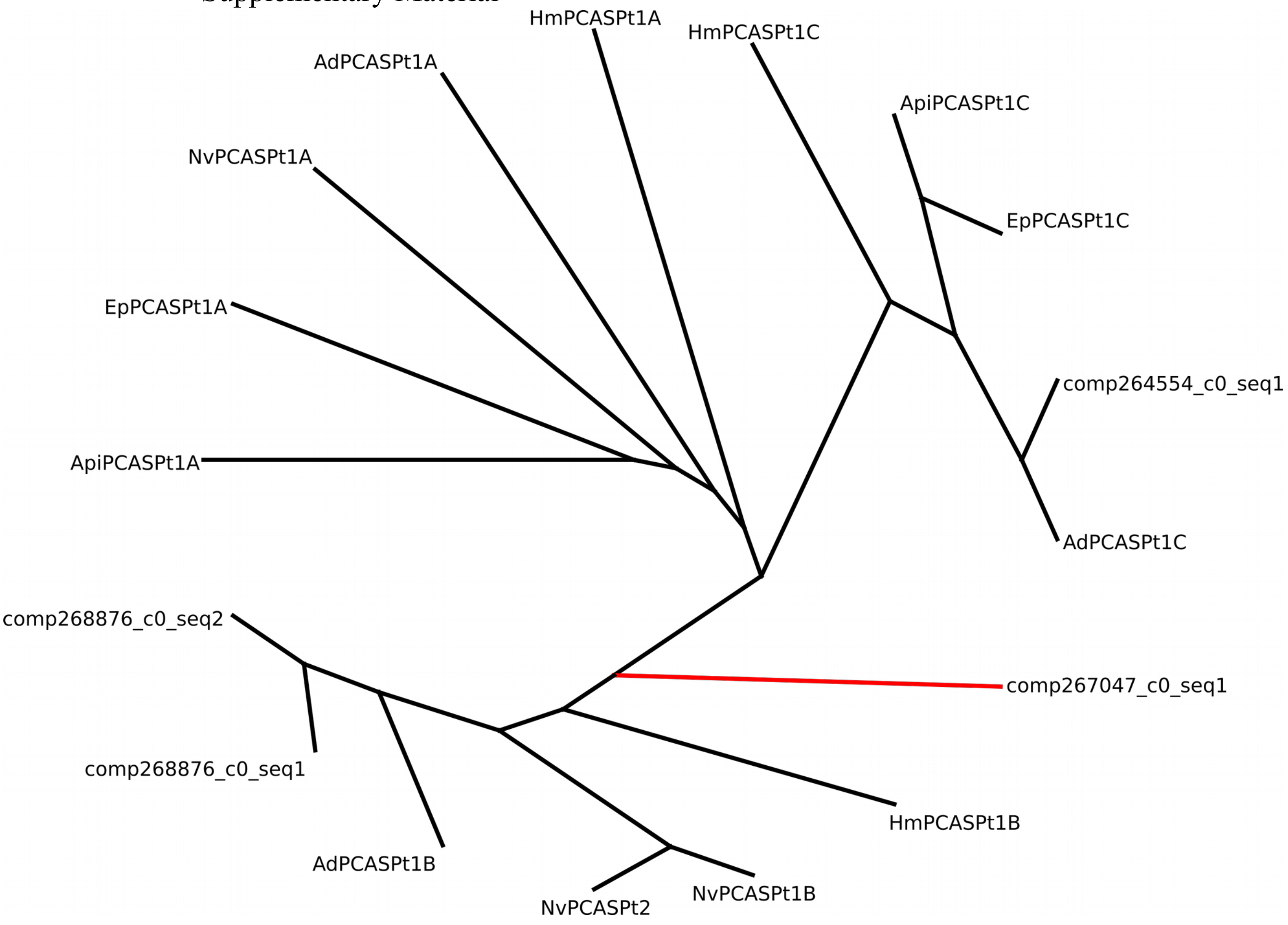

Supplemental Figure 4 : Identification of the coral paracaspase paralog which is differentially expressed during natural coral bleaching stress.

The transcript comp267047_c0_seq1 (indicated in red) is one of the most significantly altered transcripts during and after natural coral bleaching stress (78), a dysregulated coral host immune response triggered by environmental stress leading to detrimential dysbiosis. The protein sequences of the MALT1 homologs identified in the RNAseq experiment were aligned against the other cnidarian paracaspase paralogs with MUSCLE; phylogenetic analysis was done with PhyML in Ugene.
